# Protein composition analysis of polyhedra matrix of *Bombyx mori* nucleopolyhedrovirus (BmNPV) showed powerful capacity of polyhedra to encapsulate foreign proteins

**DOI:** 10.1038/s41598-017-08987-8

**Published:** 2017-08-18

**Authors:** Zhong-Jian Guo, Meng-Han Yu, Xian-Yun Dong, Wei-Li Wang, Ting Tian, Xian-Yin Yu, Xu-Dong Tang

**Affiliations:** 1 Institute of Life Sciences, Jiangsu University, 301# Xuefu Road, Zhenjiang, 212013 Jiangsu P.R. China; 2 Department of Occupation Evaluation, Hebei Ankang Occupation Health Technology Service Co. Ltd., 286# Tianshan Street, Shijiazhuang, 050000 Hebei, P.R. China; 3 College of Biotechnology, Jiangsu University of Science and technology, 2# Mengxi Road, Zhenjiang, 212018 Jiangsu P.R. China

## Abstract

Polyhedra can encapsulate other proteins and have potential applications as protein stabilizers. The extremely stable polyhedra matrix may provide a platform for future engineered micro-crystal devices. However, the protein composition of the polyhedra matrix remains largely unknown. In this study, the occlusion-derived virus (ODV)-removed BmNPV polyhedra matrix fraction was subjected to SDS-PAGE and then an LC-ESI-MS/MS analysis using a Thermo Scientific Q Exactive mass spectrometer. In total, 28 host and 91 viral proteins were identified. The host components were grouped into one of six categories, i.e., chaperones, ubiquitin and related proteins, host helicases, cytoskeleton-related proteins, RNA-binding proteins and others, according to their predicted Pfam domain(s). Most viral proteins may not be essential for polyhedra assembly, as evidenced by studies in the literature showing that polyhedra formation occurs in the nucleus upon the disruption of individual genes. The structural role of these proteins in baculovirus replication will be of significant interest in future studies. The immobilization of enhanced green fluorescent protein (eGFP) into the polyhedra by fusing with the C-terminus of BM134 that is encoded by open reading frame (ORF) 134 suggested that the polyhedra had a powerful capacity to trap foreign proteins, and BM134 was a potential carrier for incorporating proteins of interest into the polyhedra.

## Introduction

Baculoviridae is a family of insect DNA viruses that have a large, circular, supercoiled and double-stranded DNA genome within a rod-shaped nucleocapsid^1^. During the life cycle of baculovirus, two typical progeny virions, i.e., budded virus (BV) and occlusion-derived virus (ODV), are produced. These virion phenotypes are genetically identical but differ structurally and functionally. BVs are responsible for cell-to-cell infections in cultured cells and tissues of susceptible hosts, while ODVs are required for horizontal insect-to-insect spreading^1, 2^. ODVs are embedded in a protein crystal called occlusion body (OB)^3^. Once ingested by a susceptible larva, the OBs are quickly dissolved due to the alkaline pH of the host midgut. ODVs are released to cross the peritrophic membrane and initiate the primary infection^4^. During the early stage of infection, nucleocapsids are transported through the nuclear membrane and migrate across the cytosol to the cell membrane for budding, thereby producing extracellular BVs. During the very late stage of infection, nucleocapsids in the nucleus are enveloped, resulting in ODVs that are packaged into the crystalline polyhedra matrix to form OBs.

The OBs, also named polyhedra, are stable and resistant to most normal environmental conditions due to the protective effect of multiple layers that are formed mainly by the polyhedrin envelope protein (PEP)^5^, thereby allowing the ODV virions to remain infectious in soil indefinitely^1^. Polyhedrin, a highly expressed protein with a molecular weight of ~29 kDa, is the major component protein in the polyhedra matrix. Polyhedrin is one of the most conserved proteins of alpha-baculovirus. Certain single point mutations in polyhedrin result in phenotypic changes, such as large, cuboid polyhedra that occlude no or few ODVs^6–9^. Polyhedra without ODVs have potential applications as stabilizers of proteins of interest, such as enzymes and growth factors, that are encapsulated in the polyhedra matrix^10, 11^. To explore the use of polyhedra as a framework for future engineered micro-crystal devices, an analysis of the component proteins associated with the polyhedra matrix is required.

The availability of genome sequences facilitates component analyses of baculoviruses on a proteomic scale. To date, the ODV components of eight baculoviruses, i.e., *Autographa californica* multiple nucleopolyhedrovirus (AcMNPV)^12^, *Culex nigripalpus* nucleopolyhedrovirus (CuniNPV)^13^, BmNPV^14^, *Chrysodeixis chalcites* nucleopolyhedrovirus (ChchNPV)^15^, *Helicoverpa armigera* nucleopolyhedrovirus (HaNPV)^16, 17^, *Anticarsia gemmatalis* multiple nucleopolyhedrovirus (AgMNPV)^18^, *Mamestra brassicae* nucleopolyhedrovirus (MabrNPV)^19^, *Pieris rapae* granulovirus (PrGV)^20^ and *Clostera anachoreta* granulovirus (ClanGV)^21^, and the BV components of four viruses, i.e., AcMNPV^22^, HaNPV^17^, AgMNPV^18^ and MabrNPV^19^, have been reported. These data shed light on the structure and assembly of baculovirus virions; however, in comparison, little is known regarding the structural composition of the polyhedra matrix.

In this study, a comprehensive analysis of the BmNPV polyhedra matrix-associated component proteins was performed using a Thermo Scientific Q Exactive mass spectrometer. In total, 28 host and 91 viral proteins were identified to be associated with the polyhedra matrix of BmNPV. Most of these viral proteins may not be essential for polyhedra assembly, as evidenced by studies in the literature that polyhedra formation in the nucleus was shown to occur upon the disruption of individual genes. The host components had been grouped into one of six categories, i.e., chaperones, ubiquitin and related proteins, host helicases, cytoskeleton-related proteins, RNA-binding proteins and others, according to their predicted Pfam domain(s). These host-derived proteins may be important for the assembly and maturation of polyhedra. These findings may provide novel insights into the baculovirus structure and shed light on the powerful ability of polyhedra to encapsulate foreign proteins.

## Results and Discussion

### SDS-PAGE analysis of ODV-removed polyhedra matrix

**Figure 1:**
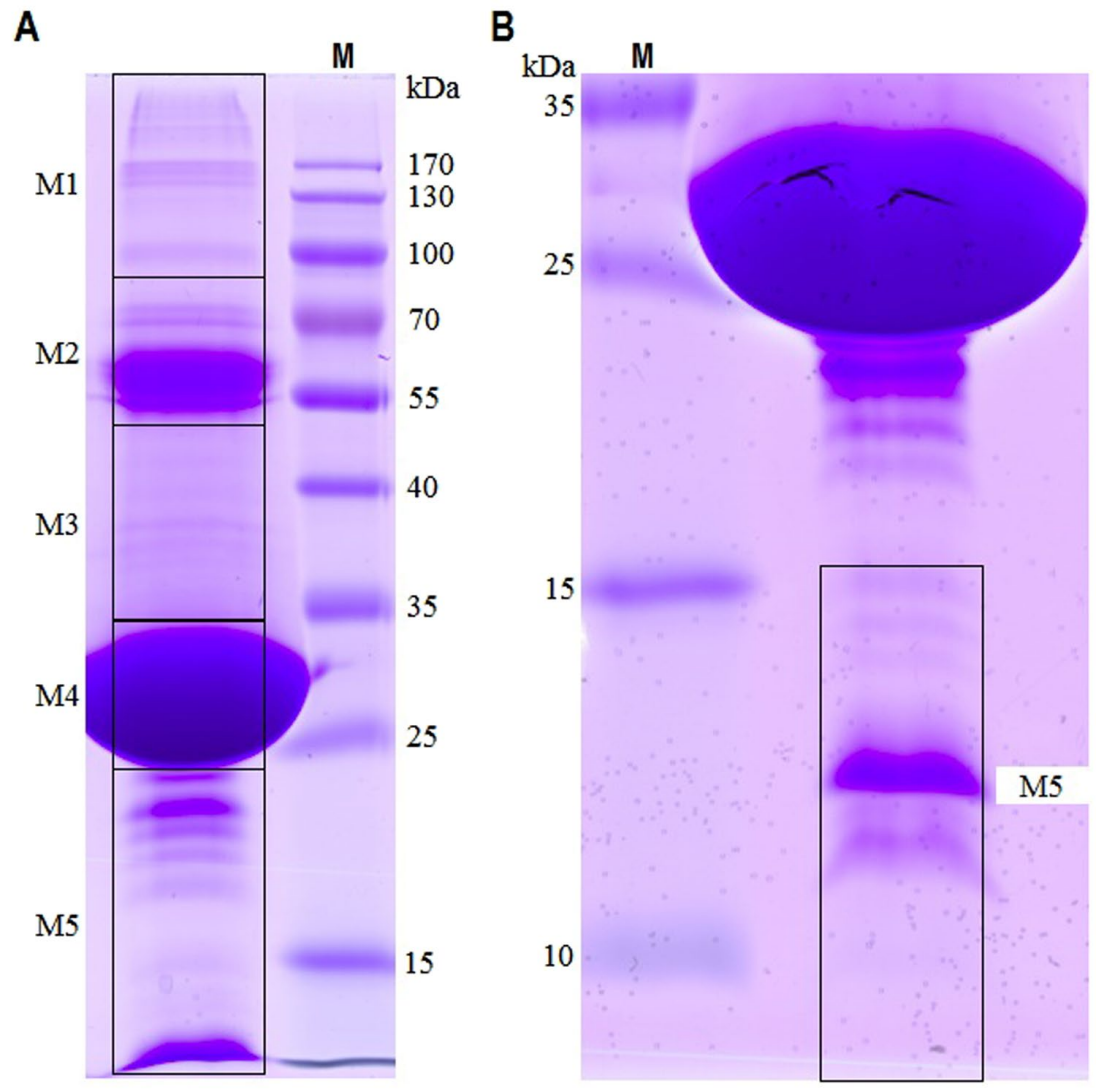
BmNPV polyhedra matrix proteins were separated on 12% (**A**) and 15% (**B**) SDS-PAGE gels. Purified polyhedra were treated with an alkaline solution. Undissolved polyhedra were removed by low-speed centrifugation at 500 × g. The resulting supernatant was collected and then centrifuged by continuous sucrose gradients to remove the ODVs. Protein sample over the upper gradient was separated by SDS-PAGE. Proteins on the gel were excised into five contiguous sections (M1 to M5) and subjected to in-gel digestion and LC-ESI-MS/MS analysis. Two M5 sections shown in (**A**) and (**B**) were combined for the determination. Lane M, protein marker.

To identify proteins associated with the BmNPV polyhedra matrix, polyhedra from infected cells were purified, and the virions were released and pelleted by continuous sucrose centrifugation. The ODV pellet and sample over the top gradient were collected for the SDS-PAGE analysis and then made visible using Coomassie brilliant blue staining. The results showed that certain protein bands were present in both fractions (Supplementary Fig. S1), which is consistent with previous observations in an earlier proteomic investigation of CuniNPV ODV^13^. The total polyhedra matrix proteins on the gel were divided into 5 regions (M1 to M5) (Fig. 1) and subjected to an in-gel trypsin digestion and LC-ESI-MS/MS analysis using a Thermo Scientific Q Exactive mass spectrometer. A comprehensive compilation of the data was provided in supplementary Tables S1 to S9.

### Identification of host proteins associated with the BmNPV polyhedra matrix

Certain viruses trap the host proteins in their virions either specifically or randomly. An earlier study has shown that baculoviruses can trap heterologous chloramphenicol acetyltransferase in BV particles nonspecifically^23^. In this present study, more than 350 host proteins were identified to be associated with the BmNPV polyhedra matrix, which is indicative of the powerful ability of polyhedra to encapsulate foreign proteins. Twenty-eight of these identified proteins that had more than 1 peptide were listed and grouped, according to their predicted Pfam domain(s), into one of six categories, i.e., chaperones, ubiquitin and related proteins, host helicases, cytoskeleton-related proteins, RNA-binding proteins and others (Table 1). These proteins may play a structural role in the long-term stability of polyhedra in most environmental conditions or participate in the polyhedra assembly and maturation and then became trapped in the polyhedra.

**Table 1:** Host proteins identified in the BmNPV polyhedral matrix using the LC-ESI-MS/MS Q Exactive technique.

Classification	Protein	Pfam domain(s)	Abbreviation	Size (amino acids)	Gel region(s) (No. of peptides identified; Coverage (%))
Chaperones	BGIBMGA002381-PA	70 kilodalton heat shock cognate/protein	HSP70-4	649	M1 (5; 10.63), M2 (16; 34.98), M3 (8; 18.03)
	BGIBMGA006196-PA	40 kilodalton heat shock protein	HSP40	313	M3 (3; 13.74)
	BGIBMGA002429-PA	Cyclophilin type peptidyl-prolyl cis-trans isomerase/cyclophilin-like domain	Cyclophilin A	165	M5 (6; 58.18)
	BGIBMGA004331-PA	FK506 binding protein	FKBP	108	M5 (2; 32.41)
Ubiquitin and related proteins	BGIBMGA011805-PA	Ubiquitin carboxyl-terminal hydrolase	UCH	665	M1 (1; 4.36), M2 (1; 2.56)
	BGIBMGA011581-PA	Small ubiquitin-related modifier	SUMO	91	M1 (1; 13.19), M2 (1; 13.19), M3 (1; 13.19)
	BGIBMGA001549-PA	Ubiquitin	Ubiquitin	153	M3 (3; 26.80), M5 (3; 26.80)
Helicases	BGIBMGA011746-PA	DEAD/DEAH box helicase	DEAD/H	569	M1 (3; 7.56), M2 (6; 16.52), M3 (11; 26.89)
	BGIBMGA011965-PA	DEAD/DEAH box helicase	DEAD/H	628	M2 (5; 13.69), M3 (8; 20.70)
	BGIBMGA010673-PA	DEAD/DEAH box helicase	DEAD/H	372	M3 (9; 33.60)
	BGIBMGA004822-PA	DEAD/DEAH box helicase	DEAD/H	405	M3 (4; 15.31)
Cytoskeleton- related proteins	BGIBMGA005576-PA	Actin	Actin	344	M1 (4; 23.84)
	BGIBMGA002981-PA	Profilin	Profilin	126	M5 (3; 47.62)
	BGIBMGA007092-PA	Calponin homology domain, calponin family repeat	CH	173	M5 (6; 54.91)
RNA binding proteins	BGIBMGA006405-PA	RNA recognition motif	RRM	361	M3 (5; 15.80)
	BGIBMGA014155-PA	K Homology domain	KH	377	M3 (6; 27.98)
	BGIBMGA004881-PA	RNA recognition motif	RRM	252	M3 (4; 23.02), M5 (3; 17.86)
	BGIBMGA013464-PA	RNA recognition motif	RRM	287	M3 (3; 15.33)
	BGIBMGA007410-PA	RNA recognition motif	RRM	314	M3 (3; 15.61)
	BGIBMGA013100-PA	Like Sm protein	LSm	130	M5 (2; 23.85)
Others	BGIBMGA003126-PA	Nup93/Nic96	NIC	820	M1 (3; 5.24),
	BGIBMGA000808-PA	XRN 5’-3′ exonuclease	XRN_N	322	M1 (2; 8.39),
	BGIBMGA005650-PA	CAF1 family ribonuclease		479	M1 (3; 8.77), M2 (3; 8.77),
	BGIBMGA008447-PA	Nuclear transport factor 2 domain, Nuclear RNA export factor 1	NTF2 NXF1	613	M2 (2; 4.89),
	BGIBMGA001106-PA	Ribosomal protein L21e		159	M5 (3; 24.53)
	BGIBMGA006867-PA	Ribosomal protein S8		129	M5 (2; 23.26)
	BGIBMGA011282-PA	Ribosomal S13/S15 N-terminal domain		151	M5 (2; 19.21)
	BGIBMGA009106-PA	glutathione S-transferase 2	GST-2	416	M5 (4; 13.22)

**Figure 2:**
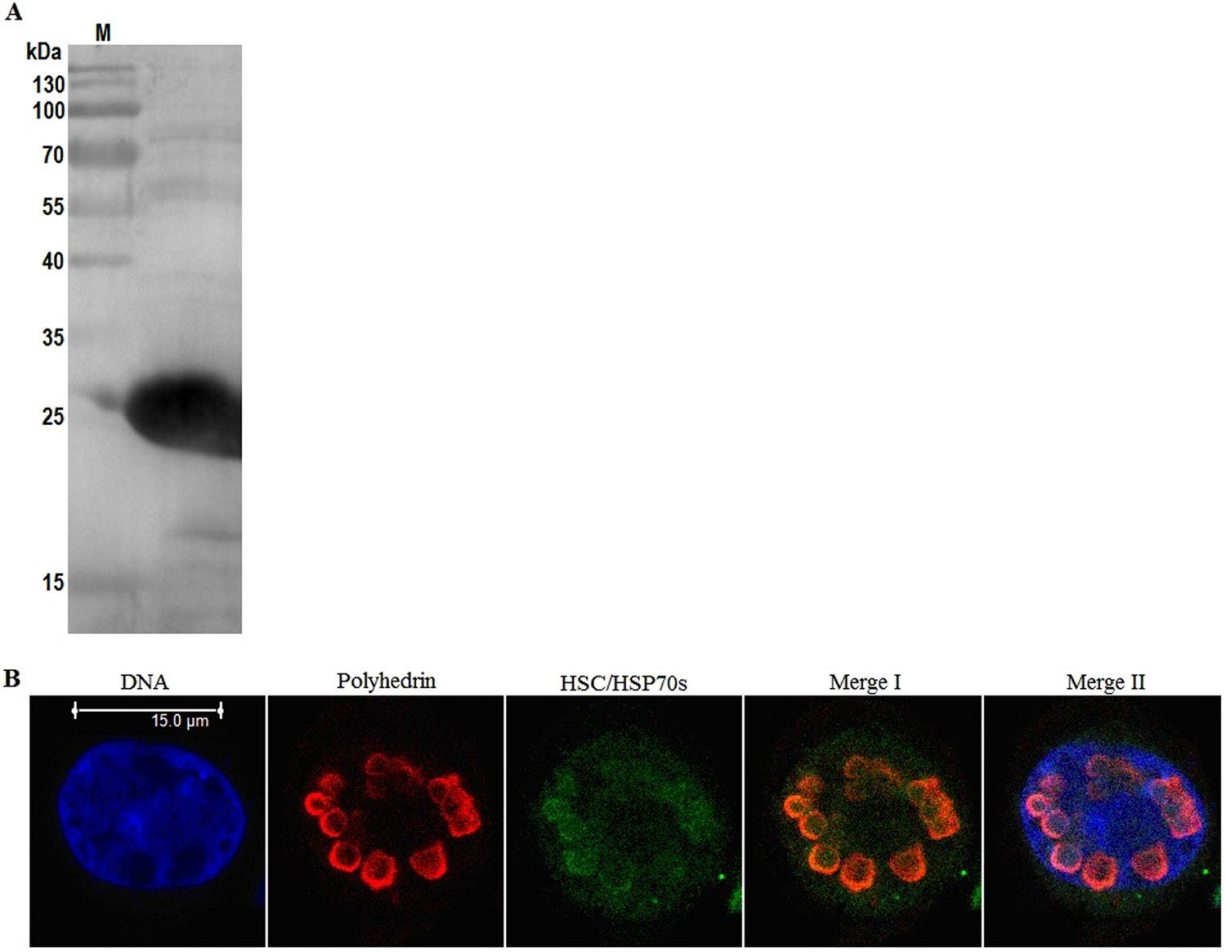
Host protein HSP70-4 was associated with the BmNPV polyhedra matrix. (**A**) Western blot analysis of HSP70-4 in the BmNPV polyhedra matrix. The fraction over the upper sucrose gradient was loaded for the Western blot analysis. Images were visualized using a chemiluminescence detection system. Lane M, the protein marker. (**B**) Co-localization of polyhedrin with HSP70-4 in the polyhedra in infected BmN cells. Cells were infected with the BmNPV T3 isolate at an MOI of 10 TCID_50_/cell. At 48 h p.i. the cells were fixed, permeabilized, and then incubated in a buffer containing mouse monoclonal anti-polyhedrin and rabbit polyclonal anti-HSC/HSP70 antibodies. Next, the cells were stained with the Texas Red-conjugated goat anti-mouse IgG, FITC-conjugated goat anti-rabbit IgG and Hoechst 33342, washed with 1× PBS, and then imaged under a Leica TCS SP5 confocal laser scanning microscope using the Texas Red filter set for the Texas Red-conjugated antibody and the FITC filter set for the FITC-conjugated antibody. In the merged images, yellow suggested an association between polyhedrin and HSP70-4 in the polyhedra matrix (Merge I), and magenta indicated the nuclear location of the polyhedra (Merge II).

During viral infections, many viral encoded proteins are synthesized in large amounts in a relatively short time and protein folding can become a limiting step during their active conformation and trafficking. Therefore, on the one hand, viruses require cellular chaperones for their protein folding processes; on the other hand, because chaperones are involved in the regulation of fundamental cellular processes, viruses must interact with chaperones^24^. The HSC/HSP70 family chaperones, which are central components of the cellular chaperone network, are involved in all stages of the viral life cycle of replication^25, 26^. In the case of baculovirus, the expression of HSP70 is often induced upon viral replication *in vivo* and *in vitro* ^27^. HSP70 plays a role in facilitating the genome synthesis of AcMNPV and the release of progeny BVs^28^, and is identified as a component of BV and ODV of BmNPV^14, 29^, BV of HaNPV^17^, and ODV of AgMNPV^18^. In this report, HSP70–4 was identified by an LC-ESI-MS/MS analysis to be associated with the BmNPV polyhedra matrix. The association between HSP70-4 and the polyhedra matrix was further confirmed by a Western blot analysis using a rabbit polyclonal anti-HSC/HSP70 antibody, which showed certain bands in five excised regions of the gel (Fig. 2A), and an immunofluorescence microscopy assay, which showed co-localization of HSP70-4 and polyhedrin in the polyhedra in the nucleus of infected cells (Fig. 2B). These results suggested that HSP70-4 was a component protein of the BmNPV polyhedra matrix. In addition to HSP70-4, this study identified HSP40 and two chaperone-like proteins, i.e., cyclophilin A and FKBP, to be associated with the BmNPV polyhedra matrix (Table 1). HSP40, which is a co-chaperone of HSP70, is involved in the enhancement or inhibition of pathogenesis in a wide range of viral infections^30^. Cyclophilin A has been shown to be recruited specifically into the virion for the infectivity of human immunodeficiency virus-1^31, 32^. For baculovirus HaNPV, cyclophilin A is identified to be associated with virion ODV and BV^17^. FKBP is also a component of the HaNPV ODV^17^. However, to date, their roles in baculovirus infections are not investigated yet.

**Figure 3:**
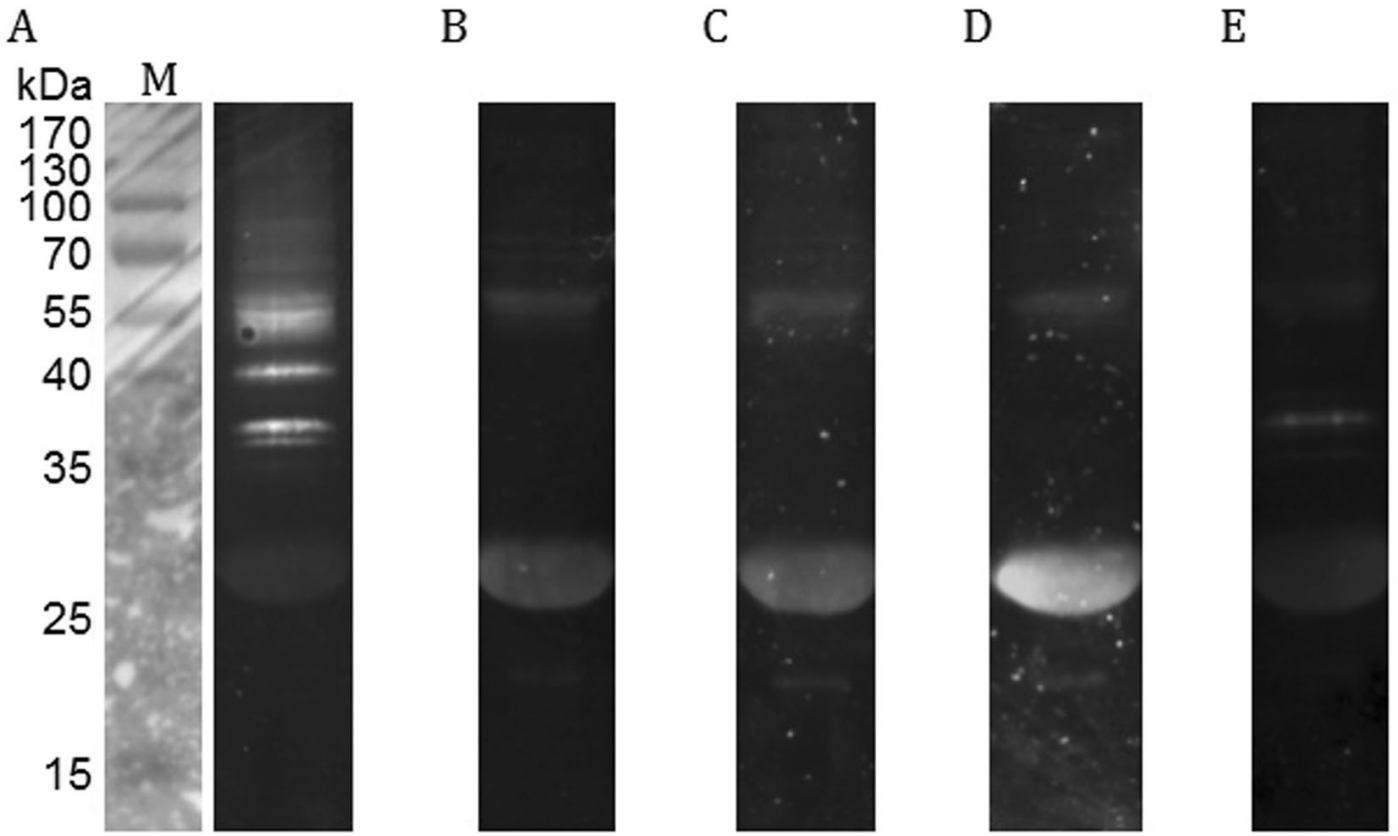
Western blot analysis of ubiquitylated proteins associated with the polyhedra matrix of BmNPV. Polyhedra matrix proteins were separated on SDS-PAGE gel and then analyzed using mouse monoclonal anti-ubiquitin antibody clone FK2 (**A**) and FK1 (**B**), rabbit monoclonal anti-ubiquitin antibody Lys48-specific clone Apu2 (**C**) and Lys63-specific clone Apu3 (**D**), and mouse monoclonal anti-linear poly-ubiquitin antibody clone LUB9 (**E**). Images were visualized using a secondary antibody conjugated to HRP and a chemiluminescence detection system.

Ubiquitin is a well-conserved protein that contains 76 amino acids and plays important roles in many diverse cellular activities^33^, and certain viral processes, such as virus entry and egress^34^. The ubiquitin-proteasome pathway plays a central role in BmNPV infections since its inhibition by the proteasome inhibitor MG-132 results in reduced BV and ODV formation^35^. The inhibitor bortezomib also leads to similar observations in terms of a reduced progeny BV production, restricted ODV proliferation, fewer copies of viral genomic DNA replication and a decreased number of *ie1* and *polyhedrin* transcripts^36^. Ubiquitin homologs are found in most lepidopteran baculovirus genomes. ORF26 in the BmNPV genome encodes a ubiquitin homolog protein. During the very late stage of polyhedra formation, the BmNPV ubiquitin is largely and evenly localized in the nucleus, and in the cytoplasm, it exhibits a dotted distribution^37^. The identification of host and viral ubiquitins (Tables 1 and 2) prompted the question of whether certain proteins that are associated with the BmNPV polyhedra matrix were ubiquitylated. To address it, the polyhedra matrix sample was subjected to a Western blot analysis using antibodies specific to different forms of ubiquitin. Both the FK2 and FK1 antibodies recognize poly-ubiquitin conjugates, but the FK2 antibody also recognizes conjugated mono- and multiple mono-ubiquitin. Certain bands were observed by immunostaining with the FK2 antibody (Fig. 3A). When the sample was analyzed using the FK1 antibody, several bands were detected (Fig. 3B). These results demonstrated that certain mono- (and/or multiple mono-) and poly-ubiquitinated proteins were present in the BmNPV polyhedra matrix. Generally, proteins conjugated to poly-ubiquitin chains linked at the K48 residue of ubiquitin are targeted for degradation by the proteasome^145^, whereas proteins that are conjugated to K63-linked poly-ubiquitin chains play other roles, such as the regulation of protein function and cellular trafficking^146^. Staining with the K48- and K63-specific antibodies revealed a ubiquitination profile that was similar to that observed using the FK1 antibody (Fig. 3C, D). The LUB9 antibody recognizes the head-to-tail linear poly-ubiquitin chain in which the C-terminal Gly of one ubiquitin monomer was conjugated to the N-terminal Met of the next monomer. After staining with the LUB9 antibody, a visible band was detected (Fig. 3E). Thus, the differences observed in the staining using several antibodies were interpreted as mono- and/or multiple mono-ubiquitination.

**Table 2:** Viral proteins identified in the BmNPV polyhedral matrix by a Q Exactive LC-ESI-MS/MS analysis.

BmNPV ORF	AcMNPV ORF	Protein designation	Predicted molecular mass (kDa)	Location in ODV virion^†^	Location in BV virion^†^	OB formation when deleted? (ODV embedded?)	Gel region(s) (No. of peptides identified; Coverage (%))
1	8	Polyhedrin	28.8	ENC^17^	NC^17^	No	M1 (18; 64.9), M2 (28; 97.55), M3 (27; 97.55), M4 (25; 97.55), M5 (23; 96.33)
2	9	P78/83	60.9	NC^38, 39^, ENC^17^	NC^17, 38, 39^		M2(6; 16.42), M3 (3; 9.23), M5 (1; 2.40)
3	10	PK1	32.4	NC^40^	NC^40^	No^41^	M3 (1; 4.36)
4	11		39.8			Yes(No)^42^	M1 (1; 4.12), M3 (9; 36.47)
5	13		39.3			Yes(Yes)^43^	M4 (1; 2.42)
7	15	EGT	57.0			Yes(Yes)^44^	M5 (1; 2.77)
8	16	BV/ODV-E26	26.2	ENC^45^	ENC^45^	Yes(Yes)^44^	M5 (1; 6.11)
9	17		24.1		ENC^17^	Yes(Yes)^44, 46^	M5(2; 14.29)
10	18		41.5			Yes(Yes)^47^	M1 (1; 5.06), M3 (6; 24.44)
11	19		12.5				M2 (1; 15.45)
14	23	F protein	78.0	E^12^	E^17, 22^	Yes(Yes)^48, 49^	M1 (1; 2.23)
15	24	PKIP	19.4		ENC^17^	No^50^	M5 (7; 49.70)
17	26		14.5	Unknown^17^			M1 (1; 9.30), M5 (7; 25.58)
19	28	LEF-6	20.3		Unkown^17^		M5 (1; 6.36)
22a							M3 (1; 20.37)
23	31	SOD	16.3	E^17^		Yes(Yes)^51^	M1 (2; 19.87), M3 (2; 15.23), M5(5; 53.64)
26	35	Ubiquitin	8.7	E^17, 52^	E^17, 22, 52^	Yes^35^	M1 (2; 32.47), M2 (1; 20.78), M3 (3; 49.35), M5(2; 44.16)
27	36	PP31	31.5		Unknown^22^	Yes(Yes)^53^	M1 (1; 5.78), M2 (1; 5.42)
29	38		25.5		E^54^		M1 (1; 3.69), M3 (1; 4.15), M5 (1; 5.07)
30	39	P43	43.3			Yes^55^	M2 (1; 4.14), M3 (1; 4.14)
34	43		9.0			Yes, but few^56, 57^	M3 (1; 19.23), M5(6; 61.54)
36	45		22.5				M3 (1; 5.18)
37	46	ODV-E66	79.2	E^12, 17, 22, 58^		Yes(Yes)^59^	M1(10; 21.08), M2(21; 49.29), M3(17; 41.60), M5(5; 13.25)
38	47	ETS/TRAX	10.5				M5 (1; 12.36)
40	51		37.8	ENC^17^	Unknown^22, 60^		M3 (3; 13.48)
43	54	VP1054	42.0	NC^17^	NC^61^	Yes(No)^62, 63^	M3 (2; 7.12)
47	58/59	ChaB	37.8	NC^17, 64^	NC^17^		M5(4; 46.2)
48	60		9.7				M1 (1; 12.05), M3 (1; 12.05), M5(2; 13.25)
49	61	FP25K	25.3	NC^65^, ENC^17^	NC^17, 65^	Yes, but few(Yes, but few)^66–68^	M5 (1; 8.88)
51	63		18.5		E^69^		M4 (1; 5.81)
53	65	DNAPOL	114.4	Unknown^17^		No^70^	M1 (1; 2.64)
54	66		93.3	ENC^17^, E^71^	NC^17^	Yes^72^	M1(8; 15.16), M2(2; 3.85)
55	67	LEF-3	44.9	Unknown^16^			M2(1; 3.90), M3(6; 20.26), M4(1; 3.38), M5 (1; 2.60)
57	69		30.4			Yes(Yes)^73^	M5 (1; 7.63)
59	73		11.5		Unknown^22^	Yes(Yes)^74^	M5(4; 58.59)
60	74		31.0	Unknown^12^	E^17^		M3 (1; 3.36)
61	75		15.5	E^17^	ENC^17^		M5 (5; 35.34)
62	76		9.6	E^75^	E^75^	Yes(No)^76^	M1 (1; 15.29), M5(2; 16.47)
63	77	VLF-1	44.3	ENC^17^	NC^77^	Yes, but few (No)^62, 78^	M1 (6; 17.41), M2 (3; 9.50), M3 (8; 26.65), M5(4; 12.93)
65	79		12.2	Unknown^12^		Yes(Yes)^79^	M5 (2; 17.31)
66	80	GP41	44.9	ENC^17^	ENC^17^	Yes^62^	M1 (12; 41.69), M2 (6; 17.12), M3 (15; 59.31), M4(1; 6.20), M5(2; 9.18)
67	81		27.0	Unknown^17^		Yes(No)^80^	M2 (1; 4.27), M5 (1; 5.56)
68	82	TLP	20.1		E^81^	Yes(Yes)^81^	M2 (2; 25.41), M4(1; 4.42)
69	83		95.8	E^17^, ENC^82^		Yes(No)^83, 84^	M1 (2; 4.53), M2 (2; 4.77), M5 (1; 2.26)
70	87	VP15	15.1				M1 (1; 17.46), M2 (1; 17.46), M3 (1; 17.46)
71	88	CG30	30.7	Unknown^12^		Yes(Yes)^85, 86^	M1 (1; 5.24), M3 (1; 6.37)
72	89	VP39	39.3	NC^17, 22, 87^	NC^17, 22, 87^		M1 (1; 4.00), M3 (4; 20.29), M5 (1; 4.00)
75	92	P33	30.9	ENC^17, 88^	ENC^89^	Yes(No and singly enveloped nucleocapsid)^88^	M1(7; 22.01), M2(7; 32.05), M3(8; 28.96), M4(16; 77.22), M5(14; 62.93)
76	93			NC^90^	ENC^90^	Yes(No)^90^	M5(4; 32.92)
77	94	ODV-E25	25.6	E^17, 22, 91^	E^17, 22^	Yes(No)^92^	M1 (2; 15.35), M2 (1; 7.89), M3 (3; 28.07), M5(10; 60.96)
78	95	DNA helicase/P143	143.6	Unknown^12, 17, 22^			M2 (1; 0.65), M5 (1; 1.15)
79	96	PIF-4	21.0	E^17, 93–95^	E^95^	Yes^95^	M5 (1; 8.79)
80		BRO-b	27.5				M2 (1; 4.18)
81		BRO-c	35.9				M4(1; 2.83), M5 (2; 7.55)
82	98	38 K	38.0	NC^17, 96^	NC^17, 96^	Yes(No)^97^	M3 (3; 11.88)
84	100	P6.9	8.1	NC^17, 98^	NC^17, 98^	Yes(No)^99^	M1 (1; 12.31)
85	101	BV/ODV-C42	41.6	NC^17, 100^	NC^17, 101^		M3 (5; 21.55), M5 (1; 1.93)
87	103	P48	45.4		ENC^17^	Yes(No)^102^	M1 (5; 12.66), M2 (3; 10.34), M3 (12; 39.02), M5 (2; 5.17)
88	104	VP80	79.9	ENC^17^, NC^103, 104^	NC^103, 104^		M1 (9; 19.65), M2 (10; 23.12), M3 (7; 15.75)
91	108		11.8	E^105, 106^		Yes(Yes)^106^	M3 (1; 27.62)
92	109	ODV-EC43	45.0	ENC^17, 107^	ENC^108^	Yes(No)^108–110^	M1 (5; 18.41), M3 (14; 53.96), M5(4; 12.79)
92a			6.8				M2 (1; 47.46)
93	111		8.2				M1 (1; 20.09), M5 (1; 14.93)
94	114		49.4	Unknown^12, 14, 111^	Unknown^22^	Yes(Yes)^112^	M1 (3; 10.14), M3 (11; 39.86), M5(3; 12.74)
95	115	PIF-3	23.0	E^17, 113^		Yes(Yes)^114, 115^	
95a	116						M1 (1; 25.00)
98	120		9.5				M5 (1; 13.41)
100	123	PK2	26.0				M5 (6; 28.89)
104	127	V-cath	36.9		ENC^116^		M1 (1; 3.10), M5 (4; 17.96)
106	129	P24	21.8	NC^17, 117^	NC^17, 117^		M1 (6; 37.44), M2 (5; 46.15), M3 (8; 58.97), M4 (2; 11.28), M5 (15; 73.33)
107	130	GP16	12.1			Yes(Yes)^118^	M5 (1; 10.38)
108	131	PEP	35.4	NC^17^	NC^17^	Yes(Yes)^119^	M1 (5; 16.51), M2 (2; 9.52), M3 (7; 32.70), M4 (3; 19.68), M5(5; 26.03)
109	132		25.2	NC^120^	NC^120^	Yes(Few)^120^	M4 (1; 4.09)
110	133	ALK-EXO	48.5	Unknown^12^			M3 (1; 1.67)
110a							M3 (1; 28.07)
111						Yes(Yes)^121^	M3 (1; 20.00)
112	135	P35	34.5			Yes(Yes)^53, 122^	M3 (6; 24.08), M5(2; 11.04)
114	137	P10	7.5			Yes(Yes)^119, 123–125^	M5 (1; 27.14)
115	138	P74/PIF-0	74.0	E^12, 17, 126^		Yes(Yes)^115, 126^	M5 (1; 1.71)
116	139	ME53	52.6	NC^127^	NC^127, 128^	Yes^129^	M1 (1; 2.00), M3 (6; 19.73), M5 (1; 2.00)
117	141	EXON0	30.1	Unknown^130^	NC^130^	Yes^131^	M3 (1; 4.21), M5 (1; 5.36)
118	142		55.5	ENC^17^	NC^101, 132^	Yes(No)^132, 133^	M1 (8; 21.22), M2 (15; 47.90), M3 (16; 41.06), M5(2; 4.2)
119	143	ODV-E18	10.4	E^17, 22, 134^	E^17, 22^	Yes^135^	M5 (1; 23.76)
120	144	ODV-EC27	33.5	ENC^134^	NC^101^		M3 (7; 31.03)
121	145		11.0	Unknown^136^	Unknown^136^	Yes(Yes)^136^	M5 (1; 22.11)
122	146		22.9	Unknown^137^	NC^137^	Yes^137^	M5(4; 28.36)
124	148	ODV-E56/PIF-5	41.3	E^17, 138^	Unknown^22^	Yes(Yes)^139, 140^	M1 (1; 5.33), M3 (5; 24.00), M5(3; 9.6)
125	149		12.3				M5 (1; 27.36)
128	153	PE38	36.1			Yes(Yes)^141^	M5 (1; 3.24)
129	154		8.9				M3 (3; 66.23), M5 (3; 67.53)
130	1	PTP	19.3	Unknown^142^	ENC^143^	Yes(Yes)^142^	M5 (1; 9.52)
131	2	BRO-d	40.1				M2 (2; 5.73)
134	5		12.4	Unknown^144^			M1(3; 40.37), M2(2; 32.11), M3(2; 32.11), M4(3; 40.37), M5(10; 72.38)

^†^Proteins were reported to be envelope (E)-, envelope-and-nucleocapsid (ENC)-, and/or nucleocapsid (NC)-associated components of BV and ODV in the literature.

The LC-ESI-MS/MS analysis identified several ubiquitinated viral proteins, i.e., BV/ODV-C42, DNAPOL and PKIP, although this results should be further confirmed experimentally (Table 3). Ubiquitin exists in several linked forms, and each form plays distinct roles in the cell. Although BmNPV ubiquitin contains an additional and seven conserved lysines^37^, two linked forms, i.e., K48- and K63-linked poly-ubiquitin chains, were identified, whereas for the host ubiquitin, K6-, K11-, K29-, K48- and K63-linked forms were observed (Table 3). Notably, the identification and role exploration of these ubiquitinated proteins in baculovirus replication will be of interest in future investigations.

**Table 3:** Ubiquitylated proteins and their identified ubiquitylation site(s).

Protein	Protein designation	Ubiquitylation site(s)
BmNPV ORF85	BV/ODV-C42	^225^YAK(GG)IVLLQNVASQR^238^
BmNPV ORF53	DNAPOL	^463^NDLSIISGQFNADKATAGISNLK^485^(GG)
BmNPV ORF15	PKIP	^150^LK(GG)LIAIK^156^
BmNPV ORF26	Ubiquitin	^43^LIFAGK(GG)QLEDSK^54^, ^55^TMADYNIQK(GG)ESTLHMVLR^72^
BGIBMGA001549-PA^†^	Ubiquitin	^1^MQIFVK(GG)TLTGK^11^, ^7^TLTGK(GG)TITLEVEASDTIENVK^27^, ^7^TLTGK(GG)TITLEVEPSDTIENVK^27^, ^12^TITLEVEASDTIENVKAK^29^(GG), ^12^TITLEVEPSDTIENVKAK^29^(GG), ^43^LIFAGK(GG)QLEDGR^54^, ^55^TLSDYNIQK(GG)ESTLHLVLR^72^

^†^The host gene BGIBMGA001549-TA encodes a pre-protein containing tandem head-to-tail repeats of the monomeric ubiquitin sequence. This pre-protein is then processed by de-ubiquitinating enzymes to produce two host ubiquitin monomers. The difference in the sequences of the two monomers is that one contains an alanine at amino acid position 19 (Ala^19^), and the other contains Pro^19^ at the same position. The Ala^19^ and Pro^19^ were underlined.

The presence of host cytoskeleton-related proteins, helicases, RNA binding and ribosomal proteins in the polyhedra matrix was not further verified due to a lack of corresponding antibodies. However, progress during the previous decade has revealed that certain viruses recruit numerous host proteins to facilitate each step of the viral replication process. Cytoskeleton-related proteins are one of the largest groups of cellular factors. The cellular actin cytoskeleton is involved in different stages of baculovirus infections^17^. This present study identified certain nucleocapsid proteins, such as P78/83, BV/ODV-C42 and VP80, to be components of the polyhedra matrix. The proteins P78/83 and BV/ODV-C42 are involved in actin polymerization and nucleocapsid trafficking^147–149^. VP80 interacts with filamentous actin^103^. Therefore, it is not surprising that actin became trapped in the polyhedra matrix. RNA-binding proteins bind to single or double stranded RNAs and play important roles in RNA splicing, export, stability, localization and translation^150^, thereby exerting significant control over numerous cellular functions. The identification of RNA binding proteins was reasonable due to the existence of certain small non-coding RNAs that could perform a structural function in polyhedra crystal formation in the BmNPV polyhedra matrix^151–153^. The present study is the first to report that certain DEAD/DEAH box helicases are components of the polyhedra matrix. The DEAD/DEAH box helicases are a family of proteins whose purpose is to unwind nucleic acids. The DEAD box helicases are involved in various aspects of RNA metabolism, including nuclear transcription, pre-mRNA splicing, ribosome biogenesis, nucleocytoplasmic transport, translation, RNA decay and organellar gene expression^154, 155^. The tomato bushy stunt virus recruits the host DEAD-box helicases Ded1p, Ded1 and RH20 to aid viral replication in infected cells, facilitate the maintenance of the full-length viral genome and suppress viral recombination, thus limiting the appearance of defective viral RNAs during replication^156–158^. Several ribosomal proteins were also identified to be incorporated into the polyhedra matrix. In addition to their roles in protein translation, some ribosomal proteins show extra-ribosomal functions, such as protein chaperone activity and the regulation of transcription^159–162^. An earlier proteomic analysis has identified the presence of the ribosomal proteins RPL18, RPL5, RPL3, and RPS6 in Ebola virions and demonstrated that reduced expression of each of these ribosomal proteins by RNA interference effectively inhibited Ebola infection in 293 T cells^163^. It would be interesting to investigate whether these identified host proteins are involved in the baculovirus life cycle.

### Identification of viral components associated with the BmNPV polyhedra matrix

Baculovirus polyhedra contain ODVs that are embedded in the crystalline polyhedra matrix. The number of ODVs that are scattered with no apparent order generally ranges from 1 to 200^164^, implying that the polyhedra harbor flexible pools for encapsulating foreign proteins, including entities such as biological nanoparticles^10^. The genome of the BmNPV T3 strain contains 136 ORFs encoding predicted proteins of over 60 amino acids^165^. In total, 91 viral proteins with one or more peptides were identified to be associated with the polyhedra matrix of BmNPV (Table 2). Certain components were observed in more than one excised gel region. Among these proteins, most have been previously reported to be envelope (E)-, envelope-and-nucleocapsid (ENC)-, and/or nucleocapsid (NC)-associated components of BV and ODV in the literature. Apparently, most viral proteins localized in the nucleus were specifically or randomly trapped in the polyhedra matrix.

**Figure 4:**
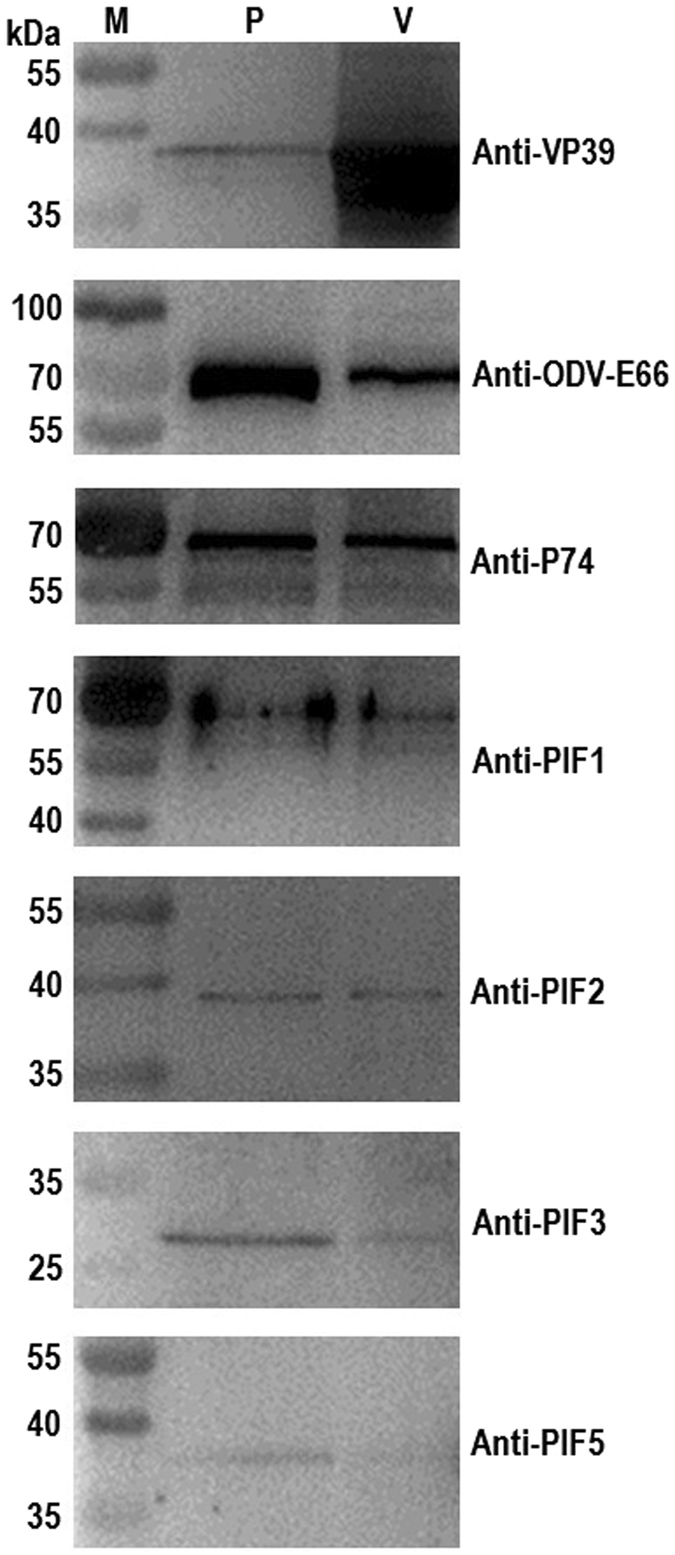
Certain proteins associated with ODV were detected in the polyhedra matrix. The polyhedra matrix (lane P) and ODV (lane V) fractions were loaded for the Western blot analyses. The antibodies used for the blotting were described previously^17^ and indicated to the right of each blot diagram. Lane M, the protein marker.

To further confirm that certain viral proteins that were associated with the ODV were encapsulated in the polyhedra matrix, Western blotting was performed. The results showed that the nucleocapsid-specific protein VP39 and certain ODV envelope-specific components, such as ODV-E66, P74, PIF1, PIF2, PIF3 and PIF5, were detected in the crystalline matrix fraction of the polyhedra (Fig. 4).

**Figure 5:**
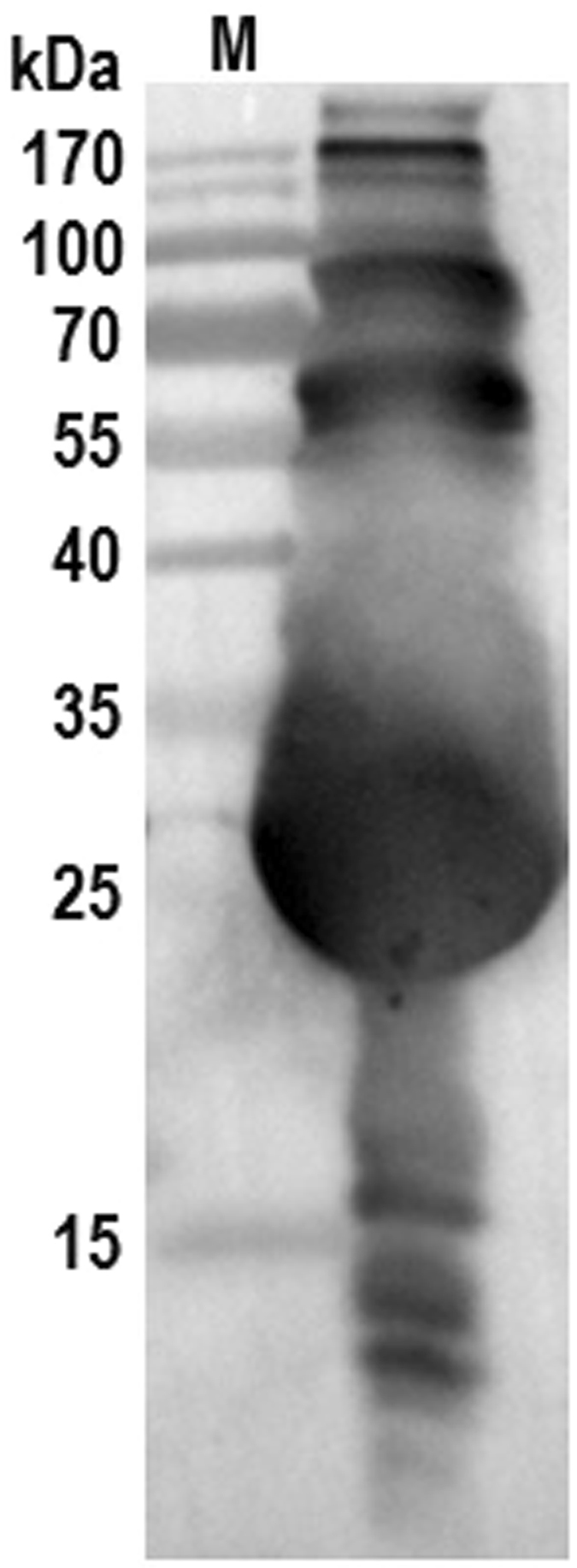
Western blot analysis of the protein polyhedrin in the polyhedra matrix. Total proteins of the polyhedra matrix fraction were subjected to SDS-PAGE and then Western blot using a mouse monoclonal anti-polyhedrin antibody^166^. Proteins were visualized using a goat anti-mouse secondary antibody conjugated to HRP and a chemiluminescence detection system. Lane M, the protein marker.

Notably, polyhedrin is a major component in the BmNPV polyhedra matrix. In this report, polyhedrin was identified in five excised gel sections by mass spectrometry. The Western blot analysis using a mouse monoclonal anti-polyhedrin antibody showed many bands with molecular weights larger and lower than the theoretical molecular size of polyhedrin of 28.8 kDa (Fig. 5). Polyhedrin is a well characterized protein. The presence of dodecameric or disulfide-linked octameric polyhedrin molecules in the polyhedra has been reported^167, 168^. Recently, polyhedrin was found to form aggregates and aggresomes in the cytoplasm of infected cells^166, 169^. Polyhedrin directly binds to *Bombyx mori* microtubule-associated protein 1-light chain 3 (BmLC3), an autophagosome marker, and is co-localized with BmLC3 to the isolation membrane of the autophagosome^166^. These findings suggest that polyhedrin may function as an autophagic adapter during the process of selective autophagy, which is regulated by post-translational modifications, such as phosphorylation and ubiquitination^170, 171^. The bands with molecular sizes larger than 28.8 kDa may represent aggregated and post-translationally modified polyhedrin, and the bands with molecular weights lower than 28.8 kDa may be partially degraded products of polyhedrin^172^. The involvement of polyhedrin in autophagy requires further clarification.

Among the 91 viral components, some proteins, such as FP25K and VLF-1, are involved in the transcription of very late genes, including *polyhedrin* ^173, 174^. Bm34 plays an important role in the transcription of *vlf-1* and *fp25K* and indirectly controls the expression of *polyhedrin* ^56^. The deletion of these genes leads to a decreased production of polyhedra^56, 57, 62, 66–68, 78^. Although these proteins are important for polyhedra production, they may not be essential for the assembly of polyhedrin into polyhedra. This finding is similar to that observed in other proteins (Table 2) as shown in the literature by studies in which polyhedra formation occurred in the nucleus upon the disruption of individual genes.

The protein DNAPOL plays an important role in viral genome replication, and when deleted, the very late phase of infection will not proceed and undoubtedly prevent the emergence of polyhedra^70^. The other two proteins, i.e., PK1 and PKIP, should also be considered. PK1, which is a serine/threonine kinase, is a component of the viral very late gene transcription initiation complex^175^ and has been shown to regulate very late *polyhedrin* promoter expression^176, 177^. Deleting *pk1* results in the failure of nucleocapsid assembly and polyhedra formation in the nucleus of infected cells^41^. PKIP is a protein that interacts with PK1 to stimulate the activity of PK1. A temperature-sensitive mutation in the *pkip* gene (amino acid A46 was mutated to T46) caused AcMNPV to lose its ability to form plaques and polyhedra at a non-permissive temperature of 33 °C upon the delayed synthesis of polyhedrin^50^. In the present study, PK1 and PKIP were identified by mass spectrometry (Table 2). Notably, further efforts are needed to better understand the roles of PK1 and PKIP in polyhedra formation.

### BM134 acts as a fusion partner to incorporate eGFP into the polyhedra matrix

The usage of polyhedra to encapsulate foreign peptide fragments was first demonstrated by McLinden and coworkers^178^, rendering polyhedra as attractive immobilized platforms. The most common approach of constructing a recombinant baculovirus with improved insecticidal properties is to engineer a donor vector that over-expresses an insect-specific toxin. The over-expression of a toxin by a recombinant baculovirus rarely contributes sufficiently to the insecticidal activity^179^. However, the incorporation of a toxin protein into polyhedra is more effective because the toxin is thus directly delivered to its normal site of activity, i.e., the gut of the host, and is expressed in the cells of the host^180^. This strategy offers an alternative to producing baculovirus-based insecticides with improved properties.

This study paid much attention to three of these 91 viral proteins due to their potential applications as carriers of engineered polyhedra. These proteins were identified by mass spectrometry with quite a fewer peptides and higher coverages, and the deletion of their individual genes leads to no or a minor impact on BV production according to the literature. One protein was polyhedrin, which is a major component of the BmNPV polyhedra matrix. Over the past decade, polyhedrin and its partial sequences were widely used to be fused, and then the fusion protein was incorporated, along with the native polyhedrin, into polyhedra^180–184^. These immobilized proteins included insecticidal toxin Cry1Ac^180^, Cry1–5^185–187^ of *Bacillus thuringiensis*, and the insect-specific cyto-insectotoxin (Cit1a) from the venom of the Central Asian spider *Lachesana tarabaevi* ^188^. The Bioassays of these recombinant viruses showed that their speed of action and pathogenicity were enhanced. The N-terminal 179 amino acids of classical swine fever virus envelope glycoprotein E2 (E2ΔC) were successfully incorporated by polyhedrin into the polyhedra, and the immobilized E2ΔC was immunogenic^182^. Additionally, the eGFP was trapped in the polyhedra and, after one month of storage, remained fluorescent^189^. These studies demonstrate that polyhedrin is a strong carrier that can incorporate foreign proteins into polyhedra.

**Figure 6:**
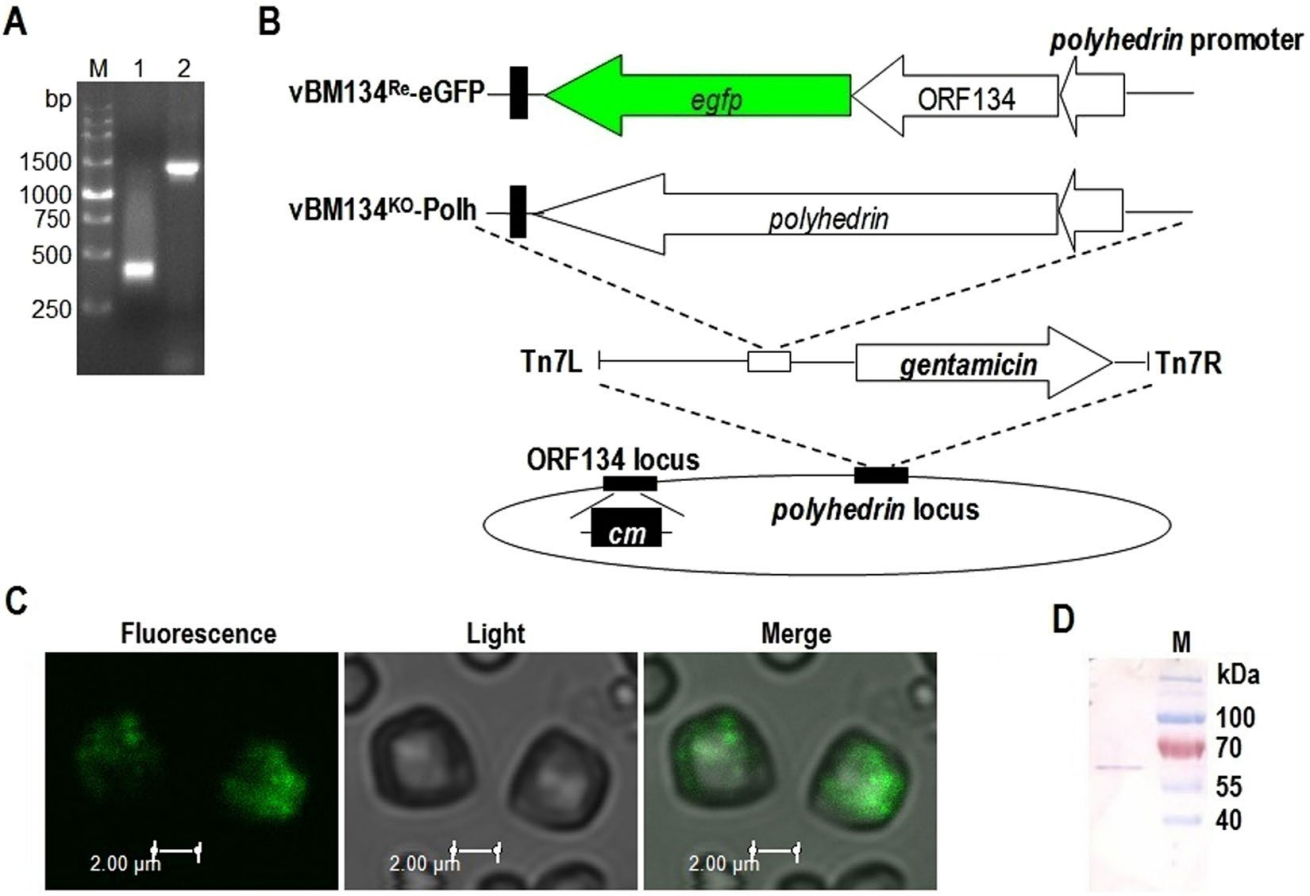
BM134 as a carrier to incorporate eGFP into the polyhedra matrix. (**A**) PCR identification of the ORF134 knockout bacmid. Lane M, DNA marker. Lane 1, DNA template of wildtype BmNPV bacmid and lane 2, template of the ORF134-disrupted bacmid, vBM134^KO^. (**B**) Construction of the BM134-eGFP-encoded nucleotide sequence- and *polyhedrin*-inserted bacmids vBM134^Re^-eGFP and vBM134^KO^-Polh. (**C**) Fluorescence of the polyhedra purified from BmN cells co-infected with viral stocks of vBM134^Re^-eGFP and vBM134^KO^-Polh. (**D**) Western blot analysis of purified polyhedra immobilized with the fusion protein BM134-eGFP using a mouse monoclonal anti-eGFP antibody. Lane M, pre-stained protein marker.

The other two proteins were ODV-E66 and BM134. ODV-E66 is an ODV envelope-specific protein^58^, and its N-terminal 23-amino-acid sequence is a hydrophobic domain that is sufficient to direct native and fusion proteins to be anchored on the viral envelope^190^. The deletion of *odv-e66* had no effect on BV production, viral DNA replication and polyhedra formation in infected Sf9 cells but had an effect on oral infectivity^59^. BM134 is encoded by the ORF134 of BmNPV, and is a 109-amino-acid protein that is associated with the ODV^144^. BM134 is likely to be nonessential because when the ORF134 was deleted, the virus appeared normal^191, 192^. The lower molecular mass of 12.4 kDa may allow BM134 to be fused and then the fusion protein is incorporated into the BmNPV polyhedra. To confirm it, an ORF134-disrupted BmNPV bacmid was successfully generated via the Red/ET homologous recombination system in *Escherichia coli* as described previously^193, 194^ (Fig. 6A). The fusion protein BM134-eGFP-encoded nucleotide sequence and *polyhedrin* fragment were inserted into the *polyhedrin* locus by a site-specific transposon^195^, to produce the bacmids vBM134^Re^-eGFP and vBM134^KO^-Polh, respectively (Fig. 6B). These bacmids were transfected into BmN cells to obtain individual BV stocks. Equal MOI (10 TCID_50_/cell) of these viral stocks were used to co-infect BmN cells, and then, at 120 h post-infection (p.i.), the polyhedra were purified for the fluorescence visualization using a Leica TCS SP5 confocal laser scanning microscope. The results showed that the green fluorescence was observed on some polyhedra particles (Fig. 6C). The immobilization of eGFP into polyhedra was further confirmed by SDS-PAGE and a Western blot analysis using a mouse monoclonal anti-eGFP antibody. Theoretically, the molecular weight of the fusion protein BM134-eGFP is 39.6 kDa. A clear band with a molecular size larger than 39.6 kDa was observed (Fig. 6D), suggesting that the protein BM134 may undergo extensively post-translational modifications. The above results showed that BM134 could be used to immobilize foreign proteins into the polyhedra, and a protein of interest could be more effectively incorporated by fusion with the C-terminus of BM134.

Notably, the polyhedrin and BM134 could be used as carriers to immobilize double proteins into the polyhedra. If the ODV-E66 or its N-terminal sequence is demonstrated in the future to be an authentic carrier, polyhedra that trap triple proteins could be engineered.

In summary, the results presented here provided novel insight into the protein composition of the baculovirus polyhedra matrix and shed light on the cellular pathways that may play a structural role. The polyhedra displayed a powerful capacity to encapsulate foreign proteins as evidenced by the immobilization of the fusion protein BM134-eGFP in the polyhedra.

## Methods

### Cells, virus, and antibodies

The *B. mori* cell line BmN was cultured as previously described^166^. The BmNPV T3 isolate^165^ was propagated in BmN cells. The virus titer was determined by end-point dilution as previously described^196^ and was expressed as TCID_50_/ml.

Antibodies, such as a rabbit polyclonal antibody to HSC/HSP70 (sc-33575, Santa Cruz Biotechnology Inc., Santa Cruz, CA, USA), mouse monoclonal anti-ubiquitinylated proteins antibody clone FK1 (04–262) and FK2 (04–263), rabbit monoclonal anti-ubiquitin Lys48-specific clone Apu2 (05–1307) and Lys63-specific clone Apu3 (05–1308) from Millipore Corporation, Billerica, MA, USA, mouse monoclonal anti-linear poly-ubiquitin clone LUB9 (AB130, LifeSensors Inc., Malvern, PA, USA), mouse monoclonal antibody to BmNPV polyhedrin from Dr. Wen-Bin Wang of Jiangsu University, China, and rabbit polyclonal antibodies to AcMNPV VP39, ODV-E66, P74, PIF1, PIF2, PIF3 and PIF5 from Dr. Zhihong Hu of State Key Laboratory of Virology, Wuhan Institute of Virology, Chinese Academy of Sciences, Wuhan, People’s Republic of China, were used.

### Polyhedra matrix protein preparation

Polyhedra were purified from infected BmN cells using a method proposed by Braunagel and Summers^197^ and then placed in a 70 °C water bath for 20 min. The protease inactivation was performed by suspending the purified polyhedra in a 10 mM HgCl_2_ solution^198^. The inhibitor HgCl_2_ was removed by washing the polyhedra with 10 mM Tris-HCl pH 7.6, 1 mM EDTA three times. ODVs were released by alkaline treatment and pelleted by continuous sucrose gradients^199^. The sample over the upper gradient was collected and added to equal volume of 2 × Laemmli buffer. The proteins were separated by 12% and 15% SDS-PAGE and stained using Coomassie brilliant blue R-250. The gel lane was excised into five contiguous sections spanning the complete gel lane based on a comparison with pre-stained molecular markers.

### In-gel digestion, mass spectrometry and protein identification

The in-gel protein digestion, LC-ESI-MS/MS analysis using a Thermo Scientific Q Exactive mass spectrometer in data-dependent mode with an automatic switch between MS and MS/MS scans, data analysis were performed as previously described^200, 201^.

### Western blot

The proteins in the polyhedra matrix and ODV fractions were separated by SDS-PAGE and transferred onto a PVDF membrane by wet electrophoresis transfer. Certain antibodies against HSC/HSP70, ubiquitin, polyhedrin, VP39, ODV-E66, P74, PIF1, PIF2, PIF3 and PIF5 were used as primary antibodies, and alkaline phosphatase or HRP-conjugated immunoglobulin G was applied as the secondary antibody. The signal was visualized using a BCIP/NBT development kit or a Tanon 5200 chemiluminescent imaging system (Tanon Science & Technology Co., Shanghai, China).

### Generation of ORF134 knockout bacmid

The BmNPV bacmid was extracted from *E. coli* BmDH10Bac^202^ and then transformed into ElectroMax^TM^ DH10B^TM^ cells (Invitrogen Life technologies, Carlsbad, CA, USA) by electroporation. The kanamycin-resistant, *lacZ*-positive colony was selected and transformed with the plasmid pRedET (Gene Bridges GmbH, Heidelberg, Germany). The colonies were isolated in the LB medium containing kanamycin and tetracycline, and designated *E. coli* BmDH10Bac-pRedET. A chloramphenicol resistance gene (*cm*) cassette with ORF134 flanking regions was amplified using the primers BM134Null-F (5′-CCAATAATATATTATGTATAGCACGTCAAAAATTAACAATGCGCGCTGAAAAGGGCGGCCGCGAAGTTCC-3′) and BM134Null-R (5′-TACGTCGCAAGCTATTTAGTTCGCGTTTTATTTGATCTCTATCATTCACTATAGGGCTCGAGGAAG-3′), and FRT-cm-FRT (Gene Bridges GmbH, Heidelberg, Germany) as the template. The underlined sequences are homologous to the upstream and downstream coding regions of ORF134. The disruption of ORF134 by *cm* was performed according to the protocol provided by the Quick & Easy Conditional Knockout Kit (Gene Bridges GmbH, Heidelberg, Germany). Colonies resistant to chloramphenicol, kanamycin and tetracycline were selected and verified by PCR using the primers BmORF134-F (5′-CGGATCCATGTATAGCACGTCAAAAAT-3′) (*Bam*HI site underlined) and BmORF134-R (5′-ACTCGAGTACGGTGCATCTGCCATATT-3′) (*Xho*I site underlined). A positive colony containing the ORF134-disrupted bacmid vBM134^KO^ was incubated at 42 °C to remove the plasmid pRedET and then chemically transformed with the helper plasmid pMON7124 to generate the *E. coli* BmDH10Bac-BM134^KO^/helper.

### Construction of the *polyhedrin*-inserted and ORF134-rescued bacmids

The fragment at locus 128,008~1,446 nt of the BmNPV T3 isolate genome was amplified by PCR using the primers Polh-F (5′-AGAATTCCAATGTACCGCGCGGCG-3′) (*Eco*RI site underlined) and Polh-R (5′-ACTGCAGACCGCCTGCACCATCG-3′) (*Pst*I site underlined) and ligated into *Eco*RI/*Pst*I sites of plasmid pFast^203^. The resulting construct pFast-Polh was transformed into *E. coli* BmDH10Bac-BM134^KO^/helper to obtain the *polyhedrin*-inserted bacmid, which was named vBM134^KO^-Polh (Fig. 6B). ORF134 without the stop codon was amplified using the primers BmORF134-F and BmORF134-R and then inserted into the *polyhedrin*-removed pP_ph_-Polyhedrin-eGFP^166^ to obtain pP_ph_-ORF134-eGFP. This donor plasmid was transformed into *E. coli* BmDH10Bac-BM134^KO^/helper competent cells to produce the ORF134-repaired bacmid designated vBM134^Re^-eGFP (Fig. 6B). Positive transpositions were verified by PCR using the pUC/M13 forward and reverse primers.

### Incorporation of eGFP into the polyhedra

Bacmids vBM134^KO^-Polh and vBM134^Re^-eGFP were transfected into BmN cells to obtain individual BV stocks. Equal MOI (10 TCID_50_/cell) of P2 viral stocks were used to co-infect BmN cells. At 120 h p.i., the polyhedra were purified for the fluorescence visualization under a Leica TCS SP5 confocal laser scanning microscope and a Western blot analysis using a mouse monoclonal anti-eGFP antibody.

## Electronic supplementary material

Supplementary Information
