# Supplementary material for: Protein composition analysis of polyhedra matrix of Bombyx mori nucleopolyhedrovirus (BmNPV) showed powerful capacity of polyhedra to encapsulate foreign proteins
Source: Sci Rep. 2017 Aug 18;7:8768. doi: 10.1038/s41598-017-08987-8 (PMC5562830; doi:10.1038/s41598-017-08987-8)
Supplement: Supplementary file 1 — Supplementary Information [file 41598_2017_8987_MOESM1_ESM.doc]

**Protein composition analysis of polyhedra matrix of *Bombyx mori* nucleopolyhedrovirus (BmNPV) showed powerful capacity of polyhedra to encapsulate foreign proteins**

Zhong-Jian Guo^1^*, Meng-Han Yu^1^, Xian-Yun Dong^1^, Wei-Li Wang^2^, Ting Tian^1^, Xian-Yin Yu^1^ & Xu-Dong Tang^3^*

^1^ Institute of Life Sciences, Jiangsu University, 301# Xuefu Road, Zhenjiang 212013, Jiangsu, P.R. China

^2^ Department of Occupation Evaluation, Hebei Ankang Occupation Health Technology Service Co. Ltd., 286# Tianshan Street, Shijiazhuang 050000, Hebei, P.R. China

^3^ College of Biotechnology, Jiangsu University of Science and technology, 2# Mengxi Road, Zhenjiang 212018, Jiangsu, P.R. China

Correspondence and requests for materials should be addressed to Z-J. G. (email: [gzh762677@ujs.edu.cn](mailto:gzh762677@ujs.edu.cn)) or X-D. T. (email: [xudongt@aliyun.com](mailto:xudongt@aliyun.com))

Supplementary Tables

**Supplementary Table S1. Viral Proteins identified in the M1 section of the gel.**

| ORF | Protein | Size(amino acids) | Sequence | Sequence(s) of corresponding peptide(s) |
| --- | --- | --- | --- | --- |
| 1 | polyhedrin | 245 | MPNYSYTPTIGRTYVYDNKYYKNLGCLIKNAKRKKHLVEHEQEEKQWDLLDNYMVAEDPFLGPGKNQKLTLFKEIRSVKPDTMKLIVNWSGKEFLRETWTRFVEDSFPIVNDQEVMDVYLVANLKPTRPNRCYKFLAQHALRWEEDYVPHEVIRIVEPSYVGMNNEYRISLAKKGGGCPIMNIHSEYTNSFESFVNRVIWENFYKPIVYIGTDSAEEEEILIEVSLVFKIKEFAPDAPLFTGPAY | ^1^MPNYSYTPTIGR^12^, ^13^TYVYDNKYYK^22^, ^36^HLVEHEQEEK^45^, ^46^QWDLLDNYMVAEDPFLGPGK^65^, ^77^SVKPDTMKLIVNWSGK^92^,^85^LIVNWSGK^92^, ^85^LIVNWSGKEFLR^96^, ^93^EFLRETWTR^101^, ^132^CYKFLAQHALR^142^, ^135^FLAQHALR^142^, ^135^FLAQHALRWEEDYVPHEVIR^154^, ^143^WEEDYVPHEVIR^154^, ^155^IVEPSYVGMNNEYRISLAK^173^, ^155^IVEPSYVGMNNEYR^168^, ^174^KGGGCPIMNIHSEYTNSFESFVNR^197^, ^175^GGGCPIMNIHSEYTNSFESFVNR^197^, ^230^IKEFAPDAPLFTGPAY^245^, ^232^EFAPDAPLFTGPAY^245^ |
| 66 | GP41 | 403 | MTDERGNFYYNTPPPPLRYPSNPATAIFTNAQTYNNAPGYVPPTTRDNKMDTSRSNSTNSVAIAPYNKSKEPTLDAGESIWYNKCVDFVQKIIRYYRCNDMSELSPLMIHFINTIRDMCIDTNPINVNVVKRFESEETMIRHLIRLQKELGQGNAAESLPSDSNIFQASFVLNSLPAYAQKFYNGGADMLGKDALAEAAKQLSLAVQYMVAESVTCNIPIPLPFNQQLANNYMTLLLKHATLPPNIQSAVESRRFPHINMINDLINAVIDDLFAGGGDYYHYVLNEKNRARIMSLKENVAFLAPLSASANIFNYMAELATRAGKQPSMFQNATFLTSAANAVNSPAAHLTKNACQDSLTELAFQNETLRRFIFQQINYNKDANAIIAAAAPNVTRPNTKGRTV | ^6^GNFYYNTPPPPLR^18^, ^55^SNSTNSVAIAPYNK^68^, ^69^SKEPTLDAGESIWYNK^84^, ^71^EPTLDAGESIWYNK^84^, ^117^DMCIDTNPINVNVVK^131^, ^132^RFESEETMIR^141^, ^182^FYNGGADMLGK^192^, ^239^HATLPPNIQSAVESR^253^, ^325^QPSMFQNATFLTSAANAVNSPAAHLTK^351^, ^352^NACQDSLTELAFQNETLR^369^, ^371^FIFQQINYNK^380^, ^381^DANAIIAAAAPNVTRPNTK^399^ |
| 88 | VP80 | 692 | MNDSNSLLITRLAAQILSRNIQTVDVIVDDKTLSLEEKIDTLTSMVLAVNGLPQSPPRVSSSDLAASSIKNNSKLVGNDFEMRYNVLRMAVVFVKRYPKYYNETTAGLVAEIENNLLQYQNYVNQGNYQNIQGYDSLLNKAEECYVKIDRLFKESIKKIMDDTEAFEKEQEAERLRAEQTAANALLNRRAQTSADDVVNRADANISTAFSDPLPGSSAPRYMYESSESDTYMEPARHTAEHYTDQDKDYNAAYTADEYNSLVRTVLLRLIEKALATLTNRLHITTIDQLKKFRDYLNSDADAGEFQIFLNQEDCVMLKNLSNLASKFFNVRCVADTLEVMLEALRNNIELVQPESDAVRRIVIKMTQEIKDSTTPLYNIAMYKSDYDAIKNKNIKTLFDLYNDRLPINFLDTSATSPAVRKTPGKRSAEDDLLPTRSNKRANRPEISVISSEDEQEDDDVEDVDYEKESKRRKLEDEDFLKLKALEFSKDIVNEKLQKIIVVTDGMKRLYEYCNCKNSLETLPSAANYGSLLKRLNLYNLDHIEMNVNFYELLFPLTLYNDNDNSDKTLSHQLVNYIFLASNYFQNCAKNFNYMRETFNVFGPFKQIDFMVMFVIKFNFLCDMRNFAKLIDELVPNKQPNMRIHSVLVMRDKIIKLAFSNLQFQTFSKKDKTRNTKHLQRLIMLMNANYNVI | ^201^ADANISTAFSDPLPGSSAPR^220^, ^221^YMYESSESDTYMEPAR^236^, ^237^HTAEHYTDQDKDYNAAYTADEYNSLVR^263^, ^346^NNIELVQPESDAVR^359^, ^371^DSTTPLYNIAMYK^383^, ^396^TLFDLYNDR^404^, ^405^LPINFLDTSATSPAVR^420^, ^617^FNFLCDMR^624^, ^625^NFAKLIDELVPNK^637^ |
| 54 |  | 805 | MQRWPKYGGTDVNTRTVHDLLNTINTMSARIKTLERYEHALREIHKVVVILKPSANTHGFEPDALPALIMQFLSDFAGRDINTLTHNINYKYDYNYPQPPAPVPAMQPPPPPPFHQPPAPPQPPYYNNYPYYPPYPFSTQPPESNVAGVGGSQSLNQITLTNEEESELTALLKNMQTNMTWELVQNFVEVLIRIVRVHVVNNVTMINVISSITSVRTLIDYNFTEFIRCVYQKTNIRFAIDQYLCTNIVTFIDFFTRVFYLVTRTNFHFTTFDQLTQYSDELYTKIQTSILNSAAPLSPPTVETVNSDVVISNLQEQLKRERALMQQIVEQHRIANERVETMQSQYDELDLKYKEVFEDKSEFAQQKSENVQKIKQLERSNRELNGTVQKLRDENAEILSEIQLQKGDLDEYKNTNRQLNEDIHRLKKRMESTFDKNYVKTLNDKIESLEKQLDDTQNLNRELESSINETAQTYKFDAKDVMELKQSVSIKDQEIAMKNAQYLELSATYQQTVNELAATKNELSQIATTNQSLFAENEESKVLLEGALAFIDSFYQIIMQIEKPDYVPISKPQLTAQESIYQTDYIKDWLQKLRSKLSNADVANLQSVSEVNDLKTQIISIVPRNIVNRILKENYKVKVENVNAELMENVAVISAVSALVQQYEQSEKQNIRLRKDFEIKLNELQRLLDQNQTDFESISEFISRDPAFNRNLNDERFQNLKQQCDEMSSKYSALKTTKIKEMESIADQAVKSEMSKLNTQLDELNSLFVKYNRKAQDIFEWKTSMLKRYETLARTTAASVQPNVE | ^16^TVHDLLNTINTMSAR^30^, ^339^VETMQSQYDELDLK^352^, ^391^LRDENAEILSEIQLQK^406^, ^462^ELESSINETAQTYK^475^, ^521^NELSQIATTNQSLFAENEESK^541^, ^597^LSNADVANLQSVSEVNDLK^615^, ^616^TQIISIVPR^624^, ^757^LNTQLDELNSLFVK^770^ |
| 37 | ODV-E66 | 702 | MSTVLIIVVVVIFLICFWCLLNSSNNSNNKNDANRNNVFVDFDPLPFDAFDINTFDITNDDSIVAFQQNNIQELENFEQWFKNNLSYSFSQKAEKVVNPNRNWNDNTVFDNLSPWTSVPDFGTVCHTLIGYCVRYNNTSDALYQNPELAYNLINGLRIICSKLPDPPPHQQAPWGPVADWYHFTITMPEVFMNITIVLNETQHYDEAAALTRYWLGLYLPTAVNSMGWHRTAGNSMRMGVPYTYSQMLRGYLLAEIRQEQGIQEILNTIAFPYVTRGNGLHVDSIYIDHIDVRAYGYLINSYFTFAYYTYYFGDEVINTVGLTRAIENVGSPEGVVVPGVMSRNGTLYSNVIGNFITYPLAVHSADYSKVLTKLSKTYYGSVVGVTDRLAYYESDPTNNVQAPLWTMARRIWNRRGRIINYNANTVPFESGVILQSLNGIMRIPSGTTSTQSFRPAIGQTAIAKTDTAGAILVYAKFAEMNNLQFKSCTLFYDHGMFQLYYNIGVEPNSLNNTNGRVIVLSRDTSVNTNDLSFEAQRINNNNSSEGTTFNGVVCHRVPITNINVPSLTVRSPNSSVELVEQIISFQSMYTATASACYKLNVEGHSDSLRAFRVDSDENIYVNVGNGVKALFNYPWVMVKENNMVSFMSANEDTTIQFSVIMNSFTSIGEPALKYSPSNCFAYGNGFKLNDSTFDLQFIFEIV | ^238^MGVPYTYSQMLR^249^, ^325^AIENVGSPEGVVVPGVMSR^343^, ^377^TYYGSVVGVTDR^388^, ^389^LAYYESDPTNNVQAPLWTMAR^409^, ^443^IPSGTTSTQSFRPAIGQTAIAK^464^, ^465^TDTAGAILVYAK^476^, ^477^FAEMNNLQFK^486^, ^523^DTSVNTNDLSFEAQR^537^, ^557^VPITNINVPSLTVR^570^, ^629^ALFNYPWVMVK^639^ |
| 134 |  | 109 | MYSTSKINNARVVASQHDYDRDQIKRELNSLRRNVHDMCTRSGTSFDCNKFLRSDDMTPVVTTITPKRTADYKITEYVSDVKTIKPSNRPLVESGPLVQEAAKYGRCTV | ^54^SDDMTPVVTTITPK^67^, ^74^ITEYVSDVK^82^, ^83^TIKPSNRPLVESGPLVQEAAK^103^ |
| 75 |  | 259 | MIPLTPLFSRYKDSYLLYSFRLIDLLRASKSAHLTKLLSSQATYLYHFACLMKYKDIQKYEVQQLIEWAINASPDMDLQQFRIEFMDKTTELNLRSCQPKSFTYTFTTIWDTMHFLSLIIDDMVYTRDKSSLDFVMQQLKTMKVLFYNVFFILQCAMCRDHYMNVKGFLIYHIELIEIALDKEKYGTDITFVDSYQQETAGADAAAVSNNMLMKNLMAYVIMTFHNHVNDYKWIQRNQKPPAHYERMTWGEYKKLLNLQ | ^1^MIPLTPLFSR^10^, ^11^YKDSYLLYSFR^21^, ^13^DSYLLYSFR^21^, ^22^LIDLLR^27^, ^37^LLSSQATYLYHFACLMK^53^, ^128^DKSSLDFVMQQLK^140^, ^130^SSLDFVMQQLK^140^ |
| 118 |  | 476 | MSGGGNLLTLERDHFKYLFLTSYFDLKDNEHVPSEPMAFIRNYLNCTFDLLDDAVLMNYFNYLQSMQLKHLVGSTSTNIFKFVKPQFRFVCNRTTVDILEFDTRMYIKPGTHVYATNLFTSNPRKMMAFLYAEFGKVFKNKIFVNINNYGCVLAGSAGFLFDDAYVDWNGVRMCAAPRLDNNMHPFRLYLLGEDMAKHFVDNNILPPHPSNAKTRKINNSMFMLKNFYKGLPLFKLKYTVVNSTKIVTRKPNDIFNEIDKELNGNCPFIKFIQRDYIFDAQFPPDLLDLLNEYMTKSSIMKIITKFVIEENPAMNGEMSREIILDRYSVDNYRKLYIKMEITNQFPVMYDHESSYIFVSKDILQLKGTMNAFYAPKQRILSILAINRLFGATETIDFHPNLLVYRQSSPPVRLTGDVYVVDKNEKVFLVKHVFSNTVPAYLLIRGDYESSSELKSLRDLNPWVQNTLLKLLIPDSV | ^28^DNEHVPSEPMAFIR^41^, ^94^TTVDILEFDTR^104^, ^188^LYLLGEDMAK^197^, ^198^HFVDNNILPPHPSNAK^213^, ^306^FVIEENPAMNGEMSR^320^, ^379^ILSILAINR^387^, ^431^HVFSNTVPAYLLIR^444^, ^458^DLNPWVQNTLLK^469^ |
| 26 | Ubiquitin | 77 | MQIFIKTLTGKTITAETEPAETVADLKQKIADKEGVPVDQQRLIFAGKQLEDSKTMADYNIQKESTLHMVLRLRGGY | ^12^TITAETEPAETVADLK^27^, ^64^ESTLHMVLR^72^ |
| 106 | P24 | 195 | MKTDAQSTSNTHNFMYSPDNNLEVVIITNSDGDHDGYLELTAAAKIMSPFISNGGSTVWTNAAPSHKLIKNNKNYIHVFGLFKYLSNYNLNNKKRPKEYYVLKSIISDLLMGAQGKVFDPLCEVKTQLCAIQESLNEAISTLNVHAAANSPAPDINKLQDMIQDLQSEYNKKITFTTDTILENLKNIKDLMCLNK | ^46^IMSPFISNGGSTVWTNAAPSHK^67^, ^74^NYIHVFGLFK^83^, ^104^SIISDLLMGAQGK^116^, ^158^LQDMIQDLQSEYNK^171^, ^158^LQDMIQDLQSEYNKK^172^, ^173^ITFTTDTILENLK^185^ |
| 63 | VLF-1 | 379 | MNGFNVRNENNFNSWKIKIQSTPRFESVFDLATDRQRCTPDEVKNNSLWSKYMFPKPFAPTTLKSYKSRFIKIVYCSVDDVHLEDMSYSLDMEFDSIENQTLLIDPQELCRRMLELRSVTKETLQLTINFYTNMMNLPEYKIPRMVMLPRDKELKNIRKKEKNLMLKNVIDTILNFINDKIKMLNGDYVHDRGLIRGAIVFCIMLGTGMRINEARQLSVNDLNVLIKKGKLHSDTINLKRKRSRNNTLNNIKMKPLELAREIYSRNPTILQISKNTSTPFKDFRRLLEESGVEMERPRSNMIRHYLSSNLYNSGVPLQKVAKLMNHESSASTKHYLNKYNIGLDETSSEEENNNNDDDAQHNRNSSGSSGESLLYYRNE | ^25^FESVFDLATDR^35^, ^52^YMFPKPFAPTTLK^64^, ^216^QLSVNDLNVLIK^227^, ^216^QLSVNDLNVLIKK^228^, ^286^LLEESGVEMERPR^298^, ^304^HYLSSNLYNSGVPLQK^319^ |
| 108 | PP34 | 315 | MKPTNNVMFDDASVLWIDTDYIYQNLKMPLQAFQQLLFTIPSKHRKMINDAGGSCHNTVKYMVDIYGASVLILRTPCSFADQLLSTFIANNYLCYFYRRRRSRSRSRSRSPHGRPRSRSRSRSRSRSRSSSPRQGRRQIFDALEKIRHQNDMLMNNVNQINLNQTNQFLELSNVMTGVRNQNVQLLAALETAKDVILTRLNTLLSEITDSLPDLTLMLDKLAEQLLEAINTMQQTQRNELNNTNSILTNLASSITNINGTLNNLLTAIENLAGGGGGGGGGGGGNFNEADRQKLDLVHTLVNDIKNILTGTLTTK | ^28^MPLQAFQQLLFTIPSK^43^, ^138^QIFDALEK^145^, ^180^NQNVQLLAALETAK^193^, ^292^QKLDLVHTLVNDIK^305^, ^294^LDLVHTLVNDIK^305^ |
| 92 |  | 391 | MECPFQIQVCISDRFFAFPHNLVEPQSDVGNKLIENLIVYVPTDDDRLYIDKKQFPKFNSVLVYRHEHDVNIDSRSPKKTASATIVYWNPLMPITEIGAGETRVFSVLLTNNLFYCNTMIIQHENPKCPIEFTYPETDMQSACSALLKNRNGQSVPPPIKSNLRPIACEIPLSHFKELVESNDFLLCFNLETSTMVKILSLKRIFCIFQYRKQPARYVINLPHEEIDNLYNKLNWERTRRLMKGDVPSNCATVNRSSLKYIKQAQSLLGIPDYSQTVVDFVKMFQKIIFPYQLVPNVIIKLNNFDQIMSVAPNKAESYKKIRLFCKNDSIAISSNGIVPINMPDFSPSNNAFDYSDYVNRTNINFVTQRVLIDGGFSSGVTVTPIKYNYYL | ^15^FFAFPHNLVEPQSDVGNK^32^, ^161^SNLRPIACEIPLSHFK^176^, ^204^IFCIFQYR^211^, ^217^YVINLPHEEIDNLYNK^232^, ^301^LNNFDQIMSVAPNK^314^ |
| 77 | ODV-E25 | 228 | MWKIVLLIVLLVLIYLYWTNALNLNSLTEASPSLGQSSESVESDENKRLNVKLNNARVANLRIAHGDNKLSQMYIAEKPLSIDDIVKEGSNKVGTNSIFLGTVYDYGVKSPNAASTSSNVTMTRGTANFDIKEFKSMFIVFKGITPTKTVEDNGMLRFEVDSMIVCLIDPNTGPLSEREVRELRKSNCTLVYTKNEAAQQVLLENNFTAINADQTAYLKNYKSYREMN | ^70^LSQMYIAEKPLSIDDIVK^87^, ^93^VGTNSIFLGTVYDYGVK^109^ |
| 94 |  | 424 | MRKYALLQKMIINELLFLNDNVNYATNKLFSKDQANGELQKLSAMLLNYKKSNKNVPNIKFDLKNLSFMLENTDKIDIIQFDDVKNYVQPAIVNLFESHNRSLNNYSTELNTLLENGNENLVPNITDIDNIKLSHMQLARLLCYTAVVESRNSKPWKAIFNNDTCVLTDSFFNYIMNILNMIKTNQGSLAHNLSVVYHIENIQMNLQNKLKPRSITIEIVDKDKFENKHSDIEVCYVMNNRLHPQDVNSQQTLMCSSFVELNALPFCLYNETLPDDQSMSVFNLYKFEQTKNNSVSKPSRLGNVMFVNSLIDKPITRETIVDIINSYHNACQNLKRSGHRVVGDYRAYERDYKLAALDFIILMLVTSITHRTLKYNMLNIHEKMFQELKTIVCKHSAAKLYDILINYDINKEPLNNFRYNYEVL | ^76^IDIIQFDDVK^85^, ^86^NYVQPAIVNLFESHNR^101^, ^301^LGNVMFVNSLIDKPITR^317^ |
| 87 | P45 | 387 | MCAYRLQYSLRFNTYDRFENVCFEAQLLRDEIDSLCFLFSKYFNQSLIVDGKGLTFFTEFNKCIVSIKSSFENQANNTDNIHNVKNIFSIFLRDEFIKQVPQFRTIMQYLQTYYNPTPAPDVDEIMCQSCKPANKIQCFECKCRYLASSLSTLDEGLQNGWDIFLRPMFGMPLMLYVLLRTDYKNESDVINENNLITQIFVQFFYNLICDKAYSLYTKRDMCVPFVKECKKATIGLRQEDHERVLSILNAQCNGFSTAANGNRLLLPFKNFMIKMGRNTNMKKVNKIASTVLIGFYLRHYLESLPNKAYPVAELELRNVCRFIMSKYSDENINLLIHKLKLIKIDICNVLMTEMIVPESFIRHIITKYQLDNEISLLIELNHDCFNK | ^42^YFNQSLIVDGK^52^, ^53^GLTFFTEFNK^62^, ^264^LLLPFK^269^, ^308^AYPVAELELR^317^, ^327^YSDENINLLIHK^338^ |
| 69 | P95 | 839 | MMSGVMLLVFAIFLIIAFTLIYLAIYFKFDETTYTKRLQVMIEYIKRTNADEPTPNVIGYVSDITQNTYTVTWFNTVDLSTYQESVHDDRNEIFDFLNQKLQPVDRIVHDRVRANDENPNEFILSGDKDDVTMKCPAYFNFDYAQLKCVPVPPCDNKPAGRYPMDERLLDTLVLNQHLDKDYSSNEHLYHPTFYLRCFANGAHAVEECPDNYTFDAKTRQCKVNELCENRPDGYILSYFPSNLLVNQFMQCVSGRHVVRECPANKIFDRNLMSCVEAHPCTFNGAGHTYITADISDAQYFKCLNNNESQLMTCINRIRNSDNQYECSGDSRCIDLPNGTGQQVFKHADDDISYNSGQLVCDNFEIISNIECDQSNVFENKLFMDKFRLNMQFPTEVFDGTACVPATADNVNFLRSTFAIENIPNHYDIDMQTSMLGKIEMIKQLVSKDLSLNNDAIFAQWLLYARDKNAIGLNPLTGEPIDCFGNNLYDVFDARRANVCKDLGKSVLKTLNFGDGEFLNVLSDTLTGKDEDYRQFCAISYENGQKIVENEHFQRRILTNILQSDVCANIYTTLYQKYTTLNPKYTTTPLQYNRILVKRPKNIEIYGANTRLKNATIPKNATTISPVFNPFENQPNNRQNDSISPLFNPFQTTDAVWYSEPDGDEAVAPPPTAPPPPSEPEPEPEPELPSPLILDNKDLFYSCHYSVPFFKLTSCHAENDVIINALNELRNNVKVDADCESAKDLSHVLNAYAYVGNGIGCRSAYDGDAIVVKKEAVPSHVYANLNTQSNDGVKYNRWLHVKNDQYMACPEELYDNDEFKCNVESDKLYYLDNLQEDSIV | ^619^NATTISPVFNPFENQPNNR^637^, ^711^LTSCHAENDVIINALNELR^729^ |
| 17 |  | 129 | MDGSVVSMCVDNAFAYTTDDLLKNISFSHSKCAPFKLQNYTVLKRLSNGFIDKSVDVGSISELQKFNFKINRLTSYISNIFEYEFVVLEHNLSTVHVINAETKTKLGHINVSLNQNDPNVLILTVTLTS | ^54^SVDVGSISELQK^65^ |
| 72 | VP39 | 350 | MALMPVGMAPRQMRVNRCIFASIVSFDACITYKSPCSPDAYHDDGWFICNSHLIKRFKMSKMVLPIFDEDDNQFKMTIARHLVGNKERGIKRILIPSATNYQEVFNLNSMMQAEQLIFHLIYNNEEAVNVICDNLKYTEGFTSGTQRVIHSVYATTRSILDTTNPNTFCSRVSRDELRFFDVTNARTHRGGVGDQLFNNYSGFLQNLIRRAVAPEYLQIDTEELRFRNSATCIIDETGLVASVPDGPELYNPIRSSDIMKSQPNRLQIRNVLKFEGDTRELDRTLSGYEEYPTYVPLFLGYQIINSENNFLRNDFISRANPNATLGGGVGALAGPAPGVVLGEAGGGVAA | ^62^MVLPIFDEDDNQFK^75^ |
| 27 | 39K | 277 | MVNMPEQQSSTETAAVCKNEKLLNKLESSSYNKSNMDQLIAIVNFLEKKNINYILNVMPVMQDERKMSKRKKKVINNNKYILFNSWYTKIKQPEWPSSPAMWDLVKNTPELADFVFIFDHTEKMGKKMADRSTSSSSSDNAAIPASKKRQTAVLTNANLAELKESCEMRDKLYSEFYSLLNETFNNNVAPLLSSIYDEVLTRDFITKNMAKFKTVALKLPVAPSTTEYVPTSISGSKKRKNSVPAKQRSSIKNRRNTTAAPTLLMVSDNTQDTNMSE | ^50^NINYILNVMPVMQDER^65^ |
| 48 |  | 83 | MLPKYYQNLPYNGKRIFEKFYDRSLQKYKSTHIATKLACCAVRKKYILIDCKWQPRPDANNSDTTTTNTEDSTTDTETEDEIA | ^5^YYQNLPYNGK^14^ |
| 23 | SOD | 151 | MKAICIISGDVHGKIYFQQESANRPLKISGYLLNLPRGLHGFHVHEYGDTSNGCTSAGEHFNPTNEDHGAPDAEIRHVGDLGNIKSVGYNSLTEINMMDNVMSLYGPHNIIGRSLVVHTDKDDLGLTEHPLSKTTGNSDGRLGCGIIAICK | ^28^ISGYLLNLPR^37^, ^114^SLVVHTDKDDLGLTEHPLSK^133^ |
| 70 | VP15 | 126 | MNTRGCVNNNKMIFMNALGLQPQSKVKIIAHKTLEKFKRDAYTRFKGVKAIKNELKTYNLTLQQYNEALNQCALNDSRWRDTNNWHHDIKEGVKINKRHIYRVNFNSKTKEIKEYYYIKVECYVNN | ^57^TYNLTLQQYNEALNQCALNDSR^78^ |
| 29 |  | 217 | MRNAAGLFMIIEPDKAVLLCARRAYRSANAPAADINDTFLEKISIPRGHRDCCDAKVYETAVREFVEETGRFFDSAFIYKFPFTLQWKDDGVTYKYLIYVGVVRGNLIDVNAKPNTYTVKLLPGTFGNDYRIMLKPRRFNCEITRSLAIVPLNKYFNYMNDKQLITYDYSNYIEFFSFVRSIKKRFDNRQLQDFFYATLKKIDNNDAPQKLHALRRV | ^16^AVLLCARR^23^ |
| 62 |  | 85 | MNLYLLLGALTIFSLVYDKKENSIFLYLLILFLVFIIVVSPAIISKNTESNVEDIPSHKAKSVRKKLEIEQALDAILNKNTSSID | ^67^LEIEQALDAILNK^79^ |
| 10 |  | 356 | MERLLNQLNLGVLPYITTKDIEDRLRDKIVAKAKLAFIKDCFEAVVCENGGLFVLTGGAAVTCHIDDDDYKSALKCIDFDYYGLCSKKMFCNLQTNLQKCVDQHYAELDVLTRQIYMSNPLVMLKCYQNGAYRLNGQIDLHLNRHIKCIKTQYNDEFDLVRFALQIDITSAYGVDEYTDNCVKITTAPLSFNVFFVNVRIMKRPFNADRCIKNFSLLGNEYHVLVSSLQRVLNDQLMCLLKDIFTNKFDYKIKRRLNHLKRLFANLPAESYNSCVNDLTDMYLYKEQNETITNFVKKILDISGPDLGCRKLMRIYLNTDTFRGQLPAYLTHYVNYPHKNLCDQNWKRFMSCIFSLY | ^213^NFSLLGNEYHVLVSSLQR^230^ |
| 93 |  | 67 | MDNYSLHNFYNNNRKPLKPTTLHDGNIKKSVYENITYVRKLMCKENMPGEHDHKFYNRGYNKENKYK | ^1^MDNYSLHNFYNNNR^14^ |
| 84 | P6.9 | 65 | MVYRRRRRSSTGATYGLTRRRRSSAGITRRRRSSGYRRRPGRPRTYRRSRSRSLTSRRSYRTRYY | ^23^SSAGITRR^30^ |
| 53 | Polymerase | 986 | MKIYSYNELKTRFAEYAKPGEFSITSADTFRIIRLHYDEKQGCLFAFCNTNIEKRVLQFYFKVKLNLYSYKQCYDKHIFPSCRNKCISYTTFVAPGVEGNCLNKINVIKYERNKAAPSDNAACLDKFLHNVNRVHMQTPFVEGAYMRFKKTQRCQNNYVGGSTTRMFNLQHFYEDFELVDEMTLTSGIMPVLSCYDIETHSDGHNMSKASVDCIMSIGFVVYKNDEYARFCFMYHKLPTEIPETHDDDTHVVMFQNEVDMITAFFDMIKITNPDVILDFNGDVFDLPYILGRLNKTKMLLKRYDLPAAAPTTKLFINKLGNKVDTYYFNYYIHIDLYKFFSSDSNQHKVENFQLNTISSYYLGENKIDLPWTEMVKMYNTRRLDVIAKYNVQDCMLPIKLFVKLKMADSVYSQCILHRLCTDDVICNISHLISVACFYAAITNTRINESTGKEEPDPYFFNKNDLSIISGQFNADKATAGISNLKRKLTPLKNIPKDAINLGPANQTVKYKGGKVLQPRASIYKNAFSLDFNSLYLTIMIAICACLSNLVLCEDGNVYLNHNSRAIVVKLLLKLLSERCKFKKNRDNQSESAFLYDLYDQKQNSVKRTANSIYGYYGIFYKVLANYITRVGRNQLRRAISLIEGLSNDPEILKKFNLNSIGFKVVYGDTDSTFVLPTFNYNEIFDETDTLKQICTHVETRVNSSFTDGYKMAFENLMKVLIILKKKKYCYLNSENKIVYKGWLVKKDMPVFMRIAFRTAVEQILRHLDINKCLQSLQASFYEYYDEFAKSKPMTDYSFSMTYNDNPGKKRKSADDNNEGPSPKRRVITVARHCREILVNKGTDFVPGNGDRIPYLLIDIEGKVTEKAYPLRLFDPVKMRISWIKHMGILCTFMNELLEIFGDEQKDNLAKCFTAIMQKYMQNQLYDRKEPVLVKINQKKCSVKRKRDDDDDNNDDDDDDGCDSSDSENDTQCANNTYKFCLYKIKK | ^477^ATAGISNLK^485^, ^493^NIPKDAINLGPANQTVK^509^ |
| 116 | ME53 | 451 | MNRFFRENNIFDAPKTGGKGRVKSLPTPVANSPLSPVRQPPKSNIKPPTRISLPTRTFSANPLERSISSSIVSKKPVVNRKDGYFVPPEFGNKLESLPAYSDKLDFKQERDLRMHFMSDLERNIMKATLKFSTNYIMGYINSKDMRMTGKFASSSVKYKKTTEQHMSDSRCTTCNYRFKDNTRAWFLYVVVHIEKPLDDSDRIDICCQKCYLYHNVPKTSYEIYPSINLVDLSYLARERFFYQYIFPVSLEHTTEVKELKIDDHNCKVFEIIRRIIRNHKEPNERIQTIDLSTTGGLVLRETYTNIVLQRYRSMCTRPDAADDVNCFILQEPSEMMAALQDNRFSGIKGTVFATVKVKKFTEVLDGGAITFPSRPTINNYCKLCKKTKLYYKNPVLYCTKCGFTNVYHFPEYSKLVYYSKAIKNFEMHNEMIIYYDLKMYKKLINIVNNNV | ^443^LINIVNNNV^451^ |
| 71 | CG30 | 267 | MEFVKLQCNICFSVAEIKNYFMQPIDRLTIIPVLELDTCKHQLCSMCIRKIRKRKKIPCPLCRVESLHFNVYSINRNVVDVIKCSVTSVAQWNKINDNFDAASLASVLFEKSLLDDAEDSNNAANSDDTMLSESQAILKKLQIDIAEQTQLNIKRQLDLNKLQQTSVFMQEKLDRIKNDYNNMHKSFKELQLKRISTEKALKSLNDDYAKLAAKNARLSNENKVLSNKNIELIKHKNLLQNEYTTLQSYKCITNATTITTNVTINVD | ^141^LQIDIAEQTQLNIK^154^ |
| 95a |  | 56 | MYFTSRFLSALGTSNTLAVRCIMLKINSADAELYRPRFIFCATRHFVRHTTLFTLN | ^7^FLSALGTSNTLAVR^20^ |
| 14 |  | 673 | MDGVKLLGTCALIILLSTTNTVVGRDRITFTPIEDSAGLVFERMYGLRHHTDERFVFVKKFNFASVLQELNNIKSKIELYEAQVSTCKNVRQIKQNRSSNIKTRIEKQLQFLTPLNKNFITYSVEDSISSNEVLDNIDLEYDDGVDFDVYDEYEPSSHWSNMTVSDAKALLQNPPKDRVMFLDTVTTNNVSSKYEEYIDCIVSNRTVENECMFLANMMNVLNDKLDDAAALAKMLKRIVKQTRKNKLNISNTVIDDDTLLTEMKKLVQILYNQNRMWVVDFNRDMNSYFDLSQAYKLHLYVDLNTVIMFITMPLLKSTTVPFNLYRAMTLPFCRGTMCLLIISGNEYFGITDSKNYYVPVSDNFRQDCQEFTGYNEFLCPETEPIATMNSKVCEIEMFMGRYRDVVYGMCDIRVANYNPKKAYVNTLVDYRKWLYIFPNTNVSVHYYCKDALVQVNTKVLPGVGVMFSTMEQTCSIGITHDVTITVDSRFYVSHSTTYWPKKKFNFNNYIDQNMLLEKATTASFIQTVDNFTRPVLLQLFNKFHIEDYTSASQHSFHQSKIYTNSATPDEDSQDDSNITVVIIIAIVAATFLFCSLLLFLFCCIKKRCHQSNNVTVQYKNNDEFVTICNNLENNRAYINLTNEYDDSDDMPKPLDLLKDDKPLLYPMIIERIK | ^179^VMFLDTVTTNNVSSK^193^ |
| 4 |  | 99 | MSLAAKLIIYNYYAKYNEVHDVYGESYHHHRIVQEYLSESYVNDMSCIERDVTAMRRLKIGSCTFDEAVKMIDAGDSIKSLSHWFSTGETTGIDDNVRKVLEQIDAVVPVSVRVQTGRQIFSLNNFEREISQDMLGCLQIILGRFEHFMRNGKLLHIANVFNPNSDAVGWWYNKFCVITYVHRIMHRSVPAELVPRLSEAVKKFIRLSKSDYDDRLHVDESYNCPRVIAEMYGRFCGIGKEHFSKHKLSCMHILFQYLRGKTTQEEKSFSCYTVIKDFGRQCIDVYRDLKDVFDLLHAHSMSDKDKNSLMDLLCVMDCEEIDVDCFYYIFESFLNNKHLQ | ^248^LSCMHILFQYLRGK^261^ |
| 124 | ODV-E56 | 375 | MSFFTNLRRVNKLYPNQASFLADNTRLLTSTPAGFTNVLNAPSVRNLGNNRYQPGYQLSNNRFVSTSDINRITRNNDVPNIRNVFQGISDPQINSLRQLRRMDNVPDFHYHTKQTRSNAVRQNFPETNVRTPEGVQNALQQNPRLHNHMRTLKVAGVGILLAGGGYLLFTASTLVQDIINAINRTGGSYYVQGRNAGENVESCLLLQRTCRQDRNLAQSDVNICSRDPLLANDSPLLTNMCQGFNYETEKTVCRGSNPAANPNSPQYVDISDLPAGQTIMCIEPYSFSDLVGDLGLDWLLGREGLVGKSSNSSDGIRNKIMPIIMMIGAVLFLGLILYFIYRYMTKGGGGGGGSGGAPTPIVIMQHPASTTAPRR | ^131^TPEGVQNALQQNPRLHNHMR^150^ |
| 104 | Cathepsin | 323 | MNKILFYLFVYAVVKSAAYDPLKAPNYFEEFVHRFNKNYSSEVEKLRRFKIFQHNLNEIINKNQNDSAKYEINKFSDLSKDETIAKYTGLSLPTQTQNFCKVILLDQPPGKGPLEFDWRRLNKVTSVKNQGMCGACWAFATLGSLESQFAIKHNELINLSEQQMIDCDFVDAGCNGGLLHTAFEAIIKMGGVQLESDYPYEADNNNCRMNSNKFLVQVKDCYRYIIVYEEKLKDLLPLVGPIPMAIDAADIVNYKQGIIKYCFDSGLNHAVLLVGYGVENNIPYWTFKNTWGTDWGEDGFFRVQQNINACGMRNELASTAVIY | ^102^VILLDQPPGK^111^ |

**Supplementary Table S2. Host proteins identified in the M1 section of the gel.**

| Protein | Pfam domain | Size  (amino acids) | Sequence | Sequence(s) of corresponding peptide(s) |
| --- | --- | --- | --- | --- |
| BGIBMGA002381-PA | HSC/HSP70 | 649 | MAKAPAVGIDLGTTYSCVGVFQHGKVEIIANDQGNRTTPSYVAFTDTERLIGDAAKNQVAMNPNNTIFDAKRLIGRKFEDATVQADMKHWPFEVVSDGGKPKIKVAYKGEDKTFFPEEVSSMVLTKMKETAEAYLGKTVQNAVITVPAYFNDSQRQATKDAGTISGLNVLRIINEPTAAAIAYGLDKKGTGERNVLIFDLGGGTFDVSILTIEDGIFEVKSTAGDTHLGGEDFDNRMVNHFVQEFKRKYKKDLATNKRALRRLRTACERAKRTLSSSTQASIEIDSLFEGIDFYTSITRARFEELNADLFRSTMEPVEKSLRDAKMDKAQIHDIVLVGGSTRIPKVQKLLQDFFNGKELNKSINPDEAVAYGAAVQAAILHGDKSEEVQDLLLLDVTPLSLGIETAGGVMTTLIKRNTTIPTKQTQTFTTYSDNQPGVLIQVFEGERAMTKDNNLLGKFELTGIPPAPRGVPQIEVTFDIDANGILNVSAIEKSTNKENKITITNDKGRLSKEEIERMVNEAEKYRNEDDKQKETIQAKNALESYCFSMKSTMEDEKLKEKISDSDKQTILDKCNDTIKWLDSNQLADKEEYEHKQKELEGIYNPIITKMYQGAGGVPGGMPGFPGGAPGAGGAAPGAGGAGPTIEEVD | ^37^TTPSYVAFTDTER^49^, ^138^TVQNAVITVPAYFNDSQR^155^, ^160^DAGTISGLNVLR^171^, ^172^IINEPTAAAIAYGLDK^187^, ^302^FEELNADLFR^311^ |
| BGIBMGA011805-PA | Ubiquitin carboxyl-terminal hydrolase | 665 | MPASICDPVTSALRLSLTQDDSQSSSLLDSRLYSSTKSVLLSKIDFEESGDYQSNVLDGLKSKYVVIRPKKTIHDPKPFKKDSASNSQCLENSKSVEDGLPKPKRILFPLEKVQLGWQSPWAAGAGMQNVGNTCYLNSTLQALFHVPALANWLMSESTHTEKCNQQEACVICGMRASLMATQKGGGAPIKPWQVYSKLRLICRHLTPGRQEDAHEFLRYLVEAMEKCYLLRFINSDKLDQYSKETTPLNQILDNSSEDICTSVATTSGVIYGPQLPESILNREKSPLKLTFYRNDERKPMLNSTSTEAPKPIILNSTLSNSENSSNVINSEPWVVKQNGEIEKVIQTPKTINGIDKGQKVDDEKRISFVIKRQNNEDVQIKPKACKSDSNMKLVANNVKVADQPVSISKLLKPFKSPLEKLEEQMNKNDGGELIQKNDSSSSRLVPYDSESSGDERGDRNGDDPTKRIPGKDAGRKPKPSRCESPRRLVVTTKSSWTVSSEKSGPLLSPSLNGDTNKKVKEERGESSQQSPHTSVSSMSPLSDRGELSPGAVTRALQTASHRGYGAPVAAWSGAHSALSRQVFEERREERKRAAESIDDMDCGRVKKFKNFHHFNRFNNNRNGYNPFQEKQNKLHWGGGNFKYRNERRSHHFNKHHNNKYKRFNR | ^15^LSLTQDDSQSSSLLDSR^31^, ^232^FINSDKLDQYSK^243^ |
| BGIBMGA005576-PA | Actin | 344 | MCDEEVAALVVDNGSGMCKAGFAGDDAPRAVFPSIVGRPRHQGVMVGMGQKDSYVGDEAQSKRGILTLKYPIEHGIVTNWDDMEKIWHHTFYNELRVAPEEHPVLLTEAPLNPKANREKMTQIMFETFNTPAMYVAIQAVLSLYASGRTTGIVLDSGDGVSHTVPIYEGYALPHAILRLDWPEKLCYVALDFEQEMATAASSSSLEKSYELPDGQVITIGNERFRCPEALFQPSFLGMEANGIHETTYNSIMKCDVDIRKDLYANTVLSGGTTMYPGIADRMQKEITALAPSTMKIKIIAPPERKYSVWIGGSILASLSTFQQMWISKQEYDESGPSIVHRKCF | ^120^MTQIMFETFNTPAMYVAIQAVLSLYASGR^148^, ^208^SYELPDGQVITIGNER^223^,  ^305^KYSVWIGGSILASLSTFQQMWISK^328^, ^306^YSVWIGGSILASLSTFQQMWISKQEYDESGPSIVHR^341^ |
| BGIBMGA003126-PA | Nup93/Nic96 | 820 | MGDEGFSKLLQQAEQLTTEIEGNEELPRVERSLGQVLEASQELYSRVIQSGANDIQAHLLFGSKGIDLNQISQKLETLSSKRTFEPLQPIADSDIESFLKNEKENAILSLIDEVNKNSLQTTEDQKWEHMLSEWNKEKIKLMNAMIGPSQNWLDLKRTPEPPSIADAPKKFGHSMLDNVEAAYARQVHQYNKYVFQGAKSRTALHQKFAQVAEDFNDPKVKEMWDIINTMANIPALVRDEDPLKARGNPAIQQCLVAQGKKYLQKRYKLYMNDVVRANPAAALRGGEPGTYPLVRGFVGLRLQGQNSQGFTDGVIDERPLWPMVYYCLRSGDPNAALHCLRKAGRDHEEFIAALEEYIRNPEKPLSDKLQTAINFQYRIQVRNSTDPYKRAVYCVIGCCDVSDEHYEVARTADDYLWLKLSFIRTRSANETESFSYSDLQKLILEEYGETHYHAYEKPVVYFQVLALTGQFEPAIEFLSRIPRYQVHGVHMALALHDVYMLGTPRNVQAPLLSVDTEDPPPLRRLNLARLLLLYVRKFELTDPSDALHYYYFLRNLKDPSGKNLFMCCCTDLALESRDYELLFGRIDANSSLRSPGLVDQFNNPHIDSKVIALNVAEQLVNKGLFEDAITMYDIADNLEKVVELSCVLLAQVVNSGGGETGVRGRLAKRCELTDARLRASTETLPPSLLHAYTKLCKLCIFFDQFHADNYDAALETLGECEVVPLNASELEARVAAARSGRGELLRCLPAVLRALCTVLLAHRQRLRANEHSHPLSQQNNKQLEWLREQAEVLNTFAGNIAYRMPGDTYSQLAQMQILMH | ^32^SLGQVLEASQELYSR^46^, ^427^SANETESFSYSDLQK^441^, ^610^VIALNVAEQLVNK^622^ |
| BGIBMGA011746-PA | DEAD/DEAH box helicase | 569 | MRIDNNGNVFVTSGVLSASAKQCIDFFLCTRSGNWNNSRGNGGSKFGGGNKFGDNSSRFGNGGNRFGGSGGFGGKKEFSGGQNMRRPDWDSVSLQPFNKNFYDPHPTVLKRSPYEVEEYRNNHEVTVSGVEVHNPIQYFEEANFPDYVQQGVKTMGYKEPTPIQAQGWPIAMSGKNLVGVAQTGSGKTLAYILPAIVHINNQPPIRRGDGPIALVLAPTRELAQQIQQVAADFGHTSYVRNTCVFGGAPKREQARDLERGVEIVIATPGRLIDFLEKGTTNLQRCTYLVLDEADRMLDMGFEPQIRKIIEQIRPDRQTLMWSATWPKEVKKLAEDYLGDYIQINIGSLQLSANHNILQIVDICQEHEKENKLNVLLQEIGQSQEPGAKTIIFVETKRKAENISRNIRRYGWPAVCMHGDKTQQERDEVLYQFKEGRASILVATDVAARGLDVDGIKYVINFDYPNSSEDYIHRIGRTGRSKSKGTSYAFFTPSNSRQAKDLVSVLQEANQIISPQLQSMADRCGGGGGGWNRNRFGGGGRGGGGSFKRGSNFGKSSQRGGSGYKRFDDY | ^188^TLAYILPAIVHINNQPPIR^206^, ^208^GDGPIALVLAPTR^220^, ^317^QTLMWSATWPK^327^ |
| BGIBMGA000808-PA | XRN 5'-3' exonuclease | 322 | MGVPAFFRWLSRKYPSVIVECVEQKPTDVDGQLVYVDSSLPNPNGVEFDNLYLDMNGIIHPCTHPEDKPAPKDEDEMMVAIFECIDRLFRIVRPRKLLYMAIDGVAPRAKMNQQRSRRFRASKETQEKIEEVARIRNELQAKGAYLPPERPKEAHFDSNCITPGTPFMDRLSKCLHYYIHDRLNNDPGWKGIKVILSDANVPGEGEHKIMDYIRRQRAQPDHDPNTQHVLCGADADLIMLGLATHEPNFTIIREEFKPNKPRPCDVCGQLGHEMKECTGTNPDASVVRSDPAFGNQDNFIFVRLSVLREYLERELQESVLKV | ^97^LLYMAIDGVAPR^108^, ^289^SDPAFGNQDNFIFVR^303^ |
| BGIBMGA005650-PA | CAF1 family ribonuclease | 479 | MEVIRKNFKEALPLAAASVKKADFLVIDTEFTGIINGRDVSMFDTPEEYYKCTQKGSSEFLLIQFGLCAFHWNHKEKHYMNDAYNFYLFPRGSPGPDRMFMCQSSSLDFLASQGFDFNKLIKDGISYMTVPIECKLRENLTERQKTYSKGKDSIKVPDEHKDDLKNHDALKKEREWEDFEDAVGFSKVARMISESEKLVVGHNMLLDVMHTLNHFFQPLPAEYTQFKEFTHCMFPHILDTKYMSSLPPFKDKVNSSILKHLLATLSAAPFSLPKVVSDEGRGYSQLHEKHHEAGYDAYVTGLCFLAMHSHLANMRGDDTTRFLSITSPLIKPFLNKVFLSRTAHQDSPFINLAGPEPLPPRDHVFHLAFPREWQRNEITQLFSPFGPITVQFIDDTSAFVALARREQATSVSKALAKHSKITLTSYYKYKKINDTPSSKITTKTAEAANNDCKKTYAILIISLVVILIAFFLRGFNKLY | ^39^DVSMFDTPEEYYK^51^, ^78^HYMNDAYNFYLFPR^91^, ^322^FLSITSPLIKPFLNK^336^ |
| BGIBMGA011581-PA | Small ubiquitin  -related modifier(SUMO) | 91 | MADEKKGENEHINLKVLGQDNAIVQFKIKKHTPLRKLMNAYCDRAGLSMQVVRFRFDGQPINENDTPTSLEMEEGDTIEVYQQQTGGVSLV | ^16^VLGQDNAIVQFK^27^ |

**Supplementary Table S3. Viral proteins identified in the M2 section of the gel.**

| ORF | Protein | Size(amino acids) | Sequence | Sequence(s) of corresponding peptide(s) |
| --- | --- | --- | --- | --- |
| 1 | polyhedrin | 245 | MPNYSYTPTIGRTYVYDNKYYKNLGCLIKNAKRKKHLVEHEQEEKQWDLLDNYMVAEDPFLGPGKNQKLTLFKEIRSVKPDTMKLIVNWSGKEFLRETWTRFVEDSFPIVNDQEVMDVYLVANLKPTRPNRCYKFLAQHALRWEEDYVPHEVIRIVEPSYVGMNNEYRISLAKKGGGCPIMNIHSEYTNSFESFVNRVIWENFYKPIVYIGTDSAEEEEILIEVSLVFKIKEFAPDAPLFTGPAY | ^1^MPNYSYTPTIGR^12^, ^1^MPNYSYTPTIGRTYVYDNK^19^, ^13^TYVYDNK^19^, ^13^TYVYDNKYYK^22^, ^20^YYKNLGCLIK^29^, ^36^HLVEHEQEEK^45^, ^36^HLVEHEQEEKQWDLLDNYMVAEDPFLGPGK^65^, ^46^QWDLLDNYMVAEDPFLGPGK^65^, ^46^QWDLLDNYMVAEDPFLGPGKNQK^68^, ^69^LTLFKEIR^76 77^SVKPDTMKLIVNWSGK^92^, ^85^LIVNWSGK^92^, ^85^LIVNWSGKEFLR^96^, ^93^EFLRETWTR^101^, ^97^ETWTRFVEDSFPIVNDQEVMDVYLVANLKPTRPNR^131^,^102^FVEDSFPIVNDQEVMDVYLVANLKPTRPNRCYK^134^, ^135^FLAQHALR^142^, ^135^FLAQHALRWEEDYVPHEVIR^154^, ^143^WEEDYVPHEVIR^154^, ^143^WEEDYVPHEVIRIVEPSYVGMNNEYR^168^, ^155^IVEPSYVGMNNEYRISLAK^173^, ^155^IVEPSYVGMNNEYR^168^, ^174^KGGGCPIMNIHSEYTNSFESFVNR^197^, ^175^GGGCPIMNIHSEYTNSFESFVNR^197^, ^230^IKEFAPDAPLFTGPAY^245^, ^232^EFAPDAPLFTGPAY^245^  ^102^FVEDSFPIVNDQEVMDVYLVANLKPTRPNR^131^, ^198^VIWENFYKPIVYIGTDSAEEEEILIEVSLVFK^229^ |
| 37 | ODV-E66 | 702 | MSTVLIIVVVVIFLICFWCLLNSSNNSNNKNDANRNNVFVDFDPLPFDAFDINTFDITNDDSIVAFQQNNIQELENFEQWFKNNLSYSFSQKAEKVVNPNRNWNDNTVFDNLSPWTSVPDFGTVCHTLIGYCVRYNNTSDALYQNPELAYNLINGLRIICSKLPDPPPHQQAPWGPVADWYHFTITMPEVFMNITIVLNETQHYDEAAALTRYWLGLYLPTAVNSMGWHRTAGNSMRMGVPYTYSQMLRGYLLAEIRQEQGIQEILNTIAFPYVTRGNGLHVDSIYIDHIDVRAYGYLINSYFTFAYYTYYFGDEVINTVGLTRAIENVGSPEGVVVPGVMSRNGTLYSNVIGNFITYPLAVHSADYSKVLTKLSKTYYGSVVGVTDRLAYYESDPTNNVQAPLWTMARRIWNRRGRIINYNANTVPFESGVILQSLNGIMRIPSGTTSTQSFRPAIGQTAIAKTDTAGAILVYAKFAEMNNLQFKSCTLFYDHGMFQLYYNIGVEPNSLNNTNGRVIVLSRDTSVNTNDLSFEAQRINNNNSSEGTTFNGVVCHRVPITNINVPSLTVRSPNSSVELVEQIISFQSMYTATASACYKLNVEGHSDSLRAFRVDSDENIYVNVGNGVKALFNYPWVMVKENNMVSFMSANEDTTIQFSVIMNSFTSIGEPALKYSPSNCFAYGNGFKLNDSTFDLQFIFEIV | ^83^NNLSYSFSQK^92^, ^135^YNNTSDALYQNPELAYNLINGLR^157^, ^213^YWLGLYLPTAVNSMGWHR^230^, ^258^QEQGIQEILNTIAFPYVTR^276^, ^277^GNGLHVDSIYIDHIDVR^293^, ^238^MGVPYTYSQMLR^249^, ^325^AIENVGSPEGVVVPGVMSR^343^, ^344^NGTLYSNVIGNFITYPLAVHSADYSK^369^, ^377^TYYGSVVGVTDR^388^, ^389^LAYYESDPTNNVQAPLWTMAR^409^, ^418^IINYNANTVPFESGVILQSLNGIMR^442^, ^443^IPSGTTSTQSFRPAIGQTAIAK^464^, ^465^TDTAGAILVYAK^476^, ^477^FAEMNNLQFK^486^, ^523^DTSVNTNDLSFEAQR^537^, ^538^INNNNSSEGTTFNGVVCHR^556^, ^557^VPITNINVPSLTVR^570^, ^599^LNVEGHSDSLR^609^, ^613^VDSDENIYVNVGNGVK^628^, ^629^ALFNYPWVMVK^639^, ^674^YSPSNCFAYGNGFK^687^ |
| 118 |  | 476 | MSGGGNLLTLERDHFKYLFLTSYFDLKDNEHVPSEPMAFIRNYLNCTFDLLDDAVLMNYFNYLQSMQLKHLVGSTSTNIFKFVKPQFRFVCNRTTVDILEFDTRMYIKPGTHVYATNLFTSNPRKMMAFLYAEFGKVFKNKIFVNINNYGCVLAGSAGFLFDDAYVDWNGVRMCAAPRLDNNMHPFRLYLLGEDMAKHFVDNNILPPHPSNAKTRKINNSMFMLKNFYKGLPLFKLKYTVVNSTKIVTRKPNDIFNEIDKELNGNCPFIKFIQRDYIFDAQFPPDLLDLLNEYMTKSSIMKIITKFVIEENPAMNGEMSREIILDRYSVDNYRKLYIKMEITNQFPVMYDHESSYIFVSKDILQLKGTMNAFYAPKQRILSILAINRLFGATETIDFHPNLLVYRQSSPPVRLTGDVYVVDKNEKVFLVKHVFSNTVPAYLLIRGDYESSSELKSLRDLNPWVQNTLLKLLIPDSV | ^17^YLFLTSYFDLK^27^, ^28^DNEHVPSEPMAFIR^41^, ^70^HLVGSTSTNIFK^81^, ^94^TTVDILEFDTR^104^, ^105^MYIKPGTHVYATNLFTSNPR^124^, ^126^MMAFLYAEFGK^136^, ^188^LYLLGEDMAK^197^, ^198^HFVDNNILPPHPSNAK^213^, ^217^INNSMFMLK^225^, ^250^KPNDIFNEIDK^260^, ^261^ELNGNCPFIK^270^, ^306^FVIEENPAMNGEMSR^320^, ^367^GTMNAFYAPK^376^, ^379^ILSILAINR^387^, ^388^LFGATETIDFHPNLLVYR^405^, ^431^HVFSNTVPAYLLIR^444^, ^458^DLNPWVQNTLLK^469^ |
| 66 | GP41 | 403 | MTDERGNFYYNTPPPPLRYPSNPATAIFTNAQTYNNAPGYVPPTTRDNKMDTSRSNSTNSVAIAPYNKSKEPTLDAGESIWYNKCVDFVQKIIRYYRCNDMSELSPLMIHFINTIRDMCIDTNPINVNVVKRFESEETMIRHLIRLQKELGQGNAAESLPSDSNIFQASFVLNSLPAYAQKFYNGGADMLGKDALAEAAKQLSLAVQYMVAESVTCNIPIPLPFNQQLANNYMTLLLKHATLPPNIQSAVESRRFPHINMINDLINAVIDDLFAGGGDYYHYVLNEKNRARIMSLKENVAFLAPLSASANIFNYMAELATRAGKQPSMFQNATFLTSAANAVNSPAAHLTKNACQDSLTELAFQNETLRRFIFQQINYNKDANAIIAAAAPNVTRPNTKGRTV | ^6^GNFYYNTPPPPLR^18^, ^69^SKEPTLDAGESIWYNK^84^, ^71^EPTLDAGESIWYNK^84^, ^117^DMCIDTNPINVNVVK^131^, ^239^HATLPPNIQSAVESR^253^, ^371^FIFQQINYNK^380^ |
| 88 | VP80 | 692 | MNDSNSLLITRLAAQILSRNIQTVDVIVDDKTLSLEEKIDTLTSMVLAVNGLPQSPPRVSSSDLAASSIKNNSKLVGNDFEMRYNVLRMAVVFVKRYPKYYNETTAGLVAEIENNLLQYQNYVNQGNYQNIQGYDSLLNKAEECYVKIDRLFKESIKKIMDDTEAFEKEQEAERLRAEQTAANALLNRRAQTSADDVVNRADANISTAFSDPLPGSSAPRYMYESSESDTYMEPARHTAEHYTDQDKDYNAAYTADEYNSLVRTVLLRLIEKALATLTNRLHITTIDQLKKFRDYLNSDADAGEFQIFLNQEDCVMLKNLSNLASKFFNVRCVADTLEVMLEALRNNIELVQPESDAVRRIVIKMTQEIKDSTTPLYNIAMYKSDYDAIKNKNIKTLFDLYNDRLPINFLDTSATSPAVRKTPGKRSAEDDLLPTRSNKRANRPEISVISSEDEQEDDDVEDVDYEKESKRRKLEDEDFLKLKALEFSKDIVNEKLQKIIVVTDGMKRLYEYCNCKNSLETLPSAANYGSLLKRLNLYNLDHIEMNVNFYELLFPLTLYNDNDNSDKTLSHQLVNYIFLASNYFQNCAKNFNYMRETFNVFGPFKQIDFMVMFVIKFNFLCDMRNFAKLIDELVPNKQPNMRIHSVLVMRDKIIKLAFSNLQFQTFSKKDKTRNTKHLQRLIMLMNANYNVI | ^201^ADANISTAFSDPLPGSSAPR^220^, ^221^YMYESSESDTYMEPAR^236^, ^237^HTAEHYTDQDKDYNAAYTADEYNSLVR^263^, ^248^DYNAAYTADEYNSLVR^263^, ^332^CVADTLEVMLEALR^345^, ^346^NNIELVQPESDAVR^359^, ^371^DSTTPLYNIAMYK^383^, ^405^LPINFLDTSATSPAVR^420^  ^656^LAFSNLQFQTFSK^668^, ^441^ANRPEISVISSEDEQEDDDVEDVDYEK^467^ |
| 54 |  | 805 | MQRWPKYGGTDVNTRTVHDLLNTINTMSARIKTLERYEHALREIHKVVVILKPSANTHGFEPDALPALIMQFLSDFAGRDINTLTHNINYKYDYNYPQPPAPVPAMQPPPPPPFHQPPAPPQPPYYNNYPYYPPYPFSTQPPESNVAGVGGSQSLNQITLTNEEESELTALLKNMQTNMTWELVQNFVEVLIRIVRVHVVNNVTMINVISSITSVRTLIDYNFTEFIRCVYQKTNIRFAIDQYLCTNIVTFIDFFTRVFYLVTRTNFHFTTFDQLTQYSDELYTKIQTSILNSAAPLSPPTVETVNSDVVISNLQEQLKRERALMQQIVEQHRIANERVETMQSQYDELDLKYKEVFEDKSEFAQQKSENVQKIKQLERSNRELNGTVQKLRDENAEILSEIQLQKGDLDEYKNTNRQLNEDIHRLKKRMESTFDKNYVKTLNDKIESLEKQLDDTQNLNRELESSINETAQTYKFDAKDVMELKQSVSIKDQEIAMKNAQYLELSATYQQTVNELAATKNELSQIATTNQSLFAENEESKVLLEGALAFIDSFYQIIMQIEKPDYVPISKPQLTAQESIYQTDYIKDWLQKLRSKLSNADVANLQSVSEVNDLKTQIISIVPRNIVNRILKENYKVKVENVNAELMENVAVISAVSALVQQYEQSEKQNIRLRKDFEIKLNELQRLLDQNQTDFESISEFISRDPAFNRNLNDERFQNLKQQCDEMSSKYSALKTTKIKEMESIADQAVKSEMSKLNTQLDELNSLFVKYNRKAQDIFEWKTSMLKRYETLARTTAASVQPNVE | ^217^TLIDYNFTEFIR^228^, ^597^LSNADVANLQSVSEVNDLK^615^ |
| 134 |  | 109 | MYSTSKINNARVVASQHDYDRDQIKRELNSLRRNVHDMCTRSGTSFDCNKFLRSDDMTPVVTTITPKRTADYKITEYVSDVKTIKPSNRPLVESGPLVQEAAKYGRCTV | ^54^SDDMTPVVTTITPK^67^, ^83^TIKPSNRPLVESGPLVQEAAK^103^ |
| 75 |  | 259 | MIPLTPLFSRYKDSYLLYSFRLIDLLRASKSAHLTKLLSSQATYLYHFACLMKYKDIQKYEVQQLIEWAINASPDMDLQQFRIEFMDKTTELNLRSCQPKSFTYTFTTIWDTMHFLSLIIDDMVYTRDKSSLDFVMQQLKTMKVLFYNVFFILQCAMCRDHYMNVKGFLIYHIELIEIALDKEKYGTDITFVDSYQQETAGADAAAVSNNMLMKNLMAYVIMTFHNHVNDYKWIQRNQKPPAHYERMTWGEYKKLLNLQ | ^1^MIPLTPLFSR^10^, ^11^YKDSYLLYSFR^21^, ^13^DSYLLYSFR^21^, ^37^LLSSQATYLYHFACLMK^53^, ^130^SSLDFVMQQLK^140^, ^167^GFLIYHIELIEIALDK^182^, ^215^NLMAYVIMTFHNHVNDYK^232^ |
| 26 | Ubiquitin | 77 | MQIFIKTLTGKTITAETEPAETVADLKQKIADKEGVPVDQQRLIFAGKQLEDSKTMADYNIQKESTLHMVLRLRGGY | ^12^TITAETEPAETVADLK^27^ |
| 106 | P24 | 195 | MKTDAQSTSNTHNFMYSPDNNLEVVIITNSDGDHDGYLELTAAAKIMSPFISNGGSTVWTNAAPSHKLIKNNKNYIHVFGLFKYLSNYNLNNKKRPKEYYVLKSIISDLLMGAQGKVFDPLCEVKTQLCAIQESLNEAISTLNVHAAANSPAPDINKLQDMIQDLQSEYNKKITFTTDTILENLKNIKDLMCLNK | ^46^IMSPFISNGGSTVWTNAAPSHK^67^, ^74^NYIHVFGLFK^83^, ^104^SIISDLLMGAQGK^116^, ^126^TQLCAIQESLNEAISTLNVHAAANSPAPDINK^157^, ^173^ITFTTDTILENLK^185^ |
| 63 | VLF-1 | 379 | MNGFNVRNENNFNSWKIKIQSTPRFESVFDLATDRQRCTPDEVKNNSLWSKYMFPKPFAPTTLKSYKSRFIKIVYCSVDDVHLEDMSYSLDMEFDSIENQTLLIDPQELCRRMLELRSVTKETLQLTINFYTNMMNLPEYKIPRMVMLPRDKELKNIRKKEKNLMLKNVIDTILNFINDKIKMLNGDYVHDRGLIRGAIVFCIMLGTGMRINEARQLSVNDLNVLIKKGKLHSDTINLKRKRSRNNTLNNIKMKPLELAREIYSRNPTILQISKNTSTPFKDFRRLLEESGVEMERPRSNMIRHYLSSNLYNSGVPLQKVAKLMNHESSASTKHYLNKYNIGLDETSSEEENNNNDDDAQHNRNSSGSSGESLLYYRNE | ^25^FESVFDLATDR^35^, ^168^NVIDTILNFINDK^180^, ^216^QLSVNDLNVLIK^227^ |
| 108 | PP34 | 315 | MKPTNNVMFDDASVLWIDTDYIYQNLKMPLQAFQQLLFTIPSKHRKMINDAGGSCHNTVKYMVDIYGASVLILRTPCSFADQLLSTFIANNYLCYFYRRRRSRSRSRSRSPHGRPRSRSRSRSRSRSRSSSPRQGRRQIFDALEKIRHQNDMLMNNVNQINLNQTNQFLELSNVMTGVRNQNVQLLAALETAKDVILTRLNTLLSEITDSLPDLTLMLDKLAEQLLEAINTMQQTQRNELNNTNSILTNLASSITNINGTLNNLLTAIENLAGGGGGGGGGGGGNFNEADRQKLDLVHTLVNDIKNILTGTLTTK | ^28^MPLQAFQQLLFTIPSK^43^, ^180^NQNVQLLAALETAK^193^ |
| 87 | P45 | 387 | MCAYRLQYSLRFNTYDRFENVCFEAQLLRDEIDSLCFLFSKYFNQSLIVDGKGLTFFTEFNKCIVSIKSSFENQANNTDNIHNVKNIFSIFLRDEFIKQVPQFRTIMQYLQTYYNPTPAPDVDEIMCQSCKPANKIQCFECKCRYLASSLSTLDEGLQNGWDIFLRPMFGMPLMLYVLLRTDYKNESDVINENNLITQIFVQFFYNLICDKAYSLYTKRDMCVPFVKECKKATIGLRQEDHERVLSILNAQCNGFSTAANGNRLLLPFKNFMIKMGRNTNMKKVNKIASTVLIGFYLRHYLESLPNKAYPVAELELRNVCRFIMSKYSDENINLLIHKLKLIKIDICNVLMTEMIVPESFIRHIITKYQLDNEISLLIELNHDCFNK | ^53^GLTFFTEFNK^62^, ^244^VLSILNAQCNGFSTAANGNR^263^, ^308^AYPVAELELR^317^ |
| 69 | P95 | 839 | MMSGVMLLVFAIFLIIAFTLIYLAIYFKFDETTYTKRLQVMIEYIKRTNADEPTPNVIGYVSDITQNTYTVTWFNTVDLSTYQESVHDDRNEIFDFLNQKLQPVDRIVHDRVRANDENPNEFILSGDKDDVTMKCPAYFNFDYAQLKCVPVPPCDNKPAGRYPMDERLLDTLVLNQHLDKDYSSNEHLYHPTFYLRCFANGAHAVEECPDNYTFDAKTRQCKVNELCENRPDGYILSYFPSNLLVNQFMQCVSGRHVVRECPANKIFDRNLMSCVEAHPCTFNGAGHTYITADISDAQYFKCLNNNESQLMTCINRIRNSDNQYECSGDSRCIDLPNGTGQQVFKHADDDISYNSGQLVCDNFEIISNIECDQSNVFENKLFMDKFRLNMQFPTEVFDGTACVPATADNVNFLRSTFAIENIPNHYDIDMQTSMLGKIEMIKQLVSKDLSLNNDAIFAQWLLYARDKNAIGLNPLTGEPIDCFGNNLYDVFDARRANVCKDLGKSVLKTLNFGDGEFLNVLSDTLTGKDEDYRQFCAISYENGQKIVENEHFQRRILTNILQSDVCANIYTTLYQKYTTLNPKYTTTPLQYNRILVKRPKNIEIYGANTRLKNATIPKNATTISPVFNPFENQPNNRQNDSISPLFNPFQTTDAVWYSEPDGDEAVAPPPTAPPPPSEPEPEPEPELPSPLILDNKDLFYSCHYSVPFFKLTSCHAENDVIINALNELRNNVKVDADCESAKDLSHVLNAYAYVGNGIGCRSAYDGDAIVVKKEAVPSHVYANLNTQSNDGVKYNRWLHVKNDQYMACPEELYDNDEFKCNVESDKLYYLDNLQEDSIV | ^509^TLNFGDGEFLNVLSDTLTGK^528^, ^774^EAVPSHVYANLNTQSNDGVK^793^ |
| 68 |  | 181 | MANTSNITPDIIVNAQINSEDENVLDFIIEDEYYLKKRGVGAHIIKVASSQQLRLLYKNAYSAVSCGNYSILCNLVQNGEYDLNAIMFNCAEIKLNKGQMLFQTKIYRPDNNKTDAAVNTSSPKRAVETENDDDDDEDDAASAIDEQKENTDAVGIDFEENIDDGDVSAPKKQKLDNAEQN | ^126^AVETENDDDDDEDDAASAIDEQK^148^, ^149^ENTDAVGIDFEENIDDGDVSAPK^171^ |
| 2 | orf1629 | 542 | MTNRKYESVQSYLFNNRNNKIDAHQFFERVDTAEAQIIKDSIYDNTVLLNRDVFLNILKFANDVFDNKAYMYVDDSEVSRYYNAVVKMKRLVINVRDPSLRQSLYNTIAYIERLLNIGTVNDSEITMLIADFYDLYSNYNIELPPPPPQALPRSRRPSVVQPAAPAPVPTIVHEQTKPEQIIIPAAPPPPSSVPNIPAPPPPPPPPPSSMSELPPAPPMPTKPQPAAPLDDRQQLLEAIRNEKNRTRLRPVKPKTAPETNTIIEVPTTVLPKEPKPPSASPPPPPPPPAPPAPPPMIDLSSAPLQPPLVDLPAEMLPPPAPSLSNVLSELKSGTVRLKPAQKRPQSEIIPKNSTTKNLIADVLVDTINRRRVAMAKSSSEATSNDEGWDDGGNNRPNAPDVKYVQALFNVFTSSQLYTNDSDEKNTKAHNILNDVESLLQNKTQTNIDKARLLLQDLASRVVLSENPLDSPAIGLQKQPLFETNRNLFYKSIEDLIFKFRYKDAENHLIFALTYHPKDYKFNELLKYVQQLSVNQQRTESNA | ^6^YESVQSYLFNNR^17^, ^40^DSIYDNTVLLNR^51^, ^233^QQLLEAIR^240^, ^255^TAPETNTIIEVPTTVLPK^272^, ^357^NLIADVLVDTINR^369^, ^377^SSSEATSNDEGWDDGGNNRPNAPDVK^402^ |
| 77 | ODV-E25 | 228 | MWKIVLLIVLLVLIYLYWTNALNLNSLTEASPSLGQSSESVESDENKRLNVKLNNARVANLRIAHGDNKLSQMYIAEKPLSIDDIVKEGSNKVGTNSIFLGTVYDYGVKSPNAASTSSNVTMTRGTANFDIKEFKSMFIVFKGITPTKTVEDNGMLRFEVDSMIVCLIDPNTGPLSEREVRELRKSNCTLVYTKNEAAQQVLLENNFTAINADQTAYLKNYKSYREMN | ^70^LSQMYIAEKPLSIDDIVK^87^ |
| 70 | VP15 | 126 | MNTRGCVNNNKMIFMNALGLQPQSKVKIIAHKTLEKFKRDAYTRFKGVKAIKNELKTYNLTLQQYNEALNQCALNDSRWRDTNNWHHDIKEGVKINKRHIYRVNFNSKTKEIKEYYYIKVECYVNN | ^57^TYNLTLQQYNEALNQCALNDSR^78^ |
| 11 |  | 110 | MNSGDDDATRLHYSNAHAKQTLATLFANRNHSSFYEYAITFVSTLLFKNNLNLMVACNLINTLINFEINVFGKSLLLSKFVNFCIANSDGVTIQHKMLTNVLSFLLEKYY | ^80^FVNFCIANSDGVTIQHK^96^ |
| 80 | BRO-b | 239 | MAQVKIGQFKFGQDEFTLRYVLGDEQPVKFVAKDIARSLKYVNYEKAVRVHVDVKYKTTYEQACINISKENRVKHGDPLYLSPQTILLDKIGVIQLFMRSKMHNAAELQNWFYEHVLPQCTASALSLLQDAQATVKFNSAPVEGHFYAATTLLYAERNLFKIGQTTNLTRRLATLNCGRADDDQMQYVLQTEPTVHHTLLEKLMKQELRPYRNSGEVYCTDFEHIKRALESCLPHCSQN | ^162^IGQTTNLTR^170^, ^162^IGQTTNLTRR^171^ |
| 30 | P43 | 362 | MDKRANSRKPFLFYNEDYYCEKPKRYFHTNKVIFEKLDPYATNINRCRKLLTDFFDYCLPKYYRRKNKFALLFRLLEPVIKQTGTSSALTAVSDQSRWLEINQFSAWERRDNQYAHKWLIKVAGADMGQQILFIIKQVTKKFKTCNLGFHNYYKLFRRCLSMLLFKHKEVFIKCLQVILKAAMPVKNKGVVKSNYAFAVTNALHYYIVDNPHLLCKDINVAIKVRRLLIKHEMLPTEKRIRLSFEKCSKGIEVPLYEKLLLNHMMRINDDNLQWPSLMNNKKIMEWNANRGFDESNKILHVYIGQYYKSSCRRIKKSFFKYNGWNEQLRFCRTEKFCSLVNLQLNKDGSKKLKRVQRKCDKL | ^122^VAGADMGQQILFIIK^136^ |
| 92a |  | 59 | MKYFLSAIFLIIVFMYAMYFCISIVVNNGRVQRDLFYHYNYVPDTLLNTVRVHKLKSKV | ^3^YFLSAIFLIIVFMYAMYFCISIVVNNGR^30^ |
| 131 | BRO-d | 349 | MAQVKIGQFKFGQDTFTLRYVLEQGNPQVKFVAKDIASSLKYGNCKDAVSRHVDKKYKYTYSESGARLPPSAPNSVAKQGDPLYLQPHTVLITKSGVIQLIMKSKLPYAIELQEWLLEEVIPQVLCTGKYDPAIKQQEEKNKQLVTKLIATFTEHTNALQAVVAQKTEELFKKQEFIERIIAIKDKQIEAKDLQVTRVMTDLNRMYTGFQETMQRKDEMMHKKDELLQVKDTQVSNLIAKMIDLSDRAVQYPADKRKHPVLCVTRDGTTFTAITGQKTYVESQKHKRNIDAANIVVENIRPNPTVDWNNATDRLQSKRSKRSISFDSLEEAQQFENRIKYLLKNANNIN | ^31^FVAKDIASSLK^41^, ^257^KHPVLCVTR^265^ |
| 67 |  | 234 | MTTTTTKTTQPPLSMSKKKTPTLLESISKKISTTETFQRLRNKNLTTLNKIKYDSELLLHYLYDDQQNKNSDYANNNINVIKISKVKVKKTGASILAHYFAQVHVSNGYSFEFHPGSQPRTFQTIHTDGLIIKVLILCDECCKKELRDYIKGENSFNVAFKNCESILCRRISFQTVLMTCAVLLLLFNVEKFSMINLLIILLILLSLFCHNNYIISNPCIEFCNHKSTNKKYDR | ^30^KISTTETFQR^39^ |
| 27 | 39K | 277 | MVNMPEQQSSTETAAVCKNEKLLNKLESSSYNKSNMDQLIAIVNFLEKKNINYILNVMPVMQDERKMSKRKKKVINNNKYILFNSWYTKIKQPEWPSSPAMWDLVKNTPELADFVFIFDHTEKMGKKMADRSTSSSSSDNAAIPASKKRQTAVLTNANLAELKESCEMRDKLYSEFYSLLNETFNNNVAPLLSSIYDEVLTRDFITKNMAKFKTVALKLPVAPSTTEYVPTSISGSKKRKNSVPAKQRSSIKNRRNTTAAPTLLMVSDNTQDTNMSE | ^34^SNMDQLIAIVNFLEK^48^ |
| 55 | LEF-3 | 385 | MATKRFFSGESSGEPLIKRMAMANSPKKIRENYKRINGKLMGKMTLSIDNEYYYTFRIMSDNKIQEYYGDSQSFKDMEEGKCYDISLNYVKTKFSQMIQINEYKECEMEIETAIPLSAYLTNKHFENEDSVNIIVKYKFIYKKINSNLYKIVFEVAYKNFNDDSDVVQVECFVNAKTLMNLFKNNIKGSDDINEVFKYLKDNENQIFTIYNIKCQQIFNGANVYMNWNVVNSTRIELCEAKENEAYSNLQNCTNARINISRSNKRVASYNVNMLKSELEENDMSDNKFIVQFKSDDLNVADSDDRSTSSDSSKWNKSVFYVNTNKKTEADSLQKLCADFNQISMLLEDNLIKVTIYVTVENGENGNMNVLGLLKYDEDENDYTFL | ^144^INSNLYKIVFEVAYK^158^ |
| 78 | Helicase | 1222 | MIDNILQFFLKDVPEDKTYEINNLQDANHLIIRNTRTGTRRLFEYVNNFQQFLNTIRNNFNGPCAKHDMGSSCEDTEEATEKQAVQQTLDGHDWVLESNDFCIFVKPFILKKHYEVIQKYINFENFFKSTDPGYINKCVQAGDYYYWPNWPKGQAFSFNGWQLFLNIKFGIVIEPTIPIIHNKKLGPVDLFVFDPKYFLNVELSLRTNHDPPQTLFVNGKTKFDESHEDLFILKMADGTVVTCKVNGELVNSDKNFFNYIRDDINLEECITVPKYKHIVNVNLKSLRVFEDNNFDKNDVDLSDTRSRKPRIVPIISASSENADYIQTQINLGLIAIYENMVKVLATHEQANDPNLLQQYFEKSKFKNFDFLIYVLWKILTKNENFSYRETDIKLFLELLCESLFACDKEILNEALKRCEPYKKQEKVVFNRTCNHWFDFDDTKLCVSLGYYYGIHYMIYLTLSAKNETLDDDELWAYTYENVMALNLPPDIVCKGFFRKLENVVTGVNLVFNGKNYQIVKKEDDLFKLVKSNCYKLSNIKFNNWKYLYLTTYGVYNLFTNSFHSNCPFLLGTTLPQTLKKPTDEEYLPEDAFNYMLSTSTDELSIYRIYHIAKMCRDVKMLKTNMAIVNYMGNCNTCQADMRVALNNLFRDLWNLDDENLITLALYVNKNKVSDMLHNLKCKPCRSTVSGSRPKCKCYKKIKINRKALKVCLIADMFGNDAELSKLIWMLIFTNKTYVSTTLIRTNSEFVNQHGEFFFKEHNKIIQYLYQTIHKIEYVDMLMDKFNDKRLFLTELRDDVAREPDVQFEESDNICKFYTHHTDALIILKKYNVWWDKIILARSTDDLPTWLTRFYMRIIMSKMDLKEYSYNYLKKIVEGYLYFKRFTNFNHANAIMLMHFAASLAIPVDYGKKAIYMPGEPGSGKSSFFELLDYLVLMHKFDDDNHSGESNKETSDKEVSKLNSQLYVINELKQCSESYFKKHADSSKSDSKSRKYQGLLKYEANYKMLIVNNKPLYVDDYDDGVQDRFLIVYTNHKFVDSVKFAGSVYEHIKSKQFPIESMYYESLVTPVRLFLSHVLMYRRDPKTGFVVYKTLLNNDPMHKHNLMCMSTNNSPLYALIYILNIKTVRNATITIGEDKMEEMISIAVQHLKNFLHPSFVQYNYKKNINASSSKSFVFNEQVLLQQIKNKFKNNYNKTTNVFYNMTMALNRNDLNTSVPNFVC | ^643^VALNNLFR^650^ |

**Supplementary Table S4. Host proteins identified in the M2 section of the gel.**

| Protein | Pfam domain | Size(amino acids) | Sequence | Sequence(s) of corresponding peptide(s) |
| --- | --- | --- | --- | --- |
| BGIBMGA002381-PA | HSC/HSP70 | 649 | MAKAPAVGIDLGTTYSCVGVFQHGKVEIIANDQGNRTTPSYVAFTDTERLIGDAAKNQVAMNPNNTIFDAKRLIGRKFEDATVQADMKHWPFEVVSDGGKPKIKVAYKGEDKTFFPEEVSSMVLTKMKETAEAYLGKTVQNAVITVPAYFNDSQRQATKDAGTISGLNVLRIINEPTAAAIAYGLDKKGTGERNVLIFDLGGGTFDVSILTIEDGIFEVKSTAGDTHLGGEDFDNRMVNHFVQEFKRKYKKDLATNKRALRRLRTACERAKRTLSSSTQASIEIDSLFEGIDFYTSITRARFEELNADLFRSTMEPVEKSLRDAKMDKAQIHDIVLVGGSTRIPKVQKLLQDFFNGKELNKSINPDEAVAYGAAVQAAILHGDKSEEVQDLLLLDVTPLSLGIETAGGVMTTLIKRNTTIPTKQTQTFTTYSDNQPGVLIQVFEGERAMTKDNNLLGKFELTGIPPAPRGVPQIEVTFDIDANGILNVSAIEKSTNKENKITITNDKGRLSKEEIERMVNEAEKYRNEDDKQKETIQAKNALESYCFSMKSTMEDEKLKEKISDSDKQTILDKCNDTIKWLDSNQLADKEEYEHKQKELEGIYNPIITKMYQGAGGVPGGMPGFPGGAPGAGGAAPGAGGAGPTIEEVD | ^37^TTPSYVAFTDTER^49^, ^89^HWPFEVVSDGGKPK^102^, ^113^TFFPEEVSSMVLTK^126^, ^138^TVQNAVITVPAYFNDSQR^155^,  ^160^DAGTISGLNVLR^171^, ^172^IINEPTAAAIAYGLDK^187^, ^221^STAGDTHLGGEDFDNR^236^, ^237^MVNHFVQEFK^246^, ^302^FEELNADLFR^311^, ^329^AQIHDIVLVGGSTR^342^, ^349^LLQDFFNGK^357^, ^362^SINPDEAVAYGAAVQAAILHGDK^384^, ^424^QTQTFTTYSDNQPGVLIQVFEGER^447^, ^459^FELTGIPPAPR^469^, ^540^NALESYCFSMK^550^, ^598^ELEGIYNPIITK^609^ |
| BGIBMGA011746-PA | DEAD/DEAH box helicase | 569 | MRIDNNGNVFVTSGVLSASAKQCIDFFLCTRSGNWNNSRGNGGSKFGGGNKFGDNSSRFGNGGNRFGGSGGFGGKKEFSGGQNMRRPDWDSVSLQPFNKNFYDPHPTVLKRSPYEVEEYRNNHEVTVSGVEVHNPIQYFEEANFPDYVQQGVKTMGYKEPTPIQAQGWPIAMSGKNLVGVAQTGSGKTLAYILPAIVHINNQPPIRRGDGPIALVLAPTRELAQQIQQVAADFGHTSYVRNTCVFGGAPKREQARDLERGVEIVIATPGRLIDFLEKGTTNLQRCTYLVLDEADRMLDMGFEPQIRKIIEQIRPDRQTLMWSATWPKEVKKLAEDYLGDYIQINIGSLQLSANHNILQIVDICQEHEKENKLNVLLQEIGQSQEPGAKTIIFVETKRKAENISRNIRRYGWPAVCMHGDKTQQERDEVLYQFKEGRASILVATDVAARGLDVDGIKYVINFDYPNSSEDYIHRIGRTGRSKSKGTSYAFFTPSNSRQAKDLVSVLQEANQIISPQLQSMADRCGGGGGGWNRNRFGGGGRGGGGSFKRGSNFGKSSQRGGSGYKRFDDY | ^86^RPDWDSVSLQPFNK^99^, ^188^TLAYILPAIVHINNQPPIR^206^, ^208^GDGPIALVLAPTR^220^, ^221^ELAQQIQQVAADFGHTSYVR^240^, ^296^MLDMGFEPQIR^306^, ^457^YVINFDYPNSSEDYIHR^473^ |
| BGIBMGA011965-PA | DEAD/DEAH box helicase | 628 | MTIDVVAVVEEVGVAEGEDPAAVVGVATGRKSADLVDLAEVVVGTVDAAVGMAAVEDETAAGVLGEWGIGMIAPISKMISLAKNFYVPHPNVQRRTQSEIEAYRSQHQITVKGRDVPAPSIFFEEGGFPDYAMKEILKQGFPNPTPIQAQGWPIALSGRDMVGIAQTGSGKTLAYILPAIVHIINQPRLLRDDGPIVLVLAPTRELAQQIQQVANEFGQSIHVRNTCIFGGAPKGPQGRCLERGVEIVIATPGRLIDFLEKETTNLRRCTYLVLDEADRMLDMGFEPQIRKIIEQIRPDRQVLMWSATWPREVQNLAEEFLHDYIQINIGSLELSANHNILQIVDVCEEWEKNDKLITLLTEISSEEETKTIIFAETKRRVDDICKAINRAGWRALAIHGDKNQQDRDYVLNQFRHHAAAILVATDVAARGLDVEDVKFVINYDYPNNSEDYVHRIGRTGRSQNTGTAYTLFTPNNSAKARDLMSVLQEANQVVNPKLLELAQCGMGFKGKFGRSRYRDDGGRGRDGGSRGGRGSRGGGRGGYGDRGPKDCAGRYDAVRSCSGKLVTLIQLRLKIMFRGGMQVVRRGLVNTLHQMSRELISQSKKFLGACAGHNIDDGPASSFNCGAT | ^139^QGFPNPTPIQAQGWPIALSGR^159^, ^172^TLAYILPAIVHIINQPR^188^, ^205^ELAQQIQQVANEFGQSIHVR^224^, ^280^MLDMGFEPQIR^290^, ^439^FVINYDYPNNSEDYVHR^455^ |
| BGIBMGA011805-PA | Ubiquitin carboxyl-terminal hydrolase | 665 | MPASICDPVTSALRLSLTQDDSQSSSLLDSRLYSSTKSVLLSKIDFEESGDYQSNVLDGLKSKYVVIRPKKTIHDPKPFKKDSASNSQCLENSKSVEDGLPKPKRILFPLEKVQLGWQSPWAAGAGMQNVGNTCYLNSTLQALFHVPALANWLMSESTHTEKCNQQEACVICGMRASLMATQKGGGAPIKPWQVYSKLRLICRHLTPGRQEDAHEFLRYLVEAMEKCYLLRFINSDKLDQYSKETTPLNQILDNSSEDICTSVATTSGVIYGPQLPESILNREKSPLKLTFYRNDERKPMLNSTSTEAPKPIILNSTLSNSENSSNVINSEPWVVKQNGEIEKVIQTPKTINGIDKGQKVDDEKRISFVIKRQNNEDVQIKPKACKSDSNMKLVANNVKVADQPVSISKLLKPFKSPLEKLEEQMNKNDGGELIQKNDSSSSRLVPYDSESSGDERGDRNGDDPTKRIPGKDAGRKPKPSRCESPRRLVVTTKSSWTVSSEKSGPLLSPSLNGDTNKKVKEERGESSQQSPHTSVSSMSPLSDRGELSPGAVTRALQTASHRGYGAPVAAWSGAHSALSRQVFEERREERKRAAESIDDMDCGRVKKFKNFHHFNRFNNNRNGYNPFQEKQNKLHWGGGNFKYRNERRSHHFNKHHNNKYKRFNR | ^15^LSLTQDDSQSSSLLDSR^31^ |
| BGIBMGA005650-PA | CAF1 family ribonuclease | 479 | MEVIRKNFKEALPLAAASVKKADFLVIDTEFTGIINGRDVSMFDTPEEYYKCTQKGSSEFLLIQFGLCAFHWNHKEKHYMNDAYNFYLFPRGSPGPDRMFMCQSSSLDFLASQGFDFNKLIKDGISYMTVPIECKLRENLTERQKTYSKGKDSIKVPDEHKDDLKNHDALKKEREWEDFEDAVGFSKVARMISESEKLVVGHNMLLDVMHTLNHFFQPLPAEYTQFKEFTHCMFPHILDTKYMSSLPPFKDKVNSSILKHLLATLSAAPFSLPKVVSDEGRGYSQLHEKHHEAGYDAYVTGLCFLAMHSHLANMRGDDTTRFLSITSPLIKPFLNKVFLSRTAHQDSPFINLAGPEPLPPRDHVFHLAFPREWQRNEITQLFSPFGPITVQFIDDTSAFVALARREQATSVSKALAKHSKITLTSYYKYKKINDTPSSKITTKTAEAANNDCKKTYAILIISLVVILIAFFLRGFNKLY | ^39^DVSMFDTPEEYYK^51^, ^173^EREWEDFEDAVGFSK^187^  ^78^HYMNDAYNFYLFPR^91^ |
| BGIBMGA008447-PA | Nuclear transport factor 2; Nuclear RNA export factor 1 | 613 | MPKRGGGRNRNWKADTDHFEHDDRVNSVPRHVSFKPGAKGKNKFRTWKNPLALLDDDIDMGSSALQGPVRKSSFRGRGRMYSPAPQGSHGHTKKKFIPGVLPWYQIIIPYGAKHEKDVILRALLGFLSPDIFIPHYYKVNGNAAVFYVDDVKTAEKLYNADRKITMSDGFKLIVIVRNAVPNMAIDAAMKEKMKLTMAKRYNATTKALDLTKFHADPDLEDIFCALFRPMIMLAAIDIIAENIPDIEALNLNDNKLHGLQHLKVLSSKLKNLKILYMGDNRIPFLGSLDPIRSLPLVELYLKGNPLVNRFHDHEIYISDVRKKFPKLTRLDGADLPPAIGFDVAEDMGLPPRQQSFLVDPAGQDLVREFLTQYYAIYDSESRQPLLEAYHENATMSMAANYLTNDSRNSTTTRLNSYISNSRNLTRITERESRRRYLRTGKLQVVSFLSDLPKTTHDLMGFAVDLLVFTPAMIVLTMNGVFKETNTPNNPMRSFHRTFVIVSNANGGFSIINDMLYITNTTKEQEDKAFSTGTVEPIPPTPAPTPTPTPTLAAGTPDDSQKMQLLNMLGQQTGMNEHWSINCLQETGWDLQRAMFIFNQLHSEGKIPPEAFVK | ^96^FIPGVLPWYQIIIPYGAK^113^, ^442^LQVVSFLSDLPK^453^ |
| BGIBMGA011581-PA | Small ubiquitin-related modifier(SUMO) | 91 | MADEKKGENEHINLKVLGQDNAIVQFKIKKHTPLRKLMNAYCDRAGLSMQVVRFRFDGQPINENDTPTSLEMEEGDTIEVYQQQTGGVSLV | ^16^VLGQDNAIVQFK^27^ |

**Supplementary Table S5. Viral proteins identified in the M3 section of the gel.**

| ORF | Protein | Size(amino acids) | Sequence | Sequence(s) of corresponding peptide(s) |
| --- | --- | --- | --- | --- |
| 1 | polyhedrin | 245 | MPNYSYTPTIGRTYVYDNKYYKNLGCLIKNAKRKKHLVEHEQEEKQWDLLDNYMVAEDPFLGPGKNQKLTLFKEIRSVKPDTMKLIVNWSGKEFLRETWTRFVEDSFPIVNDQEVMDVYLVANLKPTRPNRCYKFLAQHALRWEEDYVPHEVIRIVEPSYVGMNNEYRISLAKKGGGCPIMNIHSEYTNSFESFVNRVIWENFYKPIVYIGTDSAEEEEILIEVSLVFKIKEFAPDAPLFTGPAY | ^1^MPNYSYTPTIGR^12^, ^13^TYVYDNK^19^, ^13^TYVYDNKYYK^22^, ^20^YYKNLGCLIK^29^, ^36^HLVEHEQEEK^45^, ^36^HLVEHEQEEKQWDLLDNYMVAEDPFLGPGK^65^, ^46^QWDLLDNYMVAEDPFLGPGK^65^, ^46^QWDLLDNYMVAEDPFLGPGKNQK^68^, ^69^LTLFKEIR^76 77^SVKPDTMKLIVNWSGK^92^, ^85^LIVNWSGK^92^, ^85^LIVNWSGKEFLR^96^, ^93^EFLRETWTR^101^, ^102^FVEDSFPIVNDQEVMDVYLVANLKPTRPNRCYK^134^, ^132^CYKFLAQHALR^142^, ^135^FLAQHALR^142^, ^135^FLAQHALRWEEDYVPHEVIR^154^, ^143^WEEDYVPHEVIR^154^, ^143^WEEDYVPHEVIRIVEPSYVGMNNEYR^168^, ^155^IVEPSYVGMNNEYRISLAK^173^, ^155^IVEPSYVGMNNEYR^168^, ^174^KGGGCPIMNIHSEYTNSFESFVNR^197^, ^175^GGGCPIMNIHSEYTNSFESFVNR^197^, ^230^IKEFAPDAPLFTGPAY^245^, ^232^EFAPDAPLFTGPAY^245^  ^102^FVEDSFPIVNDQEVMDVYLVANLKPTRPNR^131^, ^198^VIWENFYKPIVYIGTDSAEEEEILIEVSLVFK^229^ |
| 66 | GP41 | 403 | MTDERGNFYYNTPPPPLRYPSNPATAIFTNAQTYNNAPGYVPPTTRDNKMDTSRSNSTNSVAIAPYNKSKEPTLDAGESIWYNKCVDFVQKIIRYYRCNDMSELSPLMIHFINTIRDMCIDTNPINVNVVKRFESEETMIRHLIRLQKELGQGNAAESLPSDSNIFQASFVLNSLPAYAQKFYNGGADMLGKDALAEAAKQLSLAVQYMVAESVTCNIPIPLPFNQQLANNYMTLLLKHATLPPNIQSAVESRRFPHINMINDLINAVIDDLFAGGGDYYHYVLNEKNRARIMSLKENVAFLAPLSASANIFNYMAELATRAGKQPSMFQNATFLTSAANAVNSPAAHLTKNACQDSLTELAFQNETLRRFIFQQINYNKDANAIIAAAAPNVTRPNTKGRTV | ^6^GNFYYNTPPPPLR^18^, ^19^YPSNPATAIFTNAQTYNNAPGYVPPTTR^46^, ^55^SNSTNSVAIAPYNK^68^, ^69^SKEPTLDAGESIWYNK^84^, ^71^EPTLDAGESIWYNK^84^, ^98^CNDMSELSPLMIHFINTIR^116^, ^117^DMCIDTNPINVNVVK^131^, ^133^FESEETMIR^141^, ^182^FYNGGADMLGK^192^, ^239^HATLPPNIQSAVESR^253^, ^297^ENVAFLAPLSASANIFNYMAELATR^321^, ^325^QPSMFQNATFLTSAANAVNSPAAHLTK^351^, ^352^NACQDSLTELAFQNETLR^369^, ^371^FIFQQINYNK^380^, ^381^DANAIIAAAAPNVTRPNTK^399^ |
| 108 | PP34 | 315 | MKPTNNVMFDDASVLWIDTDYIYQNLKMPLQAFQQLLFTIPSKHRKMINDAGGSCHNTVKYMVDIYGASVLILRTPCSFADQLLSTFIANNYLCYFYRRRRSRSRSRSRSPHGRPRSRSRSRSRSRSRSSSPRQGRRQIFDALEKIRHQNDMLMNNVNQINLNQTNQFLELSNVMTGVRNQNVQLLAALETAKDVILTRLNTLLSEITDSLPDLTLMLDKLAEQLLEAINTMQQTQRNELNNTNSILTNLASSITNINGTLNNLLTAIENLAGGGGGGGGGGGGNFNEADRQKLDLVHTLVNDIKNILTGTLTTK | ^28^MPLQAFQQLLFTIPSK^43^, ^148^HQNDMLMNNVNQINLNQTNQFLELSNVMTGVR^179^, ^180^NQNVQLLAALETAK^193^, ^221^LAEQLLEAINTMQQTQR^237^, ^292^QKLDLVHTLVNDIK^305^, ^294^LDLVHTLVNDIK^305^, ^306^ NILTGTLTTK^315^ |
| 92 |  | 391 | MECPFQIQVCISDRFFAFPHNLVEPQSDVGNKLIENLIVYVPTDDDRLYIDKKQFPKFNSVLVYRHEHDVNIDSRSPKKTASATIVYWNPLMPITEIGAGETRVFSVLLTNNLFYCNTMIIQHENPKCPIEFTYPETDMQSACSALLKNRNGQSVPPPIKSNLRPIACEIPLSHFKELVESNDFLLCFNLETSTMVKILSLKRIFCIFQYRKQPARYVINLPHEEIDNLYNKLNWERTRRLMKGDVPSNCATVNRSSLKYIKQAQSLLGIPDYSQTVVDFVKMFQKIIFPYQLVPNVIIKLNNFDQIMSVAPNKAESYKKIRLFCKNDSIAISSNGIVPINMPDFSPSNNAFDYSDYVNRTNINFVTQRVLIDGGFSSGVTVTPIKYNYYL | ^15^FFAFPHNLVEPQSDVGNK^32^, ^58^FNSVLVYR^65^, ^79^KTASATIVYWNPLMPITEIGAGETR^103^, ^80^TASATIVYWNPLMPITEIGAGETR^103^, ^104^VFSVLLTNNLFYCNTMIIQHENPK^127^, ^128^CPIEFTYPETDMQSACSALLK^148^, ^161^SNLRPIACEIPLSHFK^176^, ^203^RIFCIFQYR^211^, ^204^IFCIFQYR^211^, ^217^YVINLPHEEIDNLYNK^232^, ^263^QAQSLLGIPDYSQTVVDFVK^282^, ^287^IIFPYQLVPNVIIK^300^, ^301^LNNFDQIMSVAPNK^314^, ^361^TNINFVTQR^369^, ^370^VLIDGGFSSGVTVTPIK^386^ |
| 37 | ODV-E66 | 702 | MSTVLIIVVVVIFLICFWCLLNSSNNSNNKNDANRNNVFVDFDPLPFDAFDINTFDITNDDSIVAFQQNNIQELENFEQWFKNNLSYSFSQKAEKVVNPNRNWNDNTVFDNLSPWTSVPDFGTVCHTLIGYCVRYNNTSDALYQNPELAYNLINGLRIICSKLPDPPPHQQAPWGPVADWYHFTITMPEVFMNITIVLNETQHYDEAAALTRYWLGLYLPTAVNSMGWHRTAGNSMRMGVPYTYSQMLRGYLLAEIRQEQGIQEILNTIAFPYVTRGNGLHVDSIYIDHIDVRAYGYLINSYFTFAYYTYYFGDEVINTVGLTRAIENVGSPEGVVVPGVMSRNGTLYSNVIGNFITYPLAVHSADYSKVLTKLSKTYYGSVVGVTDRLAYYESDPTNNVQAPLWTMARRIWNRRGRIINYNANTVPFESGVILQSLNGIMRIPSGTTSTQSFRPAIGQTAIAKTDTAGAILVYAKFAEMNNLQFKSCTLFYDHGMFQLYYNIGVEPNSLNNTNGRVIVLSRDTSVNTNDLSFEAQRINNNNSSEGTTFNGVVCHRVPITNINVPSLTVRSPNSSVELVEQIISFQSMYTATASACYKLNVEGHSDSLRAFRVDSDENIYVNVGNGVKALFNYPWVMVKENNMVSFMSANEDTTIQFSVIMNSFTSIGEPALKYSPSNCFAYGNGFKLNDSTFDLQFIFEIV | ^135^YNNTSDALYQNPELAYNLINGLR^157^, ^213^YWLGLYLPTAVNSMGWHR^230^, ^258^QEQGIQEILNTIAFPYVTR^276^, ^238^MGVPYTYSQMLR^249^, ^325^AIENVGSPEGVVVPGVMSR^343^, ^344^NGTLYSNVIGNFITYPLAVHSADYSK^369^, ^377^TYYGSVVGVTDR^388^, ^389^LAYYESDPTNNVQAPLWTMAR^409^, ^418^IINYNANTVPFESGVILQSLNGIMR^442^, ^443^IPSGTTSTQSFRPAIGQTAIAK^464^, ^465^TDTAGAILVYAK^476^, ^477^FAEMNNLQFK^486^, ^523^DTSVNTNDLSFEAQR^537^, ^538^INNNNSSEGTTFNGVVCHR^556^, ^557^VPITNINVPSLTVR^570^, ^629^ALFNYPWVMVK^639^, ^674^YSPSNCFAYGNGFK^687^ |
| 87 | P45 | 387 | MCAYRLQYSLRFNTYDRFENVCFEAQLLRDEIDSLCFLFSKYFNQSLIVDGKGLTFFTEFNKCIVSIKSSFENQANNTDNIHNVKNIFSIFLRDEFIKQVPQFRTIMQYLQTYYNPTPAPDVDEIMCQSCKPANKIQCFECKCRYLASSLSTLDEGLQNGWDIFLRPMFGMPLMLYVLLRTDYKNESDVINENNLITQIFVQFFYNLICDKAYSLYTKRDMCVPFVKECKKATIGLRQEDHERVLSILNAQCNGFSTAANGNRLLLPFKNFMIKMGRNTNMKKVNKIASTVLIGFYLRHYLESLPNKAYPVAELELRNVCRFIMSKYSDENINLLIHKLKLIKIDICNVLMTEMIVPESFIRHIITKYQLDNEISLLIELNHDCFNK | ^30^DEIDSLCFLFSK^41^, ^42^YFNQSLIVDGK^52^, ^53^GLTFFTEFNK^62^, ^69^SSFENQANNTDNIHNVK^85^, ^86^NIFSIFLRDEFIK^98^, ^244^VLSILNAQCNGFSTAANGNR^263^, ^264^LLLPFK^269^, ^287^IASTVLIGFYLR^298^, ^299^HYLESLPNK^307^, ^308^AYPVAELELR^317^, ^327^YSDENINLLIHK^338^, ^344^IDICNVLMTEMIVPESFIR^362^ |
| 118 |  | 476 | MSGGGNLLTLERDHFKYLFLTSYFDLKDNEHVPSEPMAFIRNYLNCTFDLLDDAVLMNYFNYLQSMQLKHLVGSTSTNIFKFVKPQFRFVCNRTTVDILEFDTRMYIKPGTHVYATNLFTSNPRKMMAFLYAEFGKVFKNKIFVNINNYGCVLAGSAGFLFDDAYVDWNGVRMCAAPRLDNNMHPFRLYLLGEDMAKHFVDNNILPPHPSNAKTRKINNSMFMLKNFYKGLPLFKLKYTVVNSTKIVTRKPNDIFNEIDKELNGNCPFIKFIQRDYIFDAQFPPDLLDLLNEYMTKSSIMKIITKFVIEENPAMNGEMSREIILDRYSVDNYRKLYIKMEITNQFPVMYDHESSYIFVSKDILQLKGTMNAFYAPKQRILSILAINRLFGATETIDFHPNLLVYRQSSPPVRLTGDVYVVDKNEKVFLVKHVFSNTVPAYLLIRGDYESSSELKSLRDLNPWVQNTLLKLLIPDSV | ^17^YLFLTSYFDLK^27^, ^28^DNEHVPSEPMAFIR^41^, ^70^HLVGSTSTNIFK^81^, ^94^TTVDILEFDTR^104^, ^105^MYIKPGTHVYATNLFTSNPR^124^, ^126^MMAFLYAEFGK^136^, ^188^LYLLGEDMAK^197^, ^198^HFVDNNILPPHPSNAK^213^, ^250^KPNDIFNEIDK^260^, ^261^ELNGNCPFIK^270^, ^306^FVIEENPAMNGEMSR^320^, ^367^GTMNAFYAPK^376^, ^379^ILSILAINR^387^, ^388^LFGATETIDFHPNLLVYR^405^, ^431^HVFSNTVPAYLLIR^444^, ^458^DLNPWVQNTLLK^469^ |
| 134 |  | 109 | MYSTSKINNARVVASQHDYDRDQIKRELNSLRRNVHDMCTRSGTSFDCNKFLRSDDMTPVVTTITPKRTADYKITEYVSDVKTIKPSNRPLVESGPLVQEAAKYGRCTV | ^54^SDDMTPVVTTITPK^67^, ^83^TIKPSNRPLVESGPLVQEAAK^103^ |
| 94 |  | 424 | MRKYALLQKMIINELLFLNDNVNYATNKLFSKDQANGELQKLSAMLLNYKKSNKNVPNIKFDLKNLSFMLENTDKIDIIQFDDVKNYVQPAIVNLFESHNRSLNNYSTELNTLLENGNENLVPNITDIDNIKLSHMQLARLLCYTAVVESRNSKPWKAIFNNDTCVLTDSFFNYIMNILNMIKTNQGSLAHNLSVVYHIENIQMNLQNKLKPRSITIEIVDKDKFENKHSDIEVCYVMNNRLHPQDVNSQQTLMCSSFVELNALPFCLYNETLPDDQSMSVFNLYKFEQTKNNSVSKPSRLGNVMFVNSLIDKPITRETIVDIINSYHNACQNLKRSGHRVVGDYRAYERDYKLAALDFIILMLVTSITHRTLKYNMLNIHEKMFQELKTIVCKHSAAKLYDILINYDINKEPLNNFRYNYEVL | ^10^MIINELLFLNDNVNYATNK^28^, ^42^LSAMLLNYK^50^, ^65^NLSFMLENTDK^75^, ^76^IDIIQFDDVK^85^, ^86^NYVQPAIVNLFESHNR^101^, ^141^LLCYTAVVESR^151^, ^184^TNQGSLAHNLSVVYHIENIQMNLQNK^209^, ^229^HSDIEVCYVMNNR^241^, ^301^LGNVMFVNSLIDKPITR^317^, ^318^ETIVDIINSYHNACQNLK^335^, ^400^LYDILINYDINKEPLNNFR^418^ |
| 106 | P24 | 195 | MKTDAQSTSNTHNFMYSPDNNLEVVIITNSDGDHDGYLELTAAAKIMSPFISNGGSTVWTNAAPSHKLIKNNKNYIHVFGLFKYLSNYNLNNKKRPKEYYVLKSIISDLLMGAQGKVFDPLCEVKTQLCAIQESLNEAISTLNVHAAANSPAPDINKLQDMIQDLQSEYNKKITFTTDTILENLKNIKDLMCLNK | ^46^IMSPFISNGGSTVWTNAAPSHK^67^, ^74^NYIHVFGLFK^83^, ^84^YLSNYNLNNK^93^, ^104^SIISDLLMGAQGK^116^, ^126^TQLCAIQESLNEAISTLNVHAAANSPAPDINK^157^, ^158^LQDMIQDLQSEYNK^171^, ^158^LQDMIQDLQSEYNKK^172^, ^173^ITFTTDTILENLK^185^ |
| 26 | Ubiquitin | 77 | MQIFIKTLTGKTITAETEPAETVADLKQKIADKEGVPVDQQRLIFAGKQLEDSKTMADYNIQKESTLHMVLRLRGGY | ^12^TITAETEPAETVADLK^27^, ^30^IADKEGVPVDQQR^42^, ^64^ESTLHMVLR^72^ |
| 75 |  | 259 | MIPLTPLFSRYKDSYLLYSFRLIDLLRASKSAHLTKLLSSQATYLYHFACLMKYKDIQKYEVQQLIEWAINASPDMDLQQFRIEFMDKTTELNLRSCQPKSFTYTFTTIWDTMHFLSLIIDDMVYTRDKSSLDFVMQQLKTMKVLFYNVFFILQCAMCRDHYMNVKGFLIYHIELIEIALDKEKYGTDITFVDSYQQETAGADAAAVSNNMLMKNLMAYVIMTFHNHVNDYKWIQRNQKPPAHYERMTWGEYKKLLNLQ | ^1^MIPLTPLFSR^10^, ^11^YKDSYLLYSFR^21^, ^13^DSYLLYSFR^21^, ^22^LIDLLR^27^, ^37^LLSSQATYLYHFACLMK^53^, ^128^DKSSLDFVMQQLK^140^, ^130^SSLDFVMQQLK^140^, ^215^NLMAYVIMTFHNHVNDYK^232^ |
| 63 | VLF-1 | 379 | MNGFNVRNENNFNSWKIKIQSTPRFESVFDLATDRQRCTPDEVKNNSLWSKYMFPKPFAPTTLKSYKSRFIKIVYCSVDDVHLEDMSYSLDMEFDSIENQTLLIDPQELCRRMLELRSVTKETLQLTINFYTNMMNLPEYKIPRMVMLPRDKELKNIRKKEKNLMLKNVIDTILNFINDKIKMLNGDYVHDRGLIRGAIVFCIMLGTGMRINEARQLSVNDLNVLIKKGKLHSDTINLKRKRSRNNTLNNIKMKPLELAREIYSRNPTILQISKNTSTPFKDFRRLLEESGVEMERPRSNMIRHYLSSNLYNSGVPLQKVAKLMNHESSASTKHYLNKYNIGLDETSSEEENNNNDDDAQHNRNSSGSSGESLLYYRNE | ^8^NENNFNSWK^16^, ^25^FESVFDLATDR^35^, ^52^YMFPKPFAPTTLK^64^, ^168^NVIDTILNFINDK^180^, ^216^QLSVNDLNVLIK^227^, ^286^LLEESGVEMERPR^298^, ^304^HYLSSNLYNSGVPLQK^319^, ^364^NSSGSSGESLLYYR^377^ |
| 88 | VP80 | 692 | MNDSNSLLITRLAAQILSRNIQTVDVIVDDKTLSLEEKIDTLTSMVLAVNGLPQSPPRVSSSDLAASSIKNNSKLVGNDFEMRYNVLRMAVVFVKRYPKYYNETTAGLVAEIENNLLQYQNYVNQGNYQNIQGYDSLLNKAEECYVKIDRLFKESIKKIMDDTEAFEKEQEAERLRAEQTAANALLNRRAQTSADDVVNRADANISTAFSDPLPGSSAPRYMYESSESDTYMEPARHTAEHYTDQDKDYNAAYTADEYNSLVRTVLLRLIEKALATLTNRLHITTIDQLKKFRDYLNSDADAGEFQIFLNQEDCVMLKNLSNLASKFFNVRCVADTLEVMLEALRNNIELVQPESDAVRRIVIKMTQEIKDSTTPLYNIAMYKSDYDAIKNKNIKTLFDLYNDRLPINFLDTSATSPAVRKTPGKRSAEDDLLPTRSNKRANRPEISVISSEDEQEDDDVEDVDYEKESKRRKLEDEDFLKLKALEFSKDIVNEKLQKIIVVTDGMKRLYEYCNCKNSLETLPSAANYGSLLKRLNLYNLDHIEMNVNFYELLFPLTLYNDNDNSDKTLSHQLVNYIFLASNYFQNCAKNFNYMRETFNVFGPFKQIDFMVMFVIKFNFLCDMRNFAKLIDELVPNKQPNMRIHSVLVMRDKIIKLAFSNLQFQTFSKKDKTRNTKHLQRLIMLMNANYNVI | ^201^ADANISTAFSDPLPGSSAPR^220^, ^221^YMYESSESDTYMEPAR^236^, ^248^DYNAAYTADEYNSLVR^263^, ^332^CVADTLEVMLEALR^345^, ^346^NNIELVQPESDAVR^359^, ^371^DSTTPLYNIAMYK^383^, ^405^LPINFLDTSATSPAVR^420^ |
| 4 |  | 340 | MSLAAKLIIYNYYAKYNEVHDVYGESYHHHRIVQEYLSESYVNDMSCIERDVTAMRRLKIGSCTFDEAVKMIDAGDSIKSLSHWFSTGETTGIDDNVRKVLEQIDAVVPVSVRVQTGRQIFSLNNFEREISQDMLGCLQIILGRFEHFMRNGKLLHIANVFNPNSDAVGWWYNKFCVITYVHRIMHRSVPAELVPRLSEAVKKFIRLSKSDYDDRLHVDESYNCPRVIAEMYGRFCGIGKEHFSKHKLSCMHILFQYLRGKTTQEEKSFSCYTVIKDFGRQCIDVYRDLKDVFDLLHAHSMSDKDKNSLMDLLCVMDCEEIDVDCFYYIFESFLNNKHLQ | ^7^LIIYNYYAK^15^, ^16^YNEVHDVYGESYHHHR^31^, ^80^SLSHWFSTGETTGIDDNVR^98^, ^100^VLEQIDAVVPVSVR^113^, ^119^QIFSLNNFER^128^, ^154^LLHIANVFNPNSDAVGWWYNK^174^, ^175^FCVITYVHR^183^, ^268^SFSCYTVIK^276^, ^288^DLKDVFDLLHAHSMSDK^304^ |
| 77 | ODV-E25 | 228 | MWKIVLLIVLLVLIYLYWTNALNLNSLTEASPSLGQSSESVESDENKRLNVKLNNARVANLRIAHGDNKLSQMYIAEKPLSIDDIVKEGSNKVGTNSIFLGTVYDYGVKSPNAASTSSNVTMTRGTANFDIKEFKSMFIVFKGITPTKTVEDNGMLRFEVDSMIVCLIDPNTGPLSEREVRELRKSNCTLVYTKNEAAQQVLLENNFTAINADQTAYLKNYKSYREMN | ^70^LSQMYIAEKPLSIDDIVK^87^, ^158^FEVDSMIVCLIDPNTGPLSER^178^, ^195^NEAAQQVLLENNFTAINADQTAYLK^219^ |
| 120 | ODV-EC27 | 290 | MKRVKCNKVRTVTEIVNSDEKIQKTYELAEFDLKNLSSLESYETLKIKLALSKYMAMLSTLEMTQPLLEIFRNKADTRQIAAVVFSTLAFIHNRFHPLVTNFTNKMEFVVTETNDTSIPGEPILFTENEGVLLCSVDRPSIVKMLSREFDTEALVNFENDNCNVRIAKTFGASKRKNTTRSDDYESNKQPDYDMDLSDFSITEVEATQYLTLLLIVEHAYLHYYIFKNYGVFEYCKSLTDHSLFTNKLRSTMSTKTSNLLLSKFKFTIEDFDKINSNSVTSGFNIYNFNK | ^25^TYELAEFDLK^34^, ^35^NLSSLESYETLK^46^, ^79^QIAAVVFSTLAFIHNR^94^, ^148^EFDTEALVNFENDNCNVR^165^, ^228^NYGVFEYCK^236^, ^266^FTIEDFDK^273^, ^274^INSNSVTSGFNIYNFNK^290^ |
| 10 |  | 356 | MERLLNQLNLGVLPYITTKDIEDRLRDKIVAKAKLAFIKDCFEAVVCENGGLFVLTGGAAVTCHIDDDDYKSALKCIDFDYYGLCSKKMFCNLQTNLQKCVDQHYAELDVLTRQIYMSNPLVMLKCYQNGAYRLNGQIDLHLNRHIKCIKTQYNDEFDLVRFALQIDITSAYGVDEYTDNCVKITTAPLSFNVFFVNVRIMKRPFNADRCIKNFSLLGNEYHVLVSSLQRVLNDQLMCLLKDIFTNKFDYKIKRRLNHLKRLFANLPAESYNSCVNDLTDMYLYKEQNETITNFVKKILDISGPDLGCRKLMRIYLNTDTFRGQLPAYLTHYVNYPHKNLCDQNWKRFMSCIFSLY | ^4^LLNQLNLGVLPYITTK^19^, ^100^CVDQHYAELDVLTR^113^, ^151^TQYNDEFDLVR^161^, ^184^ITTAPLSFNVFFVNVR^199^, ^213^NFSLLGNEYHVLVSSLQR^230^, ^298^ILDISGPDLGCR^309^ |
| 112 | P35 | 299 | MCVIFPVEIDVSQTVIRDCHVDEQTRELVYINKIMNTQLTKPVLMMFNISGPIRSVTRKNNDLRDRIKSKVDEQFDQLEREYSDKIDGFHDNIQYFKDEHYSVSCQNGSVLKSKFAKILKSHDYTDKKSIETYEKYCLPQLVDKHNDCYVAVCVLKPGFENGSNQVLSFEYNPIGNKVIVPFAHEINDTGLYEYDVLAYVDSVEFDGKQFEEFVQKLILPSSFNDSEKVLYYNEASKNKNMIYKALEFTTESSWVKSNKFNWKIFCNGFIYDKKSKALYVKLHNVTSTLNKNVILDMIK | ^34^IMNTQLTKPVLMMFNISGPIR^54^, ^86^IDGFHDNIQYFK^97^, ^136^YCLPQLVDK^144^, ^209^QFEEFVQK^216^, ^217^LILPSSFNDSEK^228^, ^264^IFCNGFIYDK^273^ |
| 72 | VP39 | 350 | MALMPVGMAPRQMRVNRCIFASIVSFDACITYKSPCSPDAYHDDGWFICNSHLIKRFKMSKMVLPIFDEDDNQFKMTIARHLVGNKERGIKRILIPSATNYQEVFNLNSMMQAEQLIFHLIYNNEEAVNVICDNLKYTEGFTSGTQRVIHSVYATTRSILDTTNPNTFCSRVSRDELRFFDVTNARTHRGGVGDQLFNNYSGFLQNLIRRAVAPEYLQIDTEELRFRNSATCIIDETGLVASVPDGPELYNPIRSSDIMKSQPNRLQIRNVLKFEGDTRELDRTLSGYEEYPTYVPLFLGYQIINSENNFLRNDFISRANPNATLGGGVGALAGPAPGVVLGEAGGGVAA | ^34^SPCSPDAYHDDGWFICNSHLIK^55^, ^62^MVLPIFDEDDNQFK^75^, ^190^GGVGDQLFNNYSGFLQNLIR^209^, ^211^AVAPEYLQIDTEELR^225^ |
| 116 | ME53 | 451 | MNRFFRENNIFDAPKTGGKGRVKSLPTPVANSPLSPVRQPPKSNIKPPTRISLPTRTFSANPLERSISSSIVSKKPVVNRKDGYFVPPEFGNKLESLPAYSDKLDFKQERDLRMHFMSDLERNIMKATLKFSTNYIMGYINSKDMRMTGKFASSSVKYKKTTEQHMSDSRCTTCNYRFKDNTRAWFLYVVVHIEKPLDDSDRIDICCQKCYLYHNVPKTSYEIYPSINLVDLSYLARERFFYQYIFPVSLEHTTEVKELKIDDHNCKVFEIIRRIIRNHKEPNERIQTIDLSTTGGLVLRETYTNIVLQRYRSMCTRPDAADDVNCFILQEPSEMMAALQDNRFSGIKGTVFATVKVKKFTEVLDGGAITFPSRPTINNYCKLCKKTKLYYKNPVLYCTKCGFTNVYHFPEYSKLVYYSKAIKNFEMHNEMIIYYDLKMYKKLINIVNNNV | ^114^MHFMSDLER^122^, ^131^FSTNYIMGYINSK^143^, ^219^TSYEIYPSINLVDLSYLAR^237^, ^286^IQTIDLSTTGGLVLR^300^, ^301^ETYTNIVLQR^310^, ^360^FTEVLDGGAITFPSRPTINNYCK^382^ |
| 85 |  | 362 | MSAIALYLEINKLRLKIDEPMQLAIWPQIFPLLCDEHQNVQLNTDVLINFIMHVARKSQNTILNNNAAIASQYAAGNAGVVVAAPASAHPTPRPVINLFARANAVAPAQPSEELINMRRYRNAARKLIHHYSLNSTSSTEYKISDVVMTMIFLLRSEKYHSLFKLLETTFDDYTCRPQMTQVQTDTLLDAVRSLLEMPSTTVDLTTVDIMRSSFARCFNSPIMKYAKIVLLQNVASQRDKRTTLEELLIERGEKIQMLQPQQYINSGTEIPFCDDSEFLNRLLKHIDPYPLSRMYYNAANTMFYTTMENYAVSNCKFNIEDYNNIFKVMENIRKHSNKNLNDQDELNIYLGVQSSNAKRKKY | ^102^ANAVAPAQPSEELINMR^118^, ^127^LIHHYSLNSTSSTEYK^142^, ^165^LLETTFDDYTCRPQMTQVQTDTLLDAVR^190^, ^242^TTLEELLIER^251^, ^328^VMENIRK^334^ |
| 55 | LEF-3 | 385 | MATKRFFSGESSGEPLIKRMAMANSPKKIRENYKRINGKLMGKMTLSIDNEYYYTFRIMSDNKIQEYYGDSQSFKDMEEGKCYDISLNYVKTKFSQMIQINEYKECEMEIETAIPLSAYLTNKHFENEDSVNIIVKYKFIYKKINSNLYKIVFEVAYKNFNDDSDVVQVECFVNAKTLMNLFKNNIKGSDDINEVFKYLKDNENQIFTIYNIKCQQIFNGANVYMNWNVVNSTRIELCEAKENEAYSNLQNCTNARINISRSNKRVASYNVNMLKSELEENDMSDNKFIVQFKSDDLNVADSDDRSTSSDSSKWNKSVFYVNTNKKTEADSLQKLCADFNQISMLLEDNLIKVTIYVTVENGENGNMNVLGLLKYDEDENDYTFL | ^64^IQEYYGDSQSFK^75^, ^64^IQEYYGDSQSFKDMEEGK^81^, ^94^FSQMIQINEYK^104^, ^159^NFNDDSDVVQVECFVNAK^176^, ^201^DNENQIFTIYNIK^213^, ^335^LCADFNQISMLLEDNLIK^352^ |
| 82 | 38K | 320 | MASSLQSKWICLRLNDAIIKRHVLVLSEYADLKYLGFEKYKFFEYVIFQFCNDPHLCKIIENNYNYCMQIFKAPADNMCDIRHNIKRAFKTPVLGHMCVLSNKPPMYSFLKEWFLLPHYKVVSLKSESLTWGFPHVVVFDLDSTLITEEEQIQIRDSFVYDSLQELHEMGCVLVLWSYGSRDHVAHSMRDADLEGYFDIIISEGSTVREERSDLVQNSHNAIVDYNLKKRFIENKFVFDIHNHRSDNNIPKSPKIVIKYLSDKNVNFFKSITLVDDLPTNNYAYDFYVKVKRCPTPVRDWHNYHEQIIDNLIEYDNMYNF | ^22^HVLVLSEYADLK^33^, ^112^EWFLLPHYK^120^, ^212^SDLVQNSHNAIVDYNLK^228^ |
| 40 |  | 319 | MSKRVREMSVVSDETAKRIRQNEHYHAKNESFLGFCNLEEIDYYQCLKMQYVLDQNFDNDFILTVYRMANVVTKQVRPYNSIDEKHHYNTVRNVLILIKNARLVLSNSVKKQYYDDVLNLKKNTDLESYDPLITVFLQIGESINEEIQKLRKALVNFFTNKPDKSDINNPDVVSYQFIFDRVQKLYNREIKQKTKTIIVKRPTTMNRIQIDWKPHSEDEQKMTKQEIAENIVKPCFEQFGTILHIYVCPLNHNRIIVEYANSESVQKAMTVNDDDARFTVTEFSVVQYYNVAKTEMVNQRIDIISKNIEDLKNSLKSYT | ^112^QYYDDVLNLK^121^, ^165^SDINNPDVVSYQFIFDR^181^, ^278^FTVTEFSVVQYYNVAK^293^ |
| 124 | ODV-E56 | 375 | MSFFTNLRRVNKLYPNQASFLADNTRLLTSTPAGFTNVLNAPSVRNLGNNRYQPGYQLSNNRFVSTSDINRITRNNDVPNIRNVFQGISDPQINSLRQLRRMDNVPDFHYHTKQTRSNAVRQNFPETNVRTPEGVQNALQQNPRLHNHMRTLKVAGVGILLAGGGYLLFTASTLVQDIINAINRTGGSYYVQGRNAGENVESCLLLQRTCRQDRNLAQSDVNICSRDPLLANDSPLLTNMCQGFNYETEKTVCRGSNPAANPNSPQYVDISDLPAGQTIMCIEPYSFSDLVGDLGLDWLLGREGLVGKSSNSSDGIRNKIMPIIMMIGAVLFLGLILYFIYRYMTKGGGGGGGSGGAPTPIVIMQHPASTTAPRR | ^27^LLTSTPAGFTNVLNAPSVR^45^, ^83^NVFQGISDPQINSLR^97^, ^131^TPEGVQNALQQNPR^144^, ^195^NAGENVESCLLLQR^208^, ^347^GGGGGGGSGGAPTPIVIMQHPASTTAPR^374^ |
| 43 | VP1054 | 365 | MCSTKKPIKLDLCASVKLTPFKPMRPPKPMQCWIHPRRANCKVTRPRNNYSDPDNENDMLHMTVLNSVFLNEHAKLYYRHLLRNDQAEARKTILNADDVYECVLIKPIRTEHFRSVDEAGEHNMGVLKIIIDTVIKYIGKLADDEYILIADRMYVDLIYSEFRAIILPQSAYIIKGDYAESDSESGQSVDVCNELKYPWNLITANSFIVSTDESRQSQYIYRTFLLYNTVLTAILKQNNPFNVIAENTSISIIVRNLGNCPNNKDRVKCCDLNYGGIPPGHVMCPPREITKKVFHYAKWVRNPNKYKRYSELIARQSEAGGASASLRENVNNQLHARDASQLHLLDWENFMGEFSSYFGLHAHNV | ^141^LADDEYILIADR^152^, ^223^TFLLYNTVLTAILK^236^ |
| 129 |  | 77 | MDSSNCIKIDVKYHMPLHYQCDINADKNVVNAYDAIDVDPNKKFIINHNHEQVDETNKQEVVDKTDATTYNSCIIKI | ^13^YHMPLHYQCDINADK^27^, ^28^NVVNAYDAIDVDPNK^42^, ^44^FIINHNHEQVDETNKQEVVDK^64^ |
| 48 |  | 83 | MLPKYYQNLPYNGKRIFEKFYDRSLQKYKSTHIATKLACCAVRKKYILIDCKWQPRPDANNSDTTTTNTEDSTTDTETEDEIA | ^5^YYQNLPYNGK^14^ |
| 34 |  | 78 | MNTRYATCYVCDELVYLFKKTFSNMSPSAAAFYQQRMAIVKNGIVLCVRCSSKLKITNGISIPIYPNRRAQQHARRSR | ^5^YATCYVCDELVYLFK^19^ |
| 23 | SOD | 151 | MKAICIISGDVHGKIYFQQESANRPLKISGYLLNLPRGLHGFHVHEYGDTSNGCTSAGEHFNPTNEDHGAPDAEIRHVGDLGNIKSVGYNSLTEINMMDNVMSLYGPHNIIGRSLVVHTDKDDLGLTEHPLSKTTGNSDGRLGCGIIAICK | ^15^IYFQQESANRPLK^27^, ^28^ISGYLLNLPR^37^ |
| 3 | PK1 | 275 | MATTTATNATLQTLVQFYENCKNVKTRYKIINGRFGKISILSHKPTSKLYLQKTISAHNFNVDEIKVHQLMNDHPNFIKIYFNHGFINNQVIVMDYIDCPDLFETLQIKGELSHQLVSNIIRQLCEALNDLHKHNFIHNDIKLENVLYFEALDRVYVCDYGLCKHENLPSVHDGTLEYFSPEKIRRHNYARSFDWYAVGVLTYKLLTGGRHPFEKSEDEMLDLNSMKRRQQYNDIGVLKHVRNVNARDFVYCLTRYNLDCRLTNYKQIIKHEFLS | ^143^LENVLYFEALDR^154^ |
| 70 | VP15 | 126 | MNTRGCVNNNKMIFMNALGLQPQSKVKIIAHKTLEKFKRDAYTRFKGVKAIKNELKTYNLTLQQYNEALNQCALNDSRWRDTNNWHHDIKEGVKINKRHIYRVNFNSKTKEIKEYYYIKVECYVNN | ^57^TYNLTLQQYNEALNQCALNDSR^78^ |
| 2 | ORF1629 | 542 | MTNRKYESVQSYLFNNRNNKIDAHQFFERVDTAEAQIIKDSIYDNTVLLNRDVFLNILKFANDVFDNKAYMYVDDSEVSRYYNAVVKMKRLVINVRDPSLRQSLYNTIAYIERLLNIGTVNDSEITMLIADFYDLYSNYNIELPPPPPQALPRSRRPSVVQPAAPAPVPTIVHEQTKPEQIIIPAAPPPPSSVPNIPAPPPPPPPPPSSMSELPPAPPMPTKPQPAAPLDDRQQLLEAIRNEKNRTRLRPVKPKTAPETNTIIEVPTTVLPKEPKPPSASPPPPPPPPAPPAPPPMIDLSSAPLQPPLVDLPAEMLPPPAPSLSNVLSELKSGTVRLKPAQKRPQSEIIPKNSTTKNLIADVLVDTINRRRVAMAKSSSEATSNDEGWDDGGNNRPNAPDVKYVQALFNVFTSSQLYTNDSDEKNTKAHNILNDVESLLQNKTQTNIDKARLLLQDLASRVVLSENPLDSPAIGLQKQPLFETNRNLFYKSIEDLIFKFRYKDAENHLIFALTYHPKDYKFNELLKYVQQLSVNQQRTESNA | ^377^SSSEATSNDEGWDDGGNNRPNAPDVK^402^, ^450^ARLLLQDLASR^460^, ^486^NLFYKSIEDLIFK^498^ |
| 110 | ALK-EXO | 420 | MFASLTSEQKLLLKKYKFNNYVKTIELSRAQLSRWRSNKDIHPKPLDRAEILRVEKATRGQSKNELWTLLRLDRSTASASSNSSGNMLQRPALLFGNAQESHVKETNGIMLDHMREIIKNKITSAVVETVLDCGMFFSSLGLHAASPDAYFSLADGTWIPVEIKCPYNYRDTTVEQMRVELGNGNRKYRVKHTALLVNKKGTPQFEMVKTDAHYKQIQRQMYVMNAPMGFYVVKFKQNLVAVSVPRDETFCNKELFTENNAYVAFAVENSNCERYQCADKRRLSFKMHSCNHNYSGQEIDAMVDRGIYLDYGHLKCAYCNDFSSNSREACDSVLKREHTNCKSFNLKHKNFDNPTYFDYVKRLQSLLKSHHFRNDAKTLAYFGYYLTHTGTLKTFCCGVQNSSPTKHDHLNNCVYYLEIK | ^30^AQLSRWR^36^ |
| 60 |  | 268 | MKINLCNIQFQSLINLLNLQATTIMSLNSNKIKADLIPDENDPKNVTLRLNSVPEECTDDDNFSIDLPLTTPEQKDDFMNAIKPFETLNIESDIIKTEQTDAPATSGDDNNNRKVVDANEDEYTVDGLKLKSKYVAYYKCLKILVDFLVMYVSKETNMKEYEQVYTLGRQLYEVLRSIFVDEPFKLWLERNTHEFDNNTDKILETLQSELKLALADKDKLKTCTFKDIITNFLNTKLDCRYDCADEYIKPNCIVDTYNCCNLVFKKET | ^177^SIFVDEPFK^185^ |
| 22a |  | 54 | MMQMAFMLFLNYTILILSKQSPYSQQRLEKRRRDLTKFDELKGSLSGTLLNQVY | ^20^QSPYSQQRLEK^30^ |
| 111 |  | 70 | MLQSVRVGQFVDNKFVFAFKRSVCNPLHSVQNGHIFIYTSGAVTFCNMSDCVCYLWQRDNGPENYTRVVT | ^1^MLQSVRVGQFVDNK^14^ |
| 91 |  | 105 | MKPTAADIISRVTGGRTGNNIVDIIQANNSPTEGDQLGQFVNRNRSLIKEFVLVVCGFLIFVMIVLFFMLLVVILLNQETVTVQKQKYETTLLENYDIRNRNATT | ^17^TGNNIVDIIQANNSPTEGDQLGQFVNRNR^45^ |
| 117 | EXON0 | 261 | MIRTSSHVLNVQENIMTSNCASSPYSCEATSACAEAQQVMIDNFVFFHMYTADIQIDAKVQCGVRSAAFAIIDDKHLEMYKYRIENKFFYYYDQCADIAKPDRLPDDDGACCHHFIFDAQRIIQCIKEIEGAYGVRDRGNVIVFYPYLKQLRDALKLIKNSFACCFKNINSMQMYVNELISNCLLFIEKLETINKTVKVMNLFVDNSVLYECNVCKEISTDERFLKPKECCEYAICNACCVTMWKTATTHAKCPACRTSYK | ^139^GNVIVFYPYLK^149^ |
| 71 | CG30 | 267 | MEFVKLQCNICFSVAEIKNYFMQPIDRLTIIPVLELDTCKHQLCSMCIRKIRKRKKIPCPLCRVESLHFNVYSINRNVVDVIKCSVTSVAQWNKINDNFDAASLASVLFEKSLLDDAEDSNNAANSDDTMLSESQAILKKLQIDIAEQTQLNIKRQLDLNKLQQTSVFMQEKLDRIKNDYNNMHKSFKELQLKRISTEKALKSLNDDYAKLAAKNARLSNENKVLSNKNIELIKHKNLLQNEYTTLQSYKCITNATTITTNVTINVD | ^95^INDNFDAASLASVLFEK^111^ |
| 30 | P43 | 362 | MDKRANSRKPFLFYNEDYYCEKPKRYFHTNKVIFEKLDPYATNINRCRKLLTDFFDYCLPKYYRRKNKFALLFRLLEPVIKQTGTSSALTAVSDQSRWLEINQFSAWERRDNQYAHKWLIKVAGADMGQQILFIIKQVTKKFKTCNLGFHNYYKLFRRCLSMLLFKHKEVFIKCLQVILKAAMPVKNKGVVKSNYAFAVTNALHYYIVDNPHLLCKDINVAIKVRRLLIKHEMLPTEKRIRLSFEKCSKGIEVPLYEKLLLNHMMRINDDNLQWPSLMNNKKIMEWNANRGFDESNKILHVYIGQYYKSSCRRIKKSFFKYNGWNEQLRFCRTEKFCSLVNLQLNKDGSKKLKRVQRKCDKL | ^122^VAGADMGQQILFIIK^136^ |
| 110a |  | 57 | MSVLDAVEWIANRAFECKDKFIINKLTNADALQHLKFLKVNYNKVVDEHVSTSQYLS | ^21^FIINKLTNADALQHLK^36^ |
| 36 |  | 193 | MLPYEMVISVLVYLSPAQILNLNLPLAYQKSVLFASNSAKVNERIRRLARDNNDDDDSYFYHKQFIKINFLTKKIINVYNKTEMCIRATFDGRYVVTHDILMCFVNKSYVKQLLRGVDTCITLQQLVKMYSPEFGLCVNSKNMFVLTESVLTSISLKHSFGKCEWLDKNIKTVCLQLRKLCINNKQHTTCLPY | ^31^SVLFASNSAK^40^ |
| 29 |  | 217 | MRNAAGLFMIIEPDKAVLLCARRAYRSANAPAADINDTFLEKISIPRGHRDCCDAKVYETAVREFVEETGRFFDSAFIYKFPFTLQWKDDGVTYKYLIYVGVVRGNLIDVNAKPNTYTVKLLPGTFGNDYRIMLKPRRFNCEITRSLAIVPLNKYFNYMNDKQLITYDYSNYIEFFSFVRSIKKRFDNRQLQDFFYATLKKIDNNDAPQKLHALRRV | ^202^IDNNDAPQK^210^ |

**Supplementary Table S6. Host proteins identified in the M3 section of the gel.**

| Protein | Annotation | Size(amino acids) | Sequence | Sequence(s) of corresponding peptide(s) |
| --- | --- | --- | --- | --- |
| BGIBMGA002381-PA | HSC/HSP70 | 649 | MAKAPAVGIDLGTTYSCVGVFQHGKVEIIANDQGNRTTPSYVAFTDTERLIGDAAKNQVAMNPNNTIFDAKRLIGRKFEDATVQADMKHWPFEVVSDGGKPKIKVAYKGEDKTFFPEEVSSMVLTKMKETAEAYLGKTVQNAVITVPAYFNDSQRQATKDAGTISGLNVLRIINEPTAAAIAYGLDKKGTGERNVLIFDLGGGTFDVSILTIEDGIFEVKSTAGDTHLGGEDFDNRMVNHFVQEFKRKYKKDLATNKRALRRLRTACERAKRTLSSSTQASIEIDSLFEGIDFYTSITRARFEELNADLFRSTMEPVEKSLRDAKMDKAQIHDIVLVGGSTRIPKVQKLLQDFFNGKELNKSINPDEAVAYGAAVQAAILHGDKSEEVQDLLLLDVTPLSLGIETAGGVMTTLIKRNTTIPTKQTQTFTTYSDNQPGVLIQVFEGERAMTKDNNLLGKFELTGIPPAPRGVPQIEVTFDIDANGILNVSAIEKSTNKENKITITNDKGRLSKEEIERMVNEAEKYRNEDDKQKETIQAKNALESYCFSMKSTMEDEKLKEKISDSDKQTILDKCNDTIKWLDSNQLADKEEYEHKQKELEGIYNPIITKMYQGAGGVPGGMPGFPGGAPGAGGAAPGAGGAGPTIEEVD | ^37^TTPSYVAFTDTER^49^, ^138^TVQNAVITVPAYFNDSQR^155^,  ^160^DAGTISGLNVLR^171^, ^172^IINEPTAAAIAYGLDK^187^, ^302^FEELNADLFR^311^, ^329^AQIHDIVLVGGSTR^342^, ^362^SINPDEAVAYGAAVQAAILHGDK^384^, ^459^FELTGIPPAPR^469^ |
| BGIBMGA011746-PA | DEAD/DEAH box helicase | 569 | MRIDNNGNVFVTSGVLSASAKQCIDFFLCTRSGNWNNSRGNGGSKFGGGNKFGDNSSRFGNGGNRFGGSGGFGGKKEFSGGQNMRRPDWDSVSLQPFNKNFYDPHPTVLKRSPYEVEEYRNNHEVTVSGVEVHNPIQYFEEANFPDYVQQGVKTMGYKEPTPIQAQGWPIAMSGKNLVGVAQTGSGKTLAYILPAIVHINNQPPIRRGDGPIALVLAPTRELAQQIQQVAADFGHTSYVRNTCVFGGAPKREQARDLERGVEIVIATPGRLIDFLEKGTTNLQRCTYLVLDEADRMLDMGFEPQIRKIIEQIRPDRQTLMWSATWPKEVKKLAEDYLGDYIQINIGSLQLSANHNILQIVDICQEHEKENKLNVLLQEIGQSQEPGAKTIIFVETKRKAENISRNIRRYGWPAVCMHGDKTQQERDEVLYQFKEGRASILVATDVAARGLDVDGIKYVINFDYPNSSEDYIHRIGRTGRSKSKGTSYAFFTPSNSRQAKDLVSVLQEANQIISPQLQSMADRCGGGGGGWNRNRFGGGGRGGGGSFKRGSNFGKSSQRGGSGYKRFDDY | ^112^SPYEVEEYR^120^, ^159^EPTPIQAQGWPIAMSGK^175^, ^188^TLAYILPAIVHINNQPPIR^206^, ^208^GDGPIALVLAPTR^220^, ^221^ELAQQIQQVAADFGHTSYVR^240^, ^260^GVEIVIATPGR^270^, ^285^CTYLVLDEADR^295^, ^317^QTLMWSATWPK^327^, ^437^ASILVATDVAAR^448^, ^457^YVINFDYPNSSEDYIHR^473^, ^484^GTSYAFFTPSNSR^496^ |
| BGIBMGA010673-PA | DEAD/DEAH box helicase | 372 | MGIFEKGWEKPSPIQEASIPIALSGKDVLARAKNGTGKTGAYCIPVLEQVDPKKDTIQALIVVPTRELALQTSQICIELAKHTDIRVMVTTGGTNLRDDIMRIYQNVQVIIATPGRMIDLMDKQVAKMDQCRMLVLDEADKLLSQDFKGMLDMVISRLPKERQILLFSATFPLSVKQFMEKHLKEPYEINLMEELTLKGVTQYYAFVQERQKVHCLNTLFSKLQINQSIIFCNSTQRVELLAKKITELGYCCYYIHARMAQAHRNRVFHDFRAGLCRNLVCSDLFTRGIDVQAVNVVINFDFPRMAETYLHRIGRSGRFGHLGIAINLITYDDRFALHRIEQELGTEIKPIPKVIDPALYVARPEDEDLGDK | ^39^TGAYCIPVLEQVDPK^53^, ^54^KDTIQALIVVPTR^66^, ^103^IYQNVQVIIATPGR^116^, ^163^QILLFSATFPLSVK^176^, ^199^GVTQYYAFVQER^210^, ^245^ITELGYCCYYIHAR^258^, ^278^NLVCSDLFTR^287^, ^340^IEQELGTEIKPIPK^353^, ^354^VIDPALYVARPEDEDLGDK^372^ |
| BGIBMGA006405-PA | RNA recognition motif | 361 | MRPQNGDDGYEEPEVHRKIFIGGLNYKTTDESLKTHFVKWGEIVDVVVMKDPKTKRSRGFGFITYSKAHMVDDAQNNRPHKIDGRFVETKRAVARQDIKNPEAGATVKKLFIGGIKDDHEEEELRQYFSNYGNVQNVSIVTDKNTGKKRGFGFVEFDDYDPVDKVCLNGPHKINGKPLDVKKALPKDGSDQRGGGGGGGRQGGGSGRNDSWGSSGGYGNNQGGWGGNSNPWESNQGGWGNQGGGGGYGGGGNGRGGWGNQDFGNYNQQSYSGGPTRNQAYGNNRSTPYNMNQGAGGGGGGGYGGDSFDNGITGAISSSSEKLAIAGKLPLKPSCYSEISHPTGTLTGSSRTDYPREHKWLI | ^1^MRPQNGDDGYEEPEVHR^17^, ^40^WGEIVDVVVMK^50^, ^40^WGEIVDVVVMKDPK^53^, ^126^QYFSNYGNVQNVSIVTDK^143^, ^150^GFGFVEFDDYDPVDK^164^ |
| BGIBMGA014155-PA | K Homology (KH) domain | 377 | MADKYDKNGYNSGDFKRNTTQGKPEELDQNGEEGIKINEKAGEYMRELLSEKIKLNNSKFPLSMKLIDQEVTKVQASGRITKDSKYVDVFRDKPTKVTVKVLVPIKEHPKFNFVGKLLGPKGNTMKQLQEDTLCKMAVLGRGSMRDRQKEEELRQSLDPKYSHLADDLHVEISAIAPPAEAHARIAYALAEVKKYLVPDTNETVWMSQMRDIDVRDVHPARQPLLANPAKSILYRNTGAERTHPYREPAPVMHPPAAMPPRSMPPKNKIISILERARTAMEGSYSYEDHYAPPEPPMRGGGRAAEPDVYYERGHERFYEDEAHYREEPREYKASSRDVGTRRPAPHPPAHRHYPRAAPYSRLRRLKEDKTKYDKKRA | ^18^NTTQGKPEELDQNGEEGIK^36^, ^161^YSHLADDLHVEISAIAPPAEAHAR^184^, ^195^YLVPDTNETVWMSQMR^210^, ^247^EPAPVMHPPAAMPPR^261^, ^278^TAMEGSYSYEDHYAPPEPPMR^298^, ^317^FYEDEAHYREEPR^329^ |
| BGIBMGA011965-PA | DEAD/DEAH box helicase | 628 | MTIDVVAVVEEVGVAEGEDPAAVVGVATGRKSADLVDLAEVVVGTVDAAVGMAAVEDETAAGVLGEWGIGMIAPISKMISLAKNFYVPHPNVQRRTQSEIEAYRSQHQITVKGRDVPAPSIFFEEGGFPDYAMKEILKQGFPNPTPIQAQGWPIALSGRDMVGIAQTGSGKTLAYILPAIVHIINQPRLLRDDGPIVLVLAPTRELAQQIQQVANEFGQSIHVRNTCIFGGAPKGPQGRCLERGVEIVIATPGRLIDFLEKETTNLRRCTYLVLDEADRMLDMGFEPQIRKIIEQIRPDRQVLMWSATWPREVQNLAEEFLHDYIQINIGSLELSANHNILQIVDVCEEWEKNDKLITLLTEISSEEETKTIIFAETKRRVDDICKAINRAGWRALAIHGDKNQQDRDYVLNQFRHHAAAILVATDVAARGLDVEDVKFVINYDYPNNSEDYVHRIGRTGRSQNTGTAYTLFTPNNSAKARDLMSVLQEANQVVNPKLLELAQCGMGFKGKFGRSRYRDDGGRGRDGGSRGGRGSRGGGRGGYGDRGPKDCAGRYDAVRSCSGKLVTLIQLRLKIMFRGGMQVVRRGLVNTLHQMSRELISQSKKFLGACAGHNIDDGPASSFNCGAT | ^115^DVPAPSIFFEEGGFPDYAMK^134^, ^139^QGFPNPTPIQAQGWPIALSGR^159^, ^172^TLAYILPAIVHIINQPR^188^, ^192^DDGPIVLVLAPTR^204^, ^205^ELAQQIQQVANEFGQSIHVR^224^, ^244^GVEIVIATPGR^254^, ^269^CTYLVLDEADR^279^, ^439^FVINYDYPNNSEDYVHR^455^ |
| BGIBMGA011581-PA | SUMO | 91 | MADEKKGENEHINLKVLGQDNAIVQFKIKKHTPLRKLMNAYCDRAGLSMQVVRFRFDGQPINENDTPTSLEMEEGDTIEVYQQQTGGVSLV | ^16^VLGQDNAIVQFK^27^ |
| BGIBMGA004881-PA | RNA recognition motif | 252 | MVPQTKVFVGSLPQGSKPEDLRKLFERFGVVTECDIMNRCGFVHMQTEEQAAAAIRALHNSTFNGGVISVERGRIKERGQRGGGRGGRGSMRGGMERRGGGPVRHAPPPRDAPYMRDRPGPVRGYERGPPAAPYDERYAYGEDRRGFALMDRGPRDAYAPPAPAYPDERYQNHISVVEGTRRYDEPPYDDRHYDERAQYERPRPAHHIPNGDMYSRRSPAPMYSGRGGPAPVAGGYERDHYVPQYMQPPLGR | ^7^VFVGSLPQGSKPEDLR^22^, ^28^FGVVTECDIMNR^39^, ^57^ALHNSTFNGGVISVER^72^, ^156^DAYAPPAPAYPDER^169^ |
| BGIBMGA013464-PA | RNA recognition motif | 287 | MANNDNFAQDITTDNQLNGNAENGGGDSQDHNSAEAPGRDDDRKLFVGGLSWETTDKELRDHFGAYGEIESINVKTDPNTGRSRGFAFIVFKAPESIDKVMAAGEHTINNKKVDPKKAKARHGKIFVGGLSSEISDDEIRNFFSEFGTILEVEMPFDKTKNQRKGFCFITFESEQVVNDLLKTPKRTIGGKEVDVKRATPKPDGPGGIGTRGGRGGRGARGGRGGRGGYGGQPWGNQGYGGYGYGQGGYGGGYDSYGYGGYGYDGYGGYGGYDYSGYPNYGKRGKQY | ^45^LFVGGLSWETTDK^57^, ^61^DHFGAYGEIESINVK^75^, ^125^IFVGGLSSEISDDEIR^140^ |
| BGIBMGA004822-PA | DEAD/DEAH box helicase | 405 | MTSSEVSSNRKILSEDLSNVEFDTSEDVEVIPTFDSMGLRDELLRGIYTYGFEKPSAIQQRSILPIVKGRDVIAQAQSGTGKTATFSISILQTLDTTLRETQVLILSPTRELATQIQKVILALGDFMNVQCHACIGGTNLGEDIRKLDYGQHVVSGTPGRVFDMIRRRVLRTRSIKMLVLDEADEMLNKGFKEQIYDVYRYLPPATQVVLISATLPHEILEMTSKFMTDPIRILVKRDELTLEGIKQFFVAVEREEWKFDTLCDLYDTLTITQAVIFCNTKRKVDWLTQKMQEANFTVSSMHGDMPQKERDNIMKEFRSGQSRVLITTDVWARGIDVQQVSLVINYDLPNNRELYIHRIGRSGRFGRKGVAINFVKSDDIRILRDIEQYYSTQIDEMPMNVADLI | ^46^GIYTYGFEKPSAIQQR^61^, ^100^ETQVLILSPTR^110^, ^201^YLPPATQVVLISATLPHEILEMTSK^225^, ^324^VLITTDVWAR^333^ |
| BGIBMGA007410-PA | RNA recognition motif | 314 | MPGTGTFKIFIGNLSDKTTEADLRPLFEKYGTVVECDIVRNYGFVHMENEQVGREAIQNLNGELVHGQAIKIEAAKSRKAPSTPTTKIFVGNLTDKTRAPEVRELFQKFGTVVECDIVRNYGFVHLDATGDVNDAIKELNGMMVDGQPMKVQLSTSRVRQRPGMGDPEQCYRCGRGGHWSKECPKALGPDRNGFRDRAFGRDPYPPPPPPPFLRDRMMGGFGDPYDGYYDRSRFDSPRDLFERRYPVGGASRGLELGGSRGARGDFVSPPLRREPMPPMPNLPPLRSGMGSMRSSYDPMYSRRSPPPGPQMSRG | ^41^NYGFVHMENEQVGR^54^, ^55^EAIQNLNGELVHGQAIK^71^, ^120^NYGFVHLDATGDVNDAIK^137^ |
| BGIBMGA001549-PA | Ubiquitin | 153 | MQIFVKTLTGKTITLEVEPSDTIENVKAKIQDKEGIPPDQQRLIFAGKQLEDGRTLSDYNIQKESTLHLVLRLRGGMQIFVKTLTGKTITLEVEASDTIENVKAKIQDKEGIPPDQQRLIFAGKQLEDGRTLSDYNIQKESTLHLVLRLRGGM | ^12^TITLEVEPSDTIENVK^27^, ^64^ESTLHLVLR^72^, ^88^TITLEVEASDTIENVK^103^ |
| BGIBMGA006196-PA | Chaperone DnaJ(Type I HSP40) | 313 | MGGSRGCGQGRGPVRGEDTMHPLAVTLEDLYAGKTTKLQLSKNVICAHCKGVGGKPGSLISCKDCRGQGIKVSYQQIAPHMTRQFHSRCPSCLGQGETFNEKDKCSKCKGKKVLNETKILEVHIEKGMRDNQKIYFRGEGDQQPDTEPGDVIIVLQQKPHEKFKRNGDNLIMKHEITLTEALCGFEFVAKHLDGRDLLIRHLPGEVIKPGDVKCVQGEGMPIYKNLFEKGNFYVKFDVVFPENHFANEEQLKQIETILPPRPAFVMPTGEDVEEVNLMDYFTPSESSRGREEAYASDDEEHMHAGPGVQCAHQ | ^72^VSYQQIAPHMTR^83^, ^89^CPSCLGQGETFNEK^102^, ^236^FDVVFPENHFANEEQLK^252^ |

**Supplementary Table S7. Viral proteins identified in the M4 section of the gel.**

| ORF | Protein | Size(amino acids) | Sequence | Sequence(s) of corresponding peptide(s) |
| --- | --- | --- | --- | --- |
| 1 | polyhedrin | 245 | MPNYSYTPTIGRTYVYDNKYYKNLGCLIKNAKRKKHLVEHEQEEKQWDLLDNYMVAEDPFLGPGKNQKLTLFKEIRSVKPDTMKLIVNWSGKEFLRETWTRFVEDSFPIVNDQEVMDVYLVANLKPTRPNRCYKFLAQHALRWEEDYVPHEVIRIVEPSYVGMNNEYRISLAKKGGGCPIMNIHSEYTNSFESFVNRVIWENFYKPIVYIGTDSAEEEEILIEVSLVFKIKEFAPDAPLFTGPAY | ^1^MPNYSYTPTIGR^12^, ^13^TYVYDNK^19^, ^13^TYVYDNKYYK^22^, ^20^YYKNLGCLIK^29^, ^23^NLGCLIKNAK^32^, ^36^HLVEHEQEEK^45^, ^36^HLVEHEQEEKQWDLLDNYMVAEDPFLGPGK^65^, ^46^QWDLLDNYMVAEDPFLGPGK^65^, ^46^QWDLLDNYMVAEDPFLGPGKNQK^68^, ^69^LTLFKEIR^76^, ^77^SVKPDTMKLIVNWSGK^92^, ^85^LIVNWSGK^92^, ^85^LIVNWSGKEFLR^96^, ^93^EFLRETWTR^101^, ^102^FVEDSFPIVNDQEVMDVYLVANLKPTRPNR^131^, ^135^FLAQHALR^142^, ^135^FLAQHALRWEEDYVPHEVIR^154^, ^143^WEEDYVPHEVIR^154^, ^155^IVEPSYVGMNNEYR^168^, ^155^IVEPSYVGMNNEYRISLAK^173^, ^174^KGGGCPIMNIHSEYTNSFESFVNR^197^, ^175^GGGCPIMNIHSEYTNSFESFVNR^197^, ^198^VIWENFYKPIVYIGTDSAEEEEILIEVSLVFK^229^, ^230^IKEFAPDAPLFTGPAY^245^, ^232^EFAPDAPLFTGPAY^245^ |
| 75 |  | 259 | MIPLTPLFSRYKDSYLLYSFRLIDLLRASKSAHLTKLLSSQATYLYHFACLMKYKDIQKYEVQQLIEWAINASPDMDLQQFRIEFMDKTTELNLRSCQPKSFTYTFTTIWDTMHFLSLIIDDMVYTRDKSSLDFVMQQLKTMKVLFYNVFFILQCAMCRDHYMNVKGFLIYHIELIEIALDKEKYGTDITFVDSYQQETAGADAAAVSNNMLMKNLMAYVIMTFHNHVNDYKWIQRNQKPPAHYERMTWGEYKKLLNLQ | ^1^MIPLTPLFSR^10^, ^11^YKDSYLLYSFR^21^, ^13^DSYLLYSFR^21^, ^22^LIDLLR^27^, ^37^LLSSQATYLYHFACLMK^53^, ^56^DIQKYEVQQLIEWAINASPDMDLQQFR^82^, ^60^YEVQQLIEWAINASPDMDLQQFR^82^, ^101^SFTYTFTTIWDTMHFLSLIIDDMVYTR^127^, ^128^DKSSLDFVMQQLK^140^, ^130^SSLDFVMQQLK^140^, ^144^VLFYNVFFILQCAMCR^159^, ^167^GFLIYHIELIEIALDK^182^, ^167^GFLIYHIELIEIALDKEK^184^, ^185^YGTDITFVDSYQQETAGADAAAVSNNMLMK^214^, ^215^NLMAYVIMTFHNHVNDYK^232^, ^247^MTWGEYK^253^ |
| 134 |  | 109 | MYSTSKINNARVVASQHDYDRDQIKRELNSLRRNVHDMCTRSGTSFDCNKFLRSDDMTPVVTTITPKRTADYKITEYVSDVKTIKPSNRPLVESGPLVQEAAKYGRCTV | ^54^SDDMTPVVTTITPK^67^, ^74^ITEYVSDVK^82^, ^83^TIKPSNRPLVESGPLVQEAAK^103^ |
| 108 | PP34 | 315 | MKPTNNVMFDDASVLWIDTDYIYQNLKMPLQAFQQLLFTIPSKHRKMINDAGGSCHNTVKYMVDIYGASVLILRTPCSFADQLLSTFIANNYLCYFYRRRRSRSRSRSRSPHGRPRSRSRSRSRSRSRSSSPRQGRRQIFDALEKIRHQNDMLMNNVNQINLNQTNQFLELSNVMTGVRNQNVQLLAALETAKDVILTRLNTLLSEITDSLPDLTLMLDKLAEQLLEAINTMQQTQRNELNNTNSILTNLASSITNINGTLNNLLTAIENLAGGGGGGGGGGGGNFNEADRQKLDLVHTLVNDIKNILTGTLTTK | ^1^MKPTNNVMFDDASVLWIDTDYIYQNLK^27^, ^180^NQNVQLLAALETAK^193^, ^200^LNTLLSEITDSLPDLTLMLDK^220^ |
| 106 | P24 | 195 | MKTDAQSTSNTHNFMYSPDNNLEVVIITNSDGDHDGYLELTAAAKIMSPFISNGGSTVWTNAAPSHKLIKNNKNYIHVFGLFKYLSNYNLNNKKRPKEYYVLKSIISDLLMGAQGKVFDPLCEVKTQLCAIQESLNEAISTLNVHAAANSPAPDINKLQDMIQDLQSEYNKKITFTTDTILENLKNIKDLMCLNK | ^117^VFDPLCEVK^125^, ^173^ITFTTDTILENLK^185^ |
| 66 | GP41 | 403 | MTDERGNFYYNTPPPPLRYPSNPATAIFTNAQTYNNAPGYVPPTTRDNKMDTSRSNSTNSVAIAPYNKSKEPTLDAGESIWYNKCVDFVQKIIRYYRCNDMSELSPLMIHFINTIRDMCIDTNPINVNVVKRFESEETMIRHLIRLQKELGQGNAAESLPSDSNIFQASFVLNSLPAYAQKFYNGGADMLGKDALAEAAKQLSLAVQYMVAESVTCNIPIPLPFNQQLANNYMTLLLKHATLPPNIQSAVESRRFPHINMINDLINAVIDDLFAGGGDYYHYVLNEKNRARIMSLKENVAFLAPLSASANIFNYMAELATRAGKQPSMFQNATFLTSAANAVNSPAAHLTKNACQDSLTELAFQNETLRRFIFQQINYNKDANAIIAAAAPNVTRPNTKGRTV | ^297^ENVAFLAPLSASANIFNYMAELATR^321^ |
| 68 |  | 181 | MANTSNITPDIIVNAQINSEDENVLDFIIEDEYYLKKRGVGAHIIKVASSQQLRLLYKNAYSAVSCGNYSILCNLVQNGEYDLNAIMFNCAEIKLNKGQMLFQTKIYRPDNNKTDAAVNTSSPKRAVETENDDDDDEDDAASAIDEQKENTDAVGIDFEENIDDGDVSAPKKQKLDNAEQN | ^47^VASSQQLR^54^ |
| 51 |  | 155 | MYNKFLIYLHLNGLHGEAKYYKYLMSQMDFENQVADEIRRFCETRLKPAISCNTLTAENLNTLVDSVVCKNGLLNPYAKEVQFALQYFFDDDEISKQDQDDFKLFLLHNYDNCENIEEYFLINNFSIADYEFEDMFEIVRIDCKDLLLLLAKYNM | ^71^NGLLNPYAK^79^ |
| 81 | BRO-c | 318 | MAQVKIGEFKFGEDTFTLRYVLGDEQPVRFVAKDIASSLKYVNCERAIRVHVDGKYKSTFEHADQIQHHAPDSVAKQGDPLYLHPHTVLITKSGVIQLIMKSKLPYAIELQEWLLEEVIPQVLCTGKYAPAVEMDTNDVIAKIDDLTQKLTVANADLAEANRSLILFANEMIVARRDAETARQDCENARRETAQLANRMADIAQDVIAKPSNPQLCHSLAVCDVGNNEFAFLRPQKRSLGRSLKRLGSNDVIFSSDYVPNSMNVLNKVKEAIPRNKFKAKHNRITLLEDYTREELMNVIGSTMTDRQIARMNSLRNAQ | ^307^QIARMNSLR^315^ |
| 55 | LEF-3 | 385 | MATKRFFSGESSGEPLIKRMAMANSPKKIRENYKRINGKLMGKMTLSIDNEYYYTFRIMSDNKIQEYYGDSQSFKDMEEGKCYDISLNYVKTKFSQMIQINEYKECEMEIETAIPLSAYLTNKHFENEDSVNIIVKYKFIYKKINSNLYKIVFEVAYKNFNDDSDVVQVECFVNAKTLMNLFKNNIKGSDDINEVFKYLKDNENQIFTIYNIKCQQIFNGANVYMNWNVVNSTRIELCEAKENEAYSNLQNCTNARINISRSNKRVASYNVNMLKSELEENDMSDNKFIVQFKSDDLNVADSDDRSTSSDSSKWNKSVFYVNTNKKTEADSLQKLCADFNQISMLLEDNLIKVTIYVTVENGENGNMNVLGLLKYDEDENDYTFL | ^201^DNENQIFTIYNIK^213^ |
| 109 |  | 220 | MSDKTPTKKSGGHAMTLRERGVTKPPKKSEKLQQYKKAIAAEQTLRTTADVSSLQNHGESAVFQELERLENVVVVLENEQKRLYPILNTPLDNFIVAFVNPTYPMAYFVNTDYKLKLECARITSDLLYKNKNEVAINRPKISSFKLQLNDVILDTIETIEYDLQNKVLTITAPVQDQELRKSIIYFNILNSSDSWEVPKYMKNLFDEMQLEPPAILPLGL | ^38^AIAAEQTLR^46^ |
| 5 |  | 331 | MLSWLWNWWMWSGDNDDDDNDAAIAAEDRFDPDDYKKYHINVQQWSHIVKWDSFKCNTHSFKYRYVHNDTNAKFYNVIDFCKGLEIAHDDILDCNWDSDQVYHLNEIIFHKQKSKRDLNSLGALFATKQGLLKILMRLNFDNKSNALLHLQTEGERDDLRDKIESVLKHVKKLNTNSEKFMVTHETFKNDVGNRFEQFELRLNELDAKLNMLQSAEKLKTAIVTESKNGTVTFPRDITKHQHLAIFSERIDDRIKLAFVLGQERHFRKRKMRFEDDMEVLYDGVHPNPLLAIQCINEKLYDKHYKIRKIAKRVIDVDCTHNVVKEVIQEVL | ^228^NGTVTFPR^235^ |

**Supplementary Table S8. Viral proteins identified in the M5 section of the gel.**

| ORF | Protein | Size(amino acids) | Sequence | Sequence(s) of corresponding peptide(s) |
| --- | --- | --- | --- | --- |
| 1 | polyhedrin | 245 | MPNYSYTPTIGRTYVYDNKYYKNLGCLIKNAKRKKHLVEHEQEEKQWDLLDNYMVAEDPFLGPGKNQKLTLFKEIRSVKPDTMKLIVNWSGKEFLRETWTRFVEDSFPIVNDQEVMDVYLVANLKPTRPNRCYKFLAQHALRWEEDYVPHEVIRIVEPSYVGMNNEYRISLAKKGGGCPIMNIHSEYTNSFESFVNRVIWENFYKPIVYIGTDSAEEEEILIEVSLVFKIKEFAPDAPLFTGPAY | ^1^MPNYSYTPTIGR^12^, ^13^TYVYDNKYYK^22^,  ^20^YYKNLGCLIK^29^, ^36^HLVEHEQEEK^45^,  ^36^HLVEHEQEEKQWDLLDNYMVAEDPFLGPGK^65, 46^QWDLLDNYMVAEDPFLGPGK^65^, ^46^QWDLLDNYMVAEDPFLGPGKNQK^68^, ^69^LTLFKEIR^76^, ^77^SVKPDTMKLIVNWSGK^92^, ^85^LIVNWSGK^92^, ^85^LIVNWSGKEFLR^96^, ^93^EFLRETWTR^101^,  ^102^FVEDSFPIVNDQEVMDVYLVANLKPTRPNR^131^, ^135^FLAQHALR^142^, ^135^FLAQHALRWEEDYVPHEVIR^154^  ^143^WEEDYVPHEVIR^154^, ^155^IVEPSYVGMNNEYRISLAK^173^, ^155^IVEPSYVGMNNEYR^168^, ^174^KGGGCPIMNIHSEYTNSFESFVNR^197^,^175^GGGCPIMNIHSEYTNSFESFVNR^197^, ^198^VIWENFYKPIVYIGTDSAEEEEILIEVSLVFK^229^, ^230^IKEFAPDAPLFTGPAY^245^, ^232^EFAPDAPLFTGPAY^245^ |
| 106 | P24 | 195 | MKTDAQSTSNTHNFMYSPDNNLEVVIITNSDGDHDGYLELTAAAKIMSPFISNGGSTVWTNAAPSHKLIKNNKNYIHVFGLFKYLSNYNLNNKKRPKEYYVLKSIISDLLMGAQGKVFDPLCEVKTQLCAIQESLNEAISTLNVHAAANSPAPDINKLQDMIQDLQSEYNKKITFTTDTILENLKNIKDLMCLNK | ^46^IMSPFISNGGSTVWTNAAPSHK^67^, ^71^NNKNYIHVFGLFK^83^, ^74^NYIHVFGLFK^83^, ^84^YLSNYNLNNK^93^, ^98^EYYVLK^103^, ^98^EYYVLKSIISDLLMGAQGK^116^, ^104^SIISDLLMGAQGK^116^, ^117^VFDPLCEVK^125^, ^126^TQLCAIQESLNEAISTLNVHAAANSPAPDINK^157^, ^158^LQDMIQDLQSEYNK^171^, ^158^LQDMIQDLQSEYNKK^172^, ^172^KITFTTDTILENLK^185^, ^173^ITFTTDTILENLK^185^, ^173^ITFTTDTILENLKNIK^188^, ^186^NIKDLMCLNK^195^ |
| 134 |  | 109 | MYSTSKINNARVVASQHDYDRDQIKRELNSLRRNVHDMCTRSGTSFDCNKFLRSDDMTPVVTTITPKRTADYKITEYVSDVKTIKPSNRPLVESGPLVQEAAKYGRCTV | ^12^VVASQHDYDRDQIK^25^, ^42^SGTSFDCNKFLR^53^, ^51^FLRSDDMTPVVTTITPK^67^, ^54^SDDMTPVVTTITPK^67^, ^54^SDDMTPVVTTITPKR^68^, ^69^TADYKITEYVSDVK^82^, ^74^ITEYVSDVK^82^, ^74^ITEYVSDVKTIKPSNRPLVESGPLVQEAAK^103^, ^83^TIKPSNRPLVESGPLVQEAAK^103^, ^83^TIKPSNRPLVESGPLVQEAAKYGR^106^ |
| 77 | ODV-E25 | 228 | MWKIVLLIVLLVLIYLYWTNALNLNSLTEASPSLGQSSESVESDENKRLNVKLNNARVANLRIAHGDNKLSQMYIAEKPLSIDDIVKEGSNKVGTNSIFLGTVYDYGVKSPNAASTSSNVTMTRGTANFDIKEFKSMFIVFKGITPTKTVEDNGMLRFEVDSMIVCLIDPNTGPLSEREVRELRKSNCTLVYTKNEAAQQVLLENNFTAINADQTAYLKNYKSYREMN | ^63^IAHGDNKLSQMYIAEKPLSIDDIVK^87^  ^70^LSQMYIAEKPLSIDDIVK^87^  ^93^VGTNSIFLGTVYDYGVK^109^  ^110^SPNAASTSSNVTMTR^124^, ^125^GTANFDIKEFK^135^, ^136^SMFIVFKGITPTK^148^  ^158^FEVDSMIVCLIDPNTGPLSER^178^, ^158^FEVDSMIVCLIDPNTGPLSEREVR^181^, ^186^SNCTLVYTKNEAAQQVLLENNFTAINADQTAYLK^219^, ^195^NEAAQQVLLENNFTAINADQTAYLK^219^ |
| 108 | PP34 | 315 | MKPTNNVMFDDASVLWIDTDYIYQNLKMPLQAFQQLLFTIPSKHRKMINDAGGSCHNTVKYMVDIYGASVLILRTPCSFADQLLSTFIANNYLCYFYRRRRSRSRSRSRSPHGRPRSRSRSRSRSRSRSSSPRQGRRQIFDALEKIRHQNDMLMNNVNQINLNQTNQFLELSNVMTGVRNQNVQLLAALETAKDVILTRLNTLLSEITDSLPDLTLMLDKLAEQLLEAINTMQQTQRNELNNTNSILTNLASSITNINGTLNNLLTAIENLAGGGGGGGGGGGGNFNEADRQKLDLVHTLVNDIKNILTGTLTTK | ^28^MPLQAFQQLLFTIPSK^43^, ^148^HQNDMLMNNVNQINLNQTNQFLELSNVMTGVR^179^, ^180^NQNVQLLAALETAK^193^, ^180^NQNVQLLAALETAKDVILTR^199^, ^292^QKLDLVHTLVNDIK^305^ |
| 17 |  | 129 | MDGSVVSMCVDNAFAYTTDDLLKNISFSHSKCAPFKLQNYTVLKRLSNGFIDKSVDVGSISELQKFNFKINRLTSYISNIFEYEFVVLEHNLSTVHVINAETKTKLGHINVSLNQNDPNVLILTVTLTS | ^37^LQNYTVLK^44^, ^37^LQNYTVLKR^45^, ^45^RLSNGFIDK^53^, ^46^LSNGFIDK^53^, ^46^LSNGFIDKSVDVGSISELQK^65^, ^54^SVDVGSISELQK^65^, ^54^SVDVGSISELQKFNFK^69^ |
| 75 |  | 259 | MIPLTPLFSRYKDSYLLYSFRLIDLLRASKSAHLTKLLSSQATYLYHFACLMKYKDIQKYEVQQLIEWAINASPDMDLQQFRIEFMDKTTELNLRSCQPKSFTYTFTTIWDTMHFLSLIIDDMVYTRDKSSLDFVMQQLKTMKVLFYNVFFILQCAMCRDHYMNVKGFLIYHIELIEIALDKEKYGTDITFVDSYQQETAGADAAAVSNNMLMKNLMAYVIMTFHNHVNDYKWIQRNQKPPAHYERMTWGEYKKLLNLQ | ^1^MIPLTPLFSR^10^, ^11^YKDSYLLYSFR^21^, ^13^DSYLLYSFR^21^, ^22^LIDLLR^27^, ^37^LLSSQATYLYHFACLMK^53^, ^60^YEVQQLIEWAINASPDMDLQQFR^82^, ^101^SFTYTFTTIWDTMHFLSLIIDDMVYTR^127^, ^128^DKSSLDFVMQQLK^140^, ^130^SSLDFVMQQLK^140^, ^144^VLFYNVFFILQCAMCR^159^, ^167^GFLIYHIELIEIALDK^182^, ^167^GFLIYHIELIEIALDKEK^184^, ^215^NLMAYVIMTFHNHVNDYK^232^, ^215^NLMAYVIMTFHNHVNDYKWIQR^236^ |
| 34 |  | 78 | MNTRYATCYVCDELVYLFKKTFSNMSPSAAAFYQQRMAIVKNGIVLCVRCSSKLKITNGISIPIYPNRRAQQHARRSR | ^5^YATCYVCDELVYLFK^19^, ^5^YATCYVCDELVYLFKK^20^, ^21^TFSNMSPSAAAFYQQR^36^, ^54^LKITNGISIPIYPNR^68^, ^56^ITNGISIPIYPNR^68^, ^56^ITNGISIPIYPNRR^69^ |
| 61 |  | 133 | MSNLMKNFFTELVKSTTFTTKVSVVKTTLSDWLCEQVYPDKDFSLKLKRVINMFLNDEIENDKIYKLVETVDSSNKLSRRQVDFLIHALLNNVSVTFTLHRFVDDNVLTQDELSFLANFLVTKLDEAYQLPAY | ^27^TTLSDWLCEQVYPDK^41^, ^27^TTLSDWLCEQVYPDKDFSLK^46^, ^50^VINMFLNDEIENDK^63^, ^50^VINMFLNDEIENDKIYK^66^, ^124^LDEAYQLPAY^133^ |
| 100 | PK2 | 225 | MKPEQLVYLNPRHHRIYFASPLNEYMLSDYLKQRNLQIFAKTNIKVPADFGFYISKFVDLVSAVEAIHSVNIVHHNINPEDIFMTGPDFDLYVGGMFGSLYKTFIKNNPQNITLYAAPEQIKKVYTPENDMYSLGIVLFELIMPFKTALERETTLTNFRNNVQQMPASLSQSHPKLTEIVCKLIQHDYSQRPNAKWLLKEMEQLLLEYTTGSKKTIKEGFGDKAI | ^16^IYFASPLNEYMLSDYLK^32^, ^42^TNIKVPADFGFYISK^56^, ^46^VPADFGFYISK^56^, ^107^NNPQNITLYAAPEQIK^122^, ^107^NNPQNITLYAAPEQIKK^123^, ^160^NNVQQMPASLSQSHPK^175^ |
| 15 | PKIP | 169 | MSCILTAFCKKNQANLNSLITLQNKKVKNYYVKNNETAIDKMLCIAADIKGQVEQLELVNQYLDAPKSEKLEFVYNCSDLDINEEDLKSLCLTKNIAYFTERYNAPTVLKAQPAVYDAFIKHSELFINAICQMDEKQQVNNFCLDELVKLKLIAIKHLCALEYLIENSI | ^12^NQANLNSLITLQNK^25^, ^12^NQANLNSLITLQNKK^26^, ^51^GQVEQLELVNQYLDAPK^67^, ^111^AQPAVYDAFIK^121^, ^122^HSELFINAICQMDEK^136^, ^137^QQVNNFCLDELVK^149^, ^157^HLCALEYLIENSI^169^ |
| 119 | ODV-E18 | 101 | MIYTDPTTGATTSTDVQSANYLNRLTPNMFLTILAVVVIIALIIMFVQSNSNNGNSSGAGSGGNGGGGGAATPPSATGFMNPLNATMRANPFMNTPQRQML | ^1^MIYTDPTTGATTSTDVQSANYLNR^24^ |
| 23 | SOD | 151 | MKAICIISGDVHGKIYFQQESANRPLKISGYLLNLPRGLHGFHVHEYGDTSNGCTSAGEHFNPTNEDHGAPDAEIRHVGDLGNIKSVGYNSLTEINMMDNVMSLYGPHNIIGRSLVVHTDKDDLGLTEHPLSKTTGNSDGRLGCGIIAICK | ^15^IYFQQESANRPLK^27^, ^28^ISGYLLNLPR^37^, ^86^SVGYNSLTEINMMDNVMSLYGPHNIIGR^113^, ^114^SLVVHTDKDDLGLTEHPLSK^133^, ^142^LGCGIIAICK^151^ |
| 48 |  | 83 | MLPKYYQNLPYNGKRIFEKFYDRSLQKYKSTHIATKLACCAVRKKYILIDCKWQPRPDANNSDTTTTNTEDSTTDTETEDEIA | ^5^YYQNLPYNGK^14^, ^5^YYQNLPYNGKR^15^ |
| 26 | Ubiquitin | 77 | MQIFIKTLTGKTITAETEPAETVADLKQKIADKEGVPVDQQRLIFAGKQLEDSKTMADYNIQKESTLHMVLRLRGGY | ^12^TITAETEPAETVADLK^27^, ^55^TMADYNIQKESTLHMVLR^72^ |
| 76 |  | 161 | MATSKTIVLYLCQAPATASLYVSADTDADEPIIYFENITECLTDDQCDKFTYFAELKQEQALFMKKVYKHLVLKNEGVFNKHHVLFDAMIMYKTYVNLVDESAFGSNVINYCEQFITAIFEIFTLSSKIVVAVPVNWENDNLSVLLKHLHNLNLIGIEIVN | ^50^FTYFAELK^57^, ^82^HHVLFDAMIMYK^93^, ^129^IVVAVPVNWENDNLSVLLK^147^, ^148^HLHNLNLIGIEIVN^161^ |
| 94 |  | 424 | MRKYALLQKMIINELLFLNDNVNYATNKLFSKDQANGELQKLSAMLLNYKKSNKNVPNIKFDLKNLSFMLENTDKIDIIQFDDVKNYVQPAIVNLFESHNRSLNNYSTELNTLLENGNENLVPNITDIDNIKLSHMQLARLLCYTAVVESRNSKPWKAIFNNDTCVLTDSFFNYIMNILNMIKTNQGSLAHNLSVVYHIENIQMNLQNKLKPRSITIEIVDKDKFENKHSDIEVCYVMNNRLHPQDVNSQQTLMCSSFVELNALPFCLYNETLPDDQSMSVFNLYKFEQTKNNSVSKPSRLGNVMFVNSLIDKPITRETIVDIINSYHNACQNLKRSGHRVVGDYRAYERDYKLAALDFIILMLVTSITHRTLKYNMLNIHEKMFQELKTIVCKHSAAKLYDILINYDINKEPLNNFRYNYEVL | ^301^LGNVMFVNSLIDKPITR^317^, ^318^ETIVDIINSYHNACQNLK^335^, ^400^LYDILINYDINKEPLNNFR^418^ |
| 37 | ODV-E66 | 702 | MSTVLIIVVVVIFLICFWCLLNSSNNSNNKNDANRNNVFVDFDPLPFDAFDINTFDITNDDSIVAFQQNNIQELENFEQWFKNNLSYSFSQKAEKVVNPNRNWNDNTVFDNLSPWTSVPDFGTVCHTLIGYCVRYNNTSDALYQNPELAYNLINGLRIICSKLPDPPPHQQAPWGPVADWYHFTITMPEVFMNITIVLNETQHYDEAAALTRYWLGLYLPTAVNSMGWHRTAGNSMRMGVPYTYSQMLRGYLLAEIRQEQGIQEILNTIAFPYVTRGNGLHVDSIYIDHIDVRAYGYLINSYFTFAYYTYYFGDEVINTVGLTRAIENVGSPEGVVVPGVMSRNGTLYSNVIGNFITYPLAVHSADYSKVLTKLSKTYYGSVVGVTDRLAYYESDPTNNVQAPLWTMARRIWNRRGRIINYNANTVPFESGVILQSLNGIMRIPSGTTSTQSFRPAIGQTAIAKTDTAGAILVYAKFAEMNNLQFKSCTLFYDHGMFQLYYNIGVEPNSLNNTNGRVIVLSRDTSVNTNDLSFEAQRINNNNSSEGTTFNGVVCHRVPITNINVPSLTVRSPNSSVELVEQIISFQSMYTATASACYKLNVEGHSDSLRAFRVDSDENIYVNVGNGVKALFNYPWVMVKENNMVSFMSANEDTTIQFSVIMNSFTSIGEPALKYSPSNCFAYGNGFKLNDSTFDLQFIFEIV | ^135^YNNTSDALYQNPELAYNLINGLR^157^, ^258^QEQGIQEILNTIAFPYVTR^276^, ^344^NGTLYSNVIGNFITYPLAVHSADYSK^369^, ^557^VPITNINVPSLTVR^570^,  ^629^ALFNYPWVMVK^639^ |
| 59 |  | 99 | MNTSVDAVTKLIRLQNDVLDMMREVDQYLNSDTPDYTIESLNAPGKQFDFLDEMLTKKLIESNAMVFDETNKNLKFIHNSISICLNRCINLITIKHYVQ | ^14^LQNDVLDMMR^23^, ^24^EVDQYLNSDTPDYTIESLNAPGK^46^, ^47^QFDFLDEMLTK^57^, ^59^LIESNAMVFDETNK^72^ |
| 47 |  | 171 | MYQIPDMLYNEKMPPRAKKLFVEAFTKYHKMNGGDEDIAMHKARKALEEKYVKINTLKNSWIPRKAAYEIVRDDIDESDDNADNSTKTKSNDRLNNENTSTDYDTEDEERVNTFNSRKRRVVAKRAFNSKSKKIPIGKVVSTPRKRLKQHTNTPPHYDTSEDEDEDNYYNY | ^20^LFVEAFTK^27^, ^66^AAYEIVRDDIDESDDNADNSTK^87^, ^94^LNNENTSTDYDTEDEERVNTFNSR^117^, ^147^LKQHTNTPPHYDTSEDEDEDNYYNY^171^ |
| 122 |  | 201 | MNVNLYCPNGEHDNNIFFIMPSKTNAVIIYLFKLDTEHPPTYNVNAKTRLVSGYENSRPININLRSITPSLNGTRGAYVISCIRAPRLYRDLFTYNKYTAPLGFVVTRTQAELQVWHILSVRKTFEAKSTRSVTGMLAHTDNGPDKFYAKDLMIMSGNVSVHFINNLQKCRAHHKDIDIFKHLCPELQIDNSVVQLESHSS | ^24^TNAVIIYLFK^33^  ^50^LVSGYENSRPININLR^65^  ^98^YTAPLGFVVTR^108^, ^182^HLCPELQIDNSVVQLESHSS^201^ |
| 118 |  | 476 | MSGGGNLLTLERDHFKYLFLTSYFDLKDNEHVPSEPMAFIRNYLNCTFDLLDDAVLMNYFNYLQSMQLKHLVGSTSTNIFKFVKPQFRFVCNRTTVDILEFDTRMYIKPGTHVYATNLFTSNPRKMMAFLYAEFGKVFKNKIFVNINNYGCVLAGSAGFLFDDAYVDWNGVRMCAAPRLDNNMHPFRLYLLGEDMAKHFVDNNILPPHPSNAKTRKINNSMFMLKNFYKGLPLFKLKYTVVNSTKIVTRKPNDIFNEIDKELNGNCPFIKFIQRDYIFDAQFPPDLLDLLNEYMTKSSIMKIITKFVIEENPAMNGEMSREIILDRYSVDNYRKLYIKMEITNQFPVMYDHESSYIFVSKDILQLKGTMNAFYAPKQRILSILAINRLFGATETIDFHPNLLVYRQSSPPVRLTGDVYVVDKNEKVFLVKHVFSNTVPAYLLIRGDYESSSELKSLRDLNPWVQNTLLKLLIPDSV | ^94^TTVDILEFDTR^104^, ^379^ILSILAINR^387^ |
| 92 |  | 391 | MECPFQIQVCISDRFFAFPHNLVEPQSDVGNKLIENLIVYVPTDDDRLYIDKKQFPKFNSVLVYRHEHDVNIDSRSPKKTASATIVYWNPLMPITEIGAGETRVFSVLLTNNLFYCNTMIIQHENPKCPIEFTYPETDMQSACSALLKNRNGQSVPPPIKSNLRPIACEIPLSHFKELVESNDFLLCFNLETSTMVKILSLKRIFCIFQYRKQPARYVINLPHEEIDNLYNKLNWERTRRLMKGDVPSNCATVNRSSLKYIKQAQSLLGIPDYSQTVVDFVKMFQKIIFPYQLVPNVIIKLNNFDQIMSVAPNKAESYKKIRLFCKNDSIAISSNGIVPINMPDFSPSNNAFDYSDYVNRTNINFVTQRVLIDGGFSSGVTVTPIKYNYYL | ^58^FNSVLVYR^65^, ^204^IFCIFQYR^211^, ^263^QAQSLLGIPDYSQTVVDFVK^282^, ^287^IIFPYQLVPNVIIK^300^ |
| 124 | ODV-E56 | 375 | MSFFTNLRRVNKLYPNQASFLADNTRLLTSTPAGFTNVLNAPSVRNLGNNRYQPGYQLSNNRFVSTSDINRITRNNDVPNIRNVFQGISDPQINSLRQLRRMDNVPDFHYHTKQTRSNAVRQNFPETNVRTPEGVQNALQQNPRLHNHMRTLKVAGVGILLAGGGYLLFTASTLVQDIINAINRTGGSYYVQGRNAGENVESCLLLQRTCRQDRNLAQSDVNICSRDPLLANDSPLLTNMCQGFNYETEKTVCRGSNPAANPNSPQYVDISDLPAGQTIMCIEPYSFSDLVGDLGLDWLLGREGLVGKSSNSSDGIRNKIMPIIMMIGAVLFLGLILYFIYRYMTKGGGGGGGSGGAPTPIVIMQHPASTTAPRR | ^10^VNKLYPNQASFLADNTR^26^, ^13^LYPNQASFLADNTR^26^, ^27^LLTSTPAGFTNVLNAPSVR^45^ |
| 112 | P35 | 299 | MCVIFPVEIDVSQTVIRDCHVDEQTRELVYINKIMNTQLTKPVLMMFNISGPIRSVTRKNNDLRDRIKSKVDEQFDQLEREYSDKIDGFHDNIQYFKDEHYSVSCQNGSVLKSKFAKILKSHDYTDKKSIETYEKYCLPQLVDKHNDCYVAVCVLKPGFENGSNQVLSFEYNPIGNKVIVPFAHEINDTGLYEYDVLAYVDSVEFDGKQFEEFVQKLILPSSFNDSEKVLYYNEASKNKNMIYKALEFTTESSWVKSNKFNWKIFCNGFIYDKKSKALYVKLHNVTSTLNKNVILDMIK | ^34^IMNTQLTKPVLMMFNISGPIR^54^, ^217^LILPSSFNDSEK^228^ |
| 66 | GP41 | 403 | MTDERGNFYYNTPPPPLRYPSNPATAIFTNAQTYNNAPGYVPPTTRDNKMDTSRSNSTNSVAIAPYNKSKEPTLDAGESIWYNKCVDFVQKIIRYYRCNDMSELSPLMIHFINTIRDMCIDTNPINVNVVKRFESEETMIRHLIRLQKELGQGNAAESLPSDSNIFQASFVLNSLPAYAQKFYNGGADMLGKDALAEAAKQLSLAVQYMVAESVTCNIPIPLPFNQQLANNYMTLLLKHATLPPNIQSAVESRRFPHINMINDLINAVIDDLFAGGGDYYHYVLNEKNRARIMSLKENVAFLAPLSASANIFNYMAELATRAGKQPSMFQNATFLTSAANAVNSPAAHLTKNACQDSLTELAFQNETLRRFIFQQINYNKDANAIIAAAAPNVTRPNTKGRTV | ^325^QPSMFQNATFLTSAANAVNSPAAHLTK^351^, ^371^FIFQQINYNK^380^ |
| 63 | VLF-1 | 379 | MNGFNVRNENNFNSWKIKIQSTPRFESVFDLATDRQRCTPDEVKNNSLWSKYMFPKPFAPTTLKSYKSRFIKIVYCSVDDVHLEDMSYSLDMEFDSIENQTLLIDPQELCRRMLELRSVTKETLQLTINFYTNMMNLPEYKIPRMVMLPRDKELKNIRKKEKNLMLKNVIDTILNFINDKIKMLNGDYVHDRGLIRGAIVFCIMLGTGMRINEARQLSVNDLNVLIKKGKLHSDTINLKRKRSRNNTLNNIKMKPLELAREIYSRNPTILQISKNTSTPFKDFRRLLEESGVEMERPRSNMIRHYLSSNLYNSGVPLQKVAKLMNHESSASTKHYLNKYNIGLDETSSEEENNNNDDDAQHNRNSSGSSGESLLYYRNE | ^25^FESVFDLATDR^35^, ^52^YMFPKPFAPTTLK^64^, ^216^QLSVNDLNVLIK^227^, ^286^LLEESGVEMERPR^298^ |
| 62 |  | 85 | MNLYLLLGALTIFSLVYDKKENSIFLYLLILFLVFIIVVSPAIISKNTESNVEDIPSHKAKSVRKKLEIEQALDAILNKNTSSID | ^66^KLEIEQALDAILNK^79^, ^67^LEIEQALDAILNK^79^ |
| 9 |  | 210 | MNLKVILTPINLKGEEEPNCVERITIMPCSLMDTEICLNVRCRSPFAKFKVLIIVDGFDSAYIQATFCSISDSVTIVNKFNEKHVMFDGFVRPDDEGTTMPYVIGPLYSVDAAVADRKVKDMVNSIENQQTMLKVFINEANVYNKWNMLKGLIYNNNNNESVLVNNVVKFIKVDKNYNVTKKNNVTKWVPAVNYFTGRQLLTILFIFKFK | ^5^VILTPINLKGEEEPNCVER^23^, ^188^WVPAVNYFTGR^198^ |
| 8 | BV/ODV-  E26 | 229 | MNSVHTRLCASSNQFAPFKKRQLAVPVGSVNSLTHTITSTTVASVIPKNYQEKRQKICHIISSLRNTHLNFNKIQSVHKKKLLHLQNLLRKKNEIIAELVRKLESAQKKTTTTHRNISSKPAAHWKYFGVVRCDNTIRTIIGNEKFVRRRLAELCTLYNAEYVFCQARADGDKDRQALASLLTAAFGPRVIVYENSRRFEFINPDEIASGKRLIIKHLQDESQSDINAY | ^176^QALASLLTAAFGPR^189^ |
| 129 |  | 77 | MDSSNCIKIDVKYHMPLHYQCDINADKNVVNAYDAIDVDPNKKFIINHNHEQVDETNKQEVVDKTDATTYNSCIIKI | ^13^YHMPLHYQCDINADK^27^, ^28^NVVNAYDAIDVDPNKK^43^, ^44^FIINHNHEQVDETNKQEVVDK^64^ |
| 49 | FP25K | 214 | MDQFEQLINVSLLKSLIKTQIDENVSDNIKSMSEKLKRLECDNLTDSVEIYGIHDNRLNNKKIRNYYLKKICILLNLNFKHVIESSFDKNHIVAKLCDATRAKEWQTMSRERRLKNFNLNINYDGPVKIFVAATAEQKLLLKKTRDALLPFYKYISICKNGVMVRRDEKSRVFIVKNEQNIEYLKANKYYAFHSDSVDNFEFENDSKKMLQNLI | ^39^LECDNLTDSVEIYGIHDNR^57^ |
| 78 | Helicase | 1222 | MIDNILQFFLKDVPEDKTYEINNLQDANHLIIRNTRTGTRRLFEYVNNFQQFLNTIRNNFNGPCAKHDMGSSCEDTEEATEKQAVQQTLDGHDWVLESNDFCIFVKPFILKKHYEVIQKYINFENFFKSTDPGYINKCVQAGDYYYWPNWPKGQAFSFNGWQLFLNIKFGIVIEPTIPIIHNKKLGPVDLFVFDPKYFLNVELSLRTNHDPPQTLFVNGKTKFDESHEDLFILKMADGTVVTCKVNGELVNSDKNFFNYIRDDINLEECITVPKYKHIVNVNLKSLRVFEDNNFDKNDVDLSDTRSRKPRIVPIISASSENADYIQTQINLGLIAIYENMVKVLATHEQANDPNLLQQYFEKSKFKNFDFLIYVLWKILTKNENFSYRETDIKLFLELLCESLFACDKEILNEALKRCEPYKKQEKVVFNRTCNHWFDFDDTKLCVSLGYYYGIHYMIYLTLSAKNETLDDDELWAYTYENVMALNLPPDIVCKGFFRKLENVVTGVNLVFNGKNYQIVKKEDDLFKLVKSNCYKLSNIKFNNWKYLYLTTYGVYNLFTNSFHSNCPFLLGTTLPQTLKKPTDEEYLPEDAFNYMLSTSTDELSIYRIYHIAKMCRDVKMLKTNMAIVNYMGNCNTCQADMRVALNNLFRDLWNLDDENLITLALYVNKNKVSDMLHNLKCKPCRSTVSGSRPKCKCYKKIKINRKALKVCLIADMFGNDAELSKLIWMLIFTNKTYVSTTLIRTNSEFVNQHGEFFFKEHNKIIQYLYQTIHKIEYVDMLMDKFNDKRLFLTELRDDVAREPDVQFEESDNICKFYTHHTDALIILKKYNVWWDKIILARSTDDLPTWLTRFYMRIIMSKMDLKEYSYNYLKKIVEGYLYFKRFTNFNHANAIMLMHFAASLAIPVDYGKKAIYMPGEPGSGKSSFFELLDYLVLMHKFDDDNHSGESNKETSDKEVSKLNSQLYVINELKQCSESYFKKHADSSKSDSKSRKYQGLLKYEANYKMLIVNNKPLYVDDYDDGVQDRFLIVYTNHKFVDSVKFAGSVYEHIKSKQFPIESMYYESLVTPVRLFLSHVLMYRRDPKTGFVVYKTLLNNDPMHKHNLMCMSTNNSPLYALIYILNIKTVRNATITIGEDKMEEMISIAVQHLKNFLHPSFVQYNYKKNINASSSKSFVFNEQVLLQQIKNKFKNNYNKTTNVFYNMTMALNRNDLNTSVPNFVC | ^1174^SFVFNEQVLLQQIK^1187^ |
| 2 | ORF1629 | 542 | MTNRKYESVQSYLFNNRNNKIDAHQFFERVDTAEAQIIKDSIYDNTVLLNRDVFLNILKFANDVFDNKAYMYVDDSEVSRYYNAVVKMKRLVINVRDPSLRQSLYNTIAYIERLLNIGTVNDSEITMLIADFYDLYSNYNIELPPPPPQALPRSRRPSVVQPAAPAPVPTIVHEQTKPEQIIIPAAPPPPSSVPNIPAPPPPPPPPPSSMSELPPAPPMPTKPQPAAPLDDRQQLLEAIRNEKNRTRLRPVKPKTAPETNTIIEVPTTVLPKEPKPPSASPPPPPPPPAPPAPPPMIDLSSAPLQPPLVDLPAEMLPPPAPSLSNVLSELKSGTVRLKPAQKRPQSEIIPKNSTTKNLIADVLVDTINRRRVAMAKSSSEATSNDEGWDDGGNNRPNAPDVKYVQALFNVFTSSQLYTNDSDEKNTKAHNILNDVESLLQNKTQTNIDKARLLLQDLASRVVLSENPLDSPAIGLQKQPLFETNRNLFYKSIEDLIFKFRYKDAENHLIFALTYHPKDYKFNELLKYVQQLSVNQQRTESNA | ^357^NLIADVLVDTINR^369^ |
| 121 |  | 95 | MLLLIIFLILIKVLIFKKMNQTHLKCHSDKICPKGYFGLNADPYDCTAYYICPHKVQMFCESNHEFDLDSASCKPIVYDRTGSGCAARMYRNLLL | ^35^GYFGLNADPYDCTAYYICPHK^55^ |
| 79 |  | 182 | MLSIILAIVFIFFVLIYLIISIKNHHPFLHRIKTLIKDFNNTLLFGAYIQIYDLSTPARTERLFIIAPENVVLYNFNKTLYYYLDSANVFCPNEFSVTTFTQSTIKTINETGIYATACTPVSSLTLIEHFVTLKNNVPDHTLVLDVVEKQIQFSILDIINYLIYNGYVDLDNQNVSSTDQVY | ^63^LFIIAPENVVLYNFNK^78^ |
| 72 | VP39 | 350 | MALMPVGMAPRQMRVNRCIFASIVSFDACITYKSPCSPDAYHDDGWFICNSHLIKRFKMSKMVLPIFDEDDNQFKMTIARHLVGNKERGIKRILIPSATNYQEVFNLNSMMQAEQLIFHLIYNNEEAVNVICDNLKYTEGFTSGTQRVIHSVYATTRSILDTTNPNTFCSRVSRDELRFFDVTNARTHRGGVGDQLFNNYSGFLQNLIRRAVAPEYLQIDTEELRFRNSATCIIDETGLVASVPDGPELYNPIRSSDIMKSQPNRLQIRNVLKFEGDTRELDRTLSGYEEYPTYVPLFLGYQIINSENNFLRNDFISRANPNATLGGGVGALAGPAPGVVLGEAGGGVAA | ^62^MVLPIFDEDDNQFK^75^ |
| 38 | ETS | 89 | MIERTVTRWHLLSDNVLLKIGEVAQRLNYYLQEYANLEMQIEEEIKYMEIDDGEEIDTVKTFLRNSMSTAEQRDLYALALKLNSLINNI | ^9^WHLLSDNVLLK^19^ |
| 87 | P45 | 387 | MCAYRLQYSLRFNTYDRFENVCFEAQLLRDEIDSLCFLFSKYFNQSLIVDGKGLTFFTEFNKCIVSIKSSFENQANNTDNIHNVKNIFSIFLRDEFIKQVPQFRTIMQYLQTYYNPTPAPDVDEIMCQSCKPANKIQCFECKCRYLASSLSTLDEGLQNGWDIFLRPMFGMPLMLYVLLRTDYKNESDVINENNLITQIFVQFFYNLICDKAYSLYTKRDMCVPFVKECKKATIGLRQEDHERVLSILNAQCNGFSTAANGNRLLLPFKNFMIKMGRNTNMKKVNKIASTVLIGFYLRHYLESLPNKAYPVAELELRNVCRFIMSKYSDENINLLIHKLKLIKIDICNVLMTEMIVPESFIRHIITKYQLDNEISLLIELNHDCFNK | ^53^GLTFFTEFNK^62^, ^308^AYPVAELELR^317^ |
| 98 |  | 82 | MSILNVVEACDLAHTFLKLGYLFRAKTCLDIALDNLKLLRRKTNIKEVAVMLNKKTTECLQLKQKIDKKIAQRILIKIYTIK | ^27^TCLDIALDNLK^37^ |
| 57 |  | 262 | MLQQKLNKLKDNLNTFSSKSVVCARSKLFDKRPTRRPRCWRKLLEIDKKFHVCRHVDTFLDLCGGPGEFANYTMSLNPLCKAYGVTLTNNSVCVYKPTVCKRKNFTTITGPDKSGDVFDKNVVFEISIKCGNACDLVLADGSVDVNGRENEQERLNFDLIMCETQLILICLRPGGNCVLKVFDAFEHKTIQMLNKFVNHFEKWVLYKPPSSRPANSERYLICFNKLVRPYCNDYVNELEKQFKKYYRIQLKNLNKLINLLKI | ^82^AYGVTLTNNSVCVYKPTVCK^101^ |
| 19 | LEF-6 | 173 | MVFDVYYNGYYVEKKFSKEFLIHIAPDLKNSVDWNGSTRKLLRVLNKRAYRQVLQCNGRYYWPDGTKFVSHVYNRSIRTHSTTVKRTDSSHRLKSQVVDKRPRRSLDSPRLDGYVLASSPIPHTDWNEELKLYAQSHGYDDDDDDLEDGEIDERDSLKSLNNHLNDLNVLEKQ | ^19^EFLIHIAPDLK^29^ |
| 93 |  | 67 | MDNYSLHNFYNNNRKPLKPTTLHDGNIKKSVYENITYVRKLMCKENMPGEHDHKFYNRGYNKENKYK | ^30^SVYENITYVR^39^ |
| 29 |  | 217 | MRNAAGLFMIIEPDKAVLLCARRAYRSANAPAADINDTFLEKISIPRGHRDCCDAKVYETAVREFVEETGRFFDSAFIYKFPFTLQWKDDGVTYKYLIYVGVVRGNLIDVNAKPNTYTVKLLPGTFGNDYRIMLKPRRFNCEITRSLAIVPLNKYFNYMNDKQLITYDYSNYIEFFSFVRSIKKRFDNRQLQDFFYATLKKIDNNDAPQKLHALRRV | ^190^QLQDFFYATLK^200^ |
| 65 |  | 104 | MATTLYTNKVWCVYILRQDNGKLYTGITSNLNRRIKQHSNKQGAKCLRNATNLRLVYHSASAYDYKTAARMEYNLKRKCSKYFKLRLIKAKPLLLHKFLLANKL | ^23^LYTGITSNLNR^33^, ^98^FLLANKL^104^ |
| 104 | Cysteine proteinase | 323 | MNKILFYLFVYAVVKSAAYDPLKAPNYFEEFVHRFNKNYSSEVEKLRRFKIFQHNLNEIINKNQNDSAKYEINKFSDLSKDETIAKYTGLSLPTQTQNFCKVILLDQPPGKGPLEFDWRRLNKVTSVKNQGMCGACWAFATLGSLESQFAIKHNELINLSEQQMIDCDFVDAGCNGGLLHTAFEAIIKMGGVQLESDYPYEADNNNCRMNSNKFLVQVKDCYRYIIVYEEKLKDLLPLVGPIPMAIDAADIVNYKQGIIKYCFDSGLNHAVLLVGYGVENNIPYWTFKNTWGTDWGEDGFFRVQQNINACGMRNELASTAVIY | ^102^VILLDQPPGKGPLEFDWR^119^, ^209^MNSNKFLVQVK^219^, ^224^YIIVYEEK^231^, ^303^VQQNINACGMRNELASTAVIY^323^ |
| 7 | EGT | 506 | MTILCWLALLSTLTAVNAVNILAVFPTPAYSHHIVYKVYIEALAEKCHNVTVVKPKLFAYSTKTYCGNITEVNSDMSVKQYKKLVTNSAMFRKRGVVSDTDTVTAANYLGLIEMFKDQFDNINVRNLIANNQTFDLVVVEAFADYALVFGHLYDPAPVIQIAPGYGLAENFDTVGAVARHPVHHPNIWRNNFDDTKANLMTEMRLYKEFKILANMSNALLKQQFGPDTPTIEELRNKVQLLLLNLHPIFDNNRPVSPSVQYLGGGIHLVKSAPLTKLSPVIDAKMNKSKSGAIYVSFGSSIDTKSFANEFFYMLINTFKALDNYTILWKIDDEVVKNITLPANVITQNWFNQRAVLRHKKMAAFITQGGLQSSDEALEAGIPMVCLPMMGDQFYHAHKLQQLGVARALDTVTVSSDQLLLAINDVLFNASTYKKHMAELYALINNDKATFPPLDKAIKFTERVIRYRHDISRRLYSLKTTAANVPYSNYYMYKSVLSIVMNHIAHF | ^222^QQFGPDTPTIEELR^235^ |
| 130 | PTP | 168 | MFPARWHNYLQCGQVIKDSNLICFKTPLQPELFAYVTSEEDVWTTEQIVKQNPSIGAIIDLTNTSKYYDGVHFLRAGLLYKKIQVPGQTLPSESIVQEFIDTVEEFTEKCPGMLVGVHCTHGINRTGYMVCRYLMHTLGIAPQEAINRFEKARGHKIERQNYVQDLLI | ^133^YLMHTLGIAPQEAINR^148^ |
| 69 | P95 | 839 | MMSGVMLLVFAIFLIIAFTLIYLAIYFKFDETTYTKRLQVMIEYIKRTNADEPTPNVIGYVSDITQNTYTVTWFNTVDLSTYQESVHDDRNEIFDFLNQKLQPVDRIVHDRVRANDENPNEFILSGDKDDVTMKCPAYFNFDYAQLKCVPVPPCDNKPAGRYPMDERLLDTLVLNQHLDKDYSSNEHLYHPTFYLRCFANGAHAVEECPDNYTFDAKTRQCKVNELCENRPDGYILSYFPSNLLVNQFMQCVSGRHVVRECPANKIFDRNLMSCVEAHPCTFNGAGHTYITADISDAQYFKCLNNNESQLMTCINRIRNSDNQYECSGDSRCIDLPNGTGQQVFKHADDDISYNSGQLVCDNFEIISNIECDQSNVFENKLFMDKFRLNMQFPTEVFDGTACVPATADNVNFLRSTFAIENIPNHYDIDMQTSMLGKIEMIKQLVSKDLSLNNDAIFAQWLLYARDKNAIGLNPLTGEPIDCFGNNLYDVFDARRANVCKDLGKSVLKTLNFGDGEFLNVLSDTLTGKDEDYRQFCAISYENGQKIVENEHFQRRILTNILQSDVCANIYTTLYQKYTTLNPKYTTTPLQYNRILVKRPKNIEIYGANTRLKNATIPKNATTISPVFNPFENQPNNRQNDSISPLFNPFQTTDAVWYSEPDGDEAVAPPPTAPPPPSEPEPEPEPELPSPLILDNKDLFYSCHYSVPFFKLTSCHAENDVIINALNELRNNVKVDADCESAKDLSHVLNAYAYVGNGIGCRSAYDGDAIVVKKEAVPSHVYANLNTQSNDGVKYNRWLHVKNDQYMACPEELYDNDEFKCNVESDKLYYLDNLQEDSIV | ^619^NATTISPVFNPFENQPNNR^637^ |
| 117 | EXON0 | 261 | MIRTSSHVLNVQENIMTSNCASSPYSCEATSACAEAQQVMIDNFVFFHMYTADIQIDAKVQCGVRSAAFAIIDDKHLEMYKYRIENKFFYYYDQCADIAKPDRLPDDDGACCHHFIFDAQRIIQCIKEIEGAYGVRDRGNVIVFYPYLKQLRDALKLIKNSFACCFKNINSMQMYVNELISNCLLFIEKLETINKTVKVMNLFVDNSVLYECNVCKEISTDERFLKPKECCEYAICNACCVTMWKTATTHAKCPACRTSYK | ^139^GNVIVFYPYLKQLR^152^ |
| 116 | ME53 | 451 | MNRFFRENNIFDAPKTGGKGRVKSLPTPVANSPLSPVRQPPKSNIKPPTRISLPTRTFSANPLERSISSSIVSKKPVVNRKDGYFVPPEFGNKLESLPAYSDKLDFKQERDLRMHFMSDLERNIMKATLKFSTNYIMGYINSKDMRMTGKFASSSVKYKKTTEQHMSDSRCTTCNYRFKDNTRAWFLYVVVHIEKPLDDSDRIDICCQKCYLYHNVPKTSYEIYPSINLVDLSYLARERFFYQYIFPVSLEHTTEVKELKIDDHNCKVFEIIRRIIRNHKEPNERIQTIDLSTTGGLVLRETYTNIVLQRYRSMCTRPDAADDVNCFILQEPSEMMAALQDNRFSGIKGTVFATVKVKKFTEVLDGGAITFPSRPTINNYCKLCKKTKLYYKNPVLYCTKCGFTNVYHFPEYSKLVYYSKAIKNFEMHNEMIIYYDLKMYKKLINIVNNNV | ^443^LINIVNNNV^451^ |
| 85 |  | 362 | MSAIALYLEINKLRLKIDEPMQLAIWPQIFPLLCDEHQNVQLNTDVLINFIMHVARKSQNTILNNNAAIASQYAAGNAGVVVAAPASAHPTPRPVINLFARANAVAPAQPSEELINMRRYRNAARKLIHHYSLNSTSSTEYKISDVVMTMIFLLRSEKYHSLFKLLETTFDDYTCRPQMTQVQTDTLLDAVRSLLEMPSTTVDLTTVDIMRSSFARCFNSPIMKYAKIVLLQNVASQRDKRTTLEELLIERGEKIQMLQPQQYINSGTEIPFCDDSEFLNRLLKHIDPYPLSRMYYNAANTMFYTTMENYAVSNCKFNIEDYNNIFKVMENIRKHSNKNLNDQDELNIYLGVQSSNAKRKKY | ^328^VMENIRK^334^ |
| 125 |  | 299 | MYILIMLGLNIFIETVYNFSDHTMERYHPYQRAPWNASIFKKNYNNVKVASYIEVPYTTNNADVEMTDTTNAPSRPKSKARRRLDFTNMAPQPCCIDDGNFAKPLL | ^49^VASYIEVPYTTNNADVEMTDTTNAPSRPK^77^ |
| 67 |  | 234 | MTTTTTKTTQPPLSMSKKKTPTLLESISKKISTTETFQRLRNKNLTTLNKIKYDSELLLHYLYDDQQNKNSDYANNNINVIKISKVKVKKTGASILAHYFAQVHVSNGYSFEFHPGSQPRTFQTIHTDGLIIKVLILCDECCKKELRDYIKGENSFNVAFKNCESILCRRISFQTVLMTCAVLLLLFNVEKFSMINLLIILLILLSLFCHNNYIISNPCIEFCNHKSTNKKYDR | ^121^TFQTIHTDGLIIK^133^ |
| 114 | P10 | 70 | MSKPNVLTRILDAIAETNTKVDSVQTQLNGLEESFQPLDGLPAQLTDFNTKISEIQSILTGDTAPDPPDS | ^52^ISEIQSILTGDTAPDPPDS^70^ |
| 81 | BRO-c | 318 | MAQVKIGEFKFGEDTFTLRYVLGDEQPVRFVAKDIASSLKYVNCERAIRVHVDGKYKSTFEHADQIQHHAPDSVAKQGDPLYLHPHTVLITKSGVIQLIMKSKLPYAIELQEWLLEEVIPQVLCTGKYAPAVEMDTNDVIAKIDDLTQKLTVANADLAEANRSLILFANEMIVARRDAETARQDCENARRETAQLANRMADIAQDVIAKPSNPQLCHSLAVCDVGNNEFAFLRPQKRSLGRSLKRLGSNDVIFSSDYVPNSMNVLNKVKEAIPRNKFKAKHNRITLLEDYTREELMNVIGSTMTDRQIARMNSLRNAQ | ^30^FVAKDIASSLK^40^, ^177^DAETARQDCENAR^189^ |
| 107 | GP16 | 106 | MNFWATFSICLVGYLVYAGHLNNELQEIKSILVVMYESMEKHFSNVVDEIDSLKTDTFMMLSNLQNNTIRTWDAVVKNGKKISNLDEKINVLLTKNGVVNNVLNVQ | ^96^NGVVNNVLNVQ^106^ |
| 115 | P74 | 645 | MAVLTAVDLTNASRYAIHMHRLEFISRWRTRFPHILIDYTLRPASSDDDYYVPPKLADRALAVKLTFSKRGCESMSCYPFHETGVVSNTTPFMYTQTSETRVGYAQPACYHLDRAAAMREGAENKVQSAEFRYTPDNRCILVDSLSKMYFNSPYLRTEEHTIMGVDDVPAFNVRPDPDPLFPERFKGEFNEAYCRRFGRNLFNGGCSFRWWESLVGFVLGDTILVTFKMLANNVFSELRNFDYRAPSSILPPRPNVDSNAILAQWRSVRDTTTDLEFEKLFTKRPTLNDLGMIVDGSPVQITYTAETGFTKTPITYNYRGTERVHVEHFEALDRSISDQDLESIITKFLEDYAIIFGIATDAGFEMLMSGFKSMLKKINTTLIPALKRMLVSTTRRVTARILGETYKAAIVHSFNAIAIKTLTTTAKALTRIAIKASSVIGIVLILLTLADLVLALWDPFGYNNMFPREFPDDMSRTFLTAYFESLDNTTSREIIEFMPEFFSDMVETDDKATFESLFHLLNYVTSLEVNSDGQMLKLDEGDKIEDFDESTLVGKALATSSLYTRMEFMQYTFRQNTILSMNKKNNNFNQIIMGLFATNTIVAFTAFVIHKQLIFFIFFVIFLMITFYYIVKESYGYYKTIDLLF | ^229^MLANNVFSELR^239^ |
| 128 | PE38 | 309 | MPRDTNNRRRHRSTPYERPTLEDLHRQLEDALERRYQMRRRQRQNRLRTIQIKQQRMMAELKKEPVINFKFECSVCFETYSQQSNDTCPFLIPTTCDHGFCFKCVIDLQSNAMNIPHSIVCCPLCNTQVKMWRSLKPNAVVTCKFYKKTQERVPAVQQYKNIIKVLQERSVISVENSDNNCDINMENQAKIVALEAELKNEKNHSDQVTSENRQLIEENTRLNEQVQELQRQVRTLAPQRGITVNPQIGRDDRAPAELNERFRSLVYSTISELFIENRVHSIQNYVYAGTSGAASSCDVNVTVNFGFEN | ^222^LNEQVQELQR^231^ |
| 55 | LEF-3 | 385 | MATKRFFSGESSGEPLIKRMAMANSPKKIRENYKRINGKLMGKMTLSIDNEYYYTFRIMSDNKIQEYYGDSQSFKDMEEGKCYDISLNYVKTKFSQMIQINEYKECEMEIETAIPLSAYLTNKHFENEDSVNIIVKYKFIYKKINSNLYKIVFEVAYKNFNDDSDVVQVECFVNAKTLMNLFKNNIKGSDDINEVFKYLKDNENQIFTIYNIKCQQIFNGANVYMNWNVVNSTRIELCEAKENEAYSNLQNCTNARINISRSNKRVASYNVNMLKSELEENDMSDNKFIVQFKSDDLNVADSDDRSTSSDSSKWNKSVFYVNTNKKTEADSLQKLCADFNQISMLLEDNLIKVTIYVTVENGENGNMNVLGLLKYDEDENDYTFL | ^266^VASYNVNMLK^275^ |

**Supplementary Table S9. Host proteins identified in the M5 section of the gel.**

| Protein | Annotation | Size(amino acids) | Sequence | Sequence(s) of corresponding peptide(s) |
| --- | --- | --- | --- | --- |
| BGIBMGA002429-PA | Cyclophilin type peptidyl-prolyl cis-trans isomerase/cyclophilin-like domain (CLD) | 165 | MSLPRVFFDVTVDDAPLGKIVIELRSDVTPKTCENFRALCTGEKGFGYKGSIFHRVIPNFMLQGGDFTNHNGTGGKSIYGNKFEDENFTLKHTGPGILSMANAGADTNGSQFFITTVKTSWLDGRHVVFGNVVEGMEVVKQIETFGSQSGKTSKRIVIKDCGQIA | ^56^VIPNFMLQGGDFTNHNGTGGK^76^, ^77^SIYGNKFEDENFTLK^91^, ^92^HTGPGILSMANAGADTNGSQFFITTVK^118^, ^119^TSWLDGRHVVFGNVVEGMEVVK^140^, ^126^HVVFGNVVEGMEVVK^140^, ^126^HVVFGNVVEGMEVVKQIETFGSQSGK^151^ |
| BGIBMGA004881-PA | RNA recognition motif | 252 | MVPQTKVFVGSLPQGSKPEDLRKLFERFGVVTECDIMNRCGFVHMQTEEQAAAAIRALHNSTFNGGVISVERGRIKERGQRGGGRGGRGSMRGGMERRGGGPVRHAPPPRDAPYMRDRPGPVRGYERGPPAAPYDERYAYGEDRRGFALMDRGPRDAYAPPAPAYPDERYQNHISVVEGTRRYDEPPYDDRHYDERAQYERPRPAHHIPNGDMYSRRSPAPMYSGRGGPAPVAGGYERDHYVPQYMQPPLGR | ^7^VFVGSLPQGSKPEDLRK^23^, ^28^FGVVTECDIMNR^39^, ^57^ALHNSTFNGGVISVER^72^ |
| BGIBMGA002981-PA | Profilin | 126 | MSWQDYVDKQLMASRCVTKAAIAGHDGNVWAKSEGFEISKDEVAKIVAGFENESLLTSGGVTIAGTRYIYLSGTDHIIRAKLGKVGVHCMKTQQAVVISLYEEPIQPQQAASVVEKLGEYLITCGY | ^33^SEGFEISKDEVAK^45^, ^46^IVAGFENESLLTSGGVTIAGTR^67^, ^92^TQQAVVISLYEEPIQPQQAASVVEK^116^ |
| BGIBMGA007092-PA | Calponin homology domain(a family of [actin](http://en.wikipedia.org/wiki/Actin) binding domain) | 173 | MDVINSKYSEELAHESLEWIRMITGEPENTSGDMDNFYEVLKDGTLLCKLANNIHPNMIKKINTSSMAFKCMENINAFLEAARQLGVPAQETFQTVDLWERQNLNSVVICLQSLGRKAGTYGKPSIGPKEAEKNVRNFSEEQLRAGQGVISLQYGSNKGANQSGINFGNTRHM | ^8^YSEELAHESLEWIR^21^, ^22^MITGEPENTSGDMDNFYEVLK^42^, ^71^CMENINAFLEAAR^83^, ^84^QLGVPAQETFQTVDLWER^101^, ^102^QNLNSVVICLQSLGR^116^, ^145^AGQGVISLQYGSNK^158^ |
| BGIBMGA001106-PA | Ribosomal protein L21e | 159 | MTNSKGYRRGTRDLFARRFRTHGTIPLSTYMKVYKVGDIVDIRGNGAVQKGMPHKVYHGKTGRVYNVTAHALGVIVNKRVRGRIIPKRINIRVEHVKHSKCRQDFLKRVKENERLLKEAKAAGKTVNLKRQPAPPKAAHIVSGTEKPVLLAPIPYEFVA | ^64^VYNVTAHALGVIVNK^78^, ^64^VYNVTAHALGVIVNKR^79^, ^137^AAHIVSGTEKPVLLAPIPYEFVA^159^ |
| BGIBMGA006867-PA | Ribosomal protein S8 | 129 | MVRMNVLSDALKSIHNAEKRGKRQVLIRPCSKVIVKFLTVMMKHGYIGEFEIVDDHRAGKIVVNLTGRLNKCGVISPRFDVPINDIERWTNLLPSRQFGYLVLTTSGGIMDHEEARRKHLGGKILGFFF | ^79^FDVPINDIER^88^, ^97^QFGYLVLTTSGGIMDHEEAR^116^ |
| BGIBMGA013100-PA | LSm ([RNA](http://en.wikipedia.org/wiki/RNA)-binding [protein](http://en.wikipedia.org/wiki/Protein)) | 130 | MSIGVPIKVLHEAEGHVVTCETNTGEVYRGKLIEAEDNMNCQMTLVTVTYRDGRVAQLENVYIRGSKIRFLILPDMLKNAPMFKRQGNKPTAGRGKSAILRAQAAGRGRAGGRGGGHRGGWQGGSGPSRR | ^9^VLHEAEGHVVTCETNTGEVYR^29^, ^55^VAQLENVYIR^64^ |
| BGIBMGA001549-PA | Ubiquitin | 152 | MQIFVKTLTGKTITLEVEPSDTIENVKAKIQDKEGIPPDQQRLIFAGKQLEDGRTLSDYNIQKESTLHLVLRLRGGMQIFVKTLTGKTITLEVEASDTIENVKAKIQDKEGIPPDQQRLIFAGKQLEDGRTLSDYNIQKESTLHLVLRLRGGM | ^12^TITLEVEPSDTIENVK^27^, ^64^ESTLHLVLR^72^, ^131^TLSDYNIQKESTLHLVLR^148^ |
| BGIBMGA004331-PA | FK506 binding protein | 108 | MGVTVETISPGDESTYPKSGQTVVVHYTGTLTNGKKFDSSRDRGKPFKFRIGKSEVIRGWDEGVAKMSVGERAKLTCSPDYAYGQQGHPGVIPPNSTLIFDVELLRLE | ^2^GVTVETISPGDESTYPK^18^, ^19^SGQTVVVHYTGTLTNGKK^36^ |
| BGIBMGA011282-PA | Ribosomal S13/S15 N-terminal domain | 151 | MGRMHAPGKGISQSALPYRRSVPTWLKLTADDVKEQIYKLGKKGLTPSQIGVMLRDSHGVAQVRFVTGKKILRIMKAMGLAPDLPEDLYYLIKKAVAMRKHLERNRKDKDSKFRLILVESRIHRLARYYKTKSVLPPNWKYESSTASALVA | ^44^GLTPSQIGVMLR^55^, ^77^AMGLAPDLPEDLYYLIK^93^ |
| BGIBMGA009106-PA | glutathione S-transferase 2 | 416 | MVKTKAERLAQQRLCKRRKYAEIKNDPELWALEKEKQRAKYQKRKSEKKQTSITEKSPREKRQIRKNWKQNSKKYRQMPESFPEETNNHVVDPIGLFTKEDLQKAQRKIRITWNYTEAGHGKGGPDGVGAVLKRTADRLVNFGRDVGDFESFCEIIKDNVESVVIKIVNEETIKQKENLLPKNLKPFRGTLSIHQVLWDILSERLTLRTKMPKVVYHYFACKALGESGRMLLAYGGQDFEDHRVLSADWPDFKPKTPFGQMPVLVIDGKQYAQSTAICRYLGRKYGLAGANDEEAFEIDQNVEFLHDIRAKAAAVYYEADEELKAKKHEDFSKNVYPDMLKKLNSIVEANKGHIAAGKLTWGDFVFTSMFDYLKTMLQIPDLEVQYPAFKKVLQSVLTQPKVKAFLDLGRPYEFEF | ^244^VLSADWPDFKPK^255^, ^256^TPFGQMPVLVIDGK^269^, ^375^TMLQIPDLEVQYPAFK^390^, ^404^AFLDLGRPYEFEF^416^ |

**Supplementary original and unprocessed figures.**

**
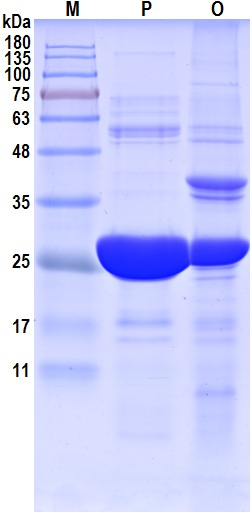
**

**Supplementary Figure S1. SDS-PAGE analysis of the proteins associated with the BmNPV polyhedra matrix (lane P) and ODV (lane O).** Polyhedra in infected cells were purified. The ODVs were released and pelleted by continuous sucrose centrifugation. Proteins of the ODV pellet and sample over the top gradient were resolved on SDS-PAGE and made visible using Coomassie brilliant blue staining. Lane M, the protein marker.


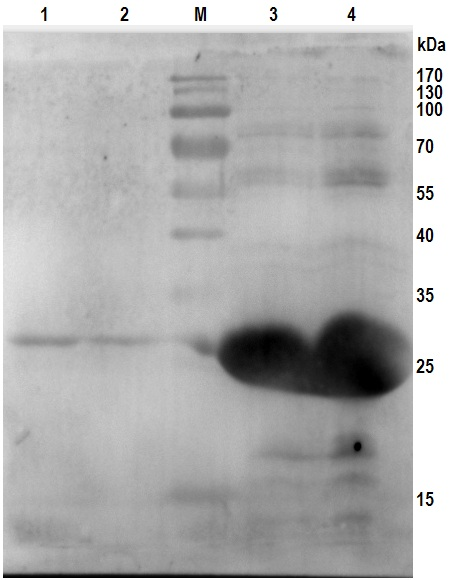


**Supplementary Figure 2A**. **Western blot analysis of HSP70-4 localization in the BmNPV polyhedra matrix.** About 1.0 (lane 1), 0.5 (lane 2), 5.0 (lane 3) and 10.0 (lane 4) μg of samples were loaded for SDS-PAGE and a Western blot analysis. Lane M, the protein marker.


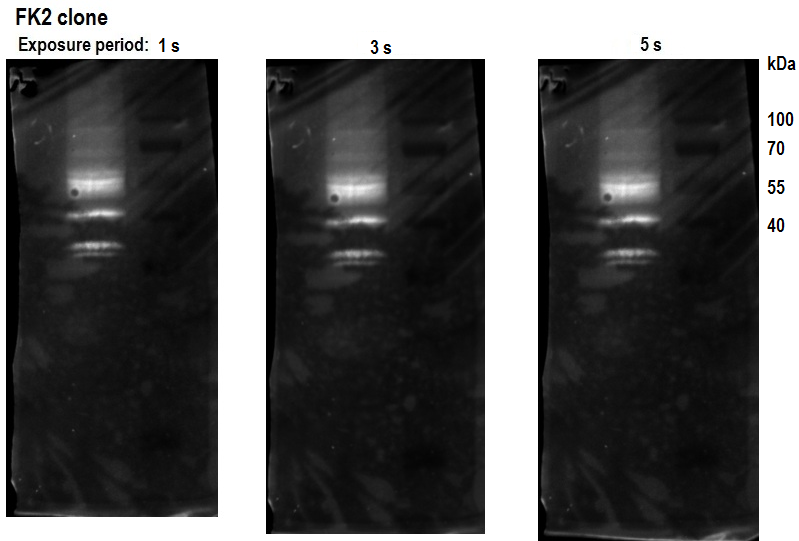


**Supplementary Figure 3A. Western blot analysis of ubiquitylated proteins in the BmNPV polyhedra matrix using a mouse monoclonal anti-ubiquitin antibody clone FK2.**


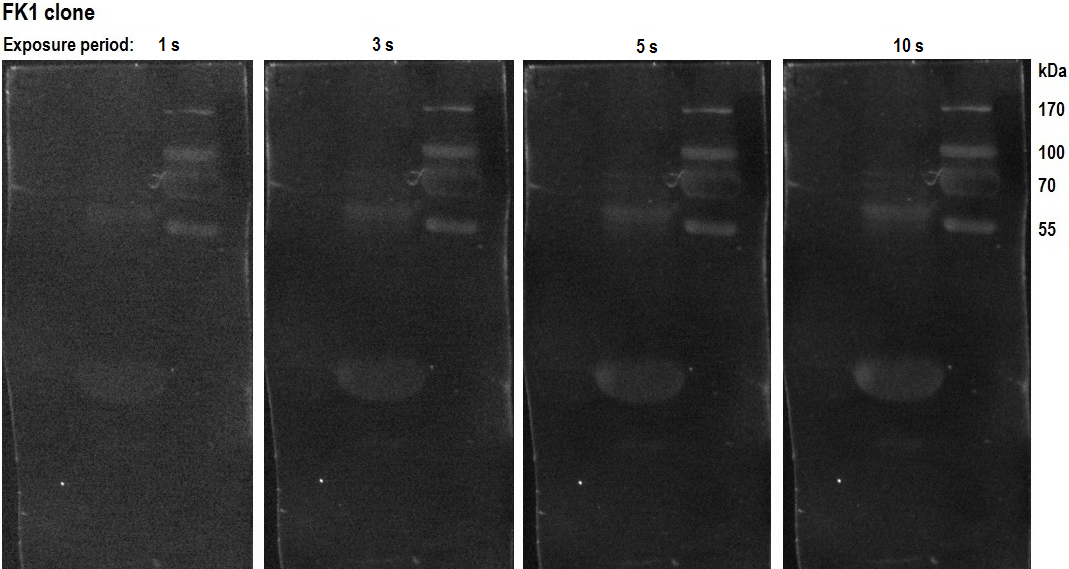


**Supplementary Figure 3B. Western blot analysis of ubiquitylated proteins in the BmNPV polyhedra matrix using a mouse monoclonal anti-ubiquitin antibody clone FK1.**


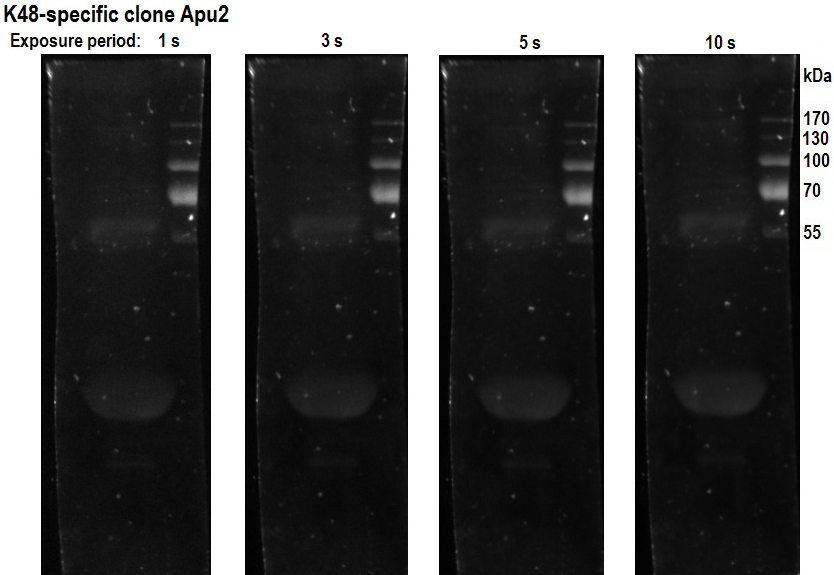


**Supplementary Figure 3C. Western blot analysis of ubiquitylated proteins in the BmNPV polyhedra matrix using a rabbit monoclonal anti-ubiquitin antibody Lys48-specific clone Apu2.**

**
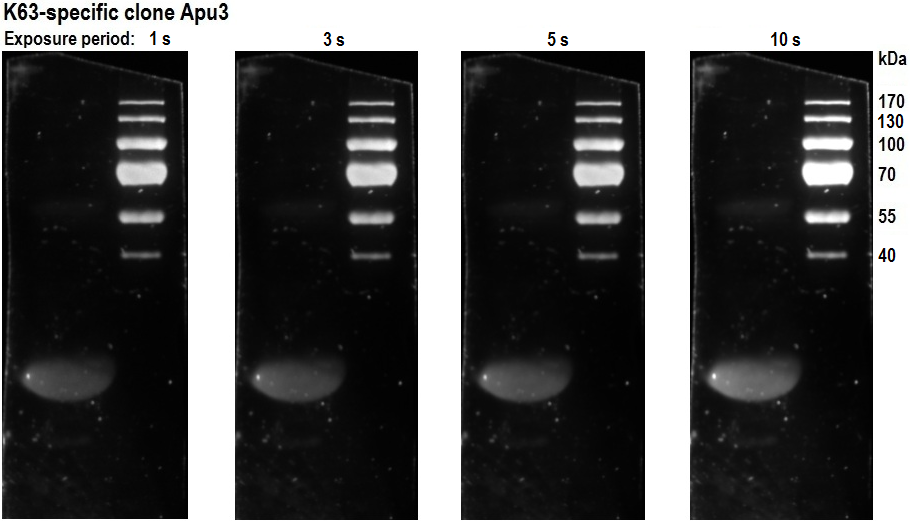
**

**Supplementary Figure 3D. Western blot analysis of ubiquitylated proteins in the BmNPV polyhedra matrix using a rabbit monoclonal anti-ubiquitin antibody Lys63-specific clone Apu3.**

**
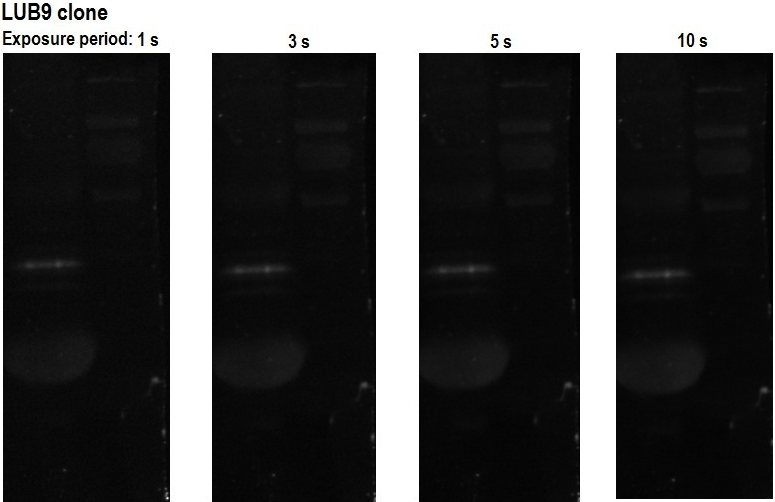
**

**Supplementary Figure 3E. Western blot analysis of ubiquitylated proteins in the BmNPV polyhedra matrix using a mouse monoclonal anti-linear poly-ubiquitin antibody clone LUB9.**


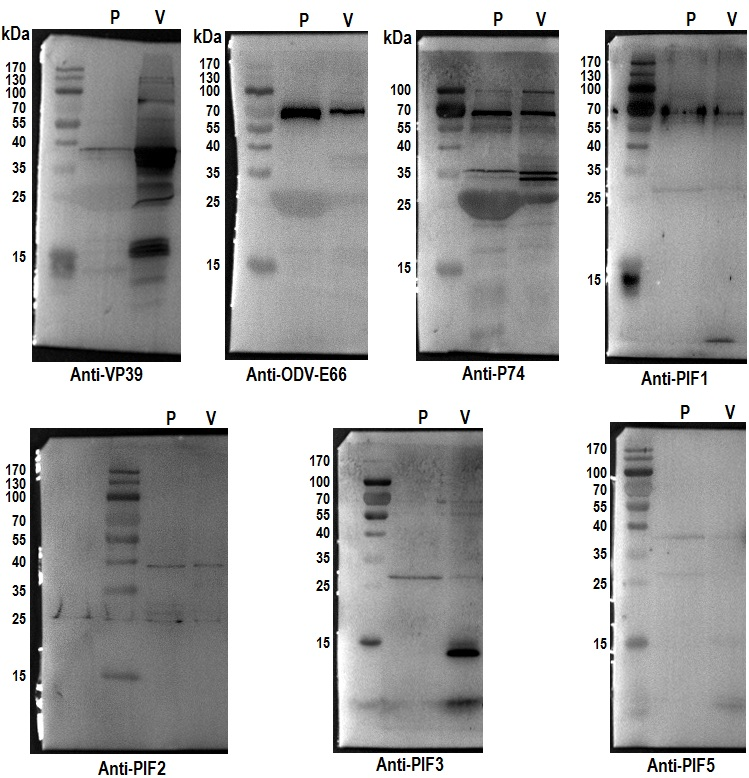


**Supplementary Figure 4.** **Western blot analyses of certain ODV-associated proteins in the BmNPV polyhedra matrix.** The polyhedra matrix (lane P) and ODV (lane V) fractions were loaded for Western blot analyses.
